# Cell Settling, Migration, and Stochastic Cancer Gene Expression Suggest Potassium Membrane Flux May Initiate pH Reversal

**DOI:** 10.3390/biom15081177

**Published:** 2025-08-16

**Authors:** Marie E. Beckner

**Affiliations:** Brain Health Research Institute, Kent State University, Kent, OH 44242, USA; mbeckner@sprynet.com

**Keywords:** potassium channels, potassium, *KCNJ16*, *KCNN4*, *KCNMA1*, H^+^ sensitivity, pH reversal, differential gene expression, Grotthuss proton transfer, oncogenes

## Abstract

Attraction of glioblastoma cells to potassium was suspected when glioblastoma cells clustered around dying cells and migrated towards serum (high [K^+^]) and increased potassium. Potassium channel proteins (KCN family, 90 members) mediating alterations in the transmembrane flux may provide K^+^ that releases H^+^ bound to inner membranes in cancer cells for cytosolic proton transfer, possibly conformational in water (Grotthuss), to extrusion sites. Cell settling and migration assay results led to collecting 70 studies, unbiased by the authors for inclusion of KCN genes, that detected KCN differentially expressed genes (DEGs). Of 53 KCN DEGs found among 29 malignancies, 62.3% encoded H^+^-sensitive proteins. KCN DEGs encoding H^+^-sensitive proteins were more prevalent in 50 studies involving one or more categories (seven oncogenes and histone/DNA modifiers) versus those with none; *p* = 0.0325. Pertinent genes for lactate outflow, etc., had relatively normal levels of expression. Brain tumors in REMBRANDT (database) showed altered expression of KCN genes encoding H^+^-sensitive proteins in glioblastomas versus less invasive oligodendrogliomas of patients on anti-seizure medications, with less *KCNJ16*/Kir5.1; *p* = 5.32 × 10^−8^ in glioblastomas. Altered H^+^-sensitive potassium flux via the KCN family, downstream of oncogenes and histone/DNA modifiers, putatively incites proton transfers for H^+^ release during pH reversal (pHi > pHe) in cancer.

## 1. Introduction

### 1.1. General

Potassium flux maintains cell membrane potential, assists volume changes, and permits neurons to fire action potentials, as the potassium they release is buffered by glial cells. Potassium is proposed to also exchange with fixed H^+^ on inner membranes to incite proton transfer and extrusion for tumor cell survival and invasion. Reversal of the pH gradient, i.e., intracellular pH (pHi) is greater than extracellular pH (pHe), may occur. K^+^/H^+^ cation exchange may explain the attraction of glioblastoma cells (highest-grade glial tumor) to axons in CNS white-matter tracts and peripheral nerves [1,2]. Cell morphology and viability in an experimental microaqueous environment suggested glioblastoma cell attraction to K^+^. Viable cells tightly clustered around single dying cells, putatively releasing cytoplasmic K^+^. Also, glioblastoma cells migrated towards fetal bovine serum with levels of 5–15 mM K^+^ [3]. The in vitro clustering behavior reflected how glioblastoma cells typically rim necrotic foci (pseudopalisading), often being band-like in vivo [4,5,6]. Pseudopalisading cells have a migratory phenotype [6,7]. Related pH features of benign astrocytic (glial) cells include (i) an increase in pHi with neuronal stimulation, possibly triggered by membrane K^+^ redistribution during depolarization [8,9]. Intracellular alkalinization of glial cells has been attributed to their uptake of potassium released by repetitive neuronal firing in vivo [10]. Also, (ii) glycolysis (inhibited by lactic acid) is stimulated by changes in intracellular [H^+^] rather than [HCO_3_^-^] in astrocytes [11]. Cytoplasmic alkalinization occurring via efflux of protons, resulting in extracellular acidosis, has been attributed to ion transporters, pumps, exchangers, etc. [12,13,14,15,16,17,18,19]. Redistribution of K^+^ has been suggested as contributing to tumor cell alkalinization [12]. Also, pH-sensitive K^+^ channels may be “sensors”, as described in pH reversal [13,14,16,17]. This study proposes that potassium flux plays an active role.

The KCN (potassium channel) family became the focus of a stochastic search for evidence of anomalous K^+^ flux based on its large size, complex family interactions, extensive non-family regulation, functional reliance on biological milieus, and a wide chromosomal distribution that increases their odds of genetic damage. Retrieval of data unbiased for KCN gene expression in tumors on differentially expressed genes (DEGs) of KCN family members and associated non-KCN DEGs in 70 studies of many cancer types provides a stochastic view of anomalous potassium flux. Also, a brain tumor database, REMBRANDT, and genome analysis of six glioblastoma cell lines were queried for KCN genomic data [20,21,22]. The detected H^+^ sensitivity of a large proportion of KCN family members and high likelihood of K^+^ as a replacement cation for H^+^ suggest that potassium ion flux across plasma membranes or accumulation near them plays a role in pH reversal in malignancy. Deregulated, inward channel flux or loss of diffusely distributed outward K^+^ flow (necessitating focally concentrated outward flow), leading to accumulations of K^+^ ions near H^+^ bound to anions of inner cell membranes, potentially leads to K^+^/H^+^ exchange that releases H^+^ as an initial step in pH reversal.

### 1.2. Cytosolic Proton Transfer Related to K^+^ and Hydration

Potassium replacement of inner-membrane-bound H^+^ should augment hydronium ion (H_3_O^+^) accumulation seen at water–lipid interfaces [23]. This putatively sets the stage for pH reversal. The mobility of unbound H^+^ (H_3_O^+^ at 36.2 × 10^−4^ cm^2^ V^−1^s^−1^) is high compared to K^+^ and Na^+^ [24]. In immortalized embryonic kidney cells, H^+^ diffusion through the cytosol occurred 60-fold faster than when limited to the inner plasma membrane, i.e., 0.6 μms^−1^ versus 0.01 μms^−1^ [25]. In cardiac myocytes, the cytoplasmic H^+^ distribution speed, 0.7 μms^−1^, was comparable based on diffusion using HCl in KCl solutions with membrane acid transport inhibited [26]. Also, dissociation of HCl occurred in a microaqueous environment, with the acid’s protons transferred to a neighboring water molecule and then another in clusters of HCl(H_2_O)_n_, where n = 1 to 5 and 7. With addition of a fifth and seventh water molecule, the covalent H-Cl bond transitioned to the contact ion pair, H_3_O^+^Cl^−^, plus free hydrogens. HCl(H_2_O)_5_ and HCl(H_2_O)_7_ clusters were spontaneously dissociated structures with contact ionic bonds [27]. Thus, H_3_O^+^ may be distributed to the cytosol after putative K^+^/H^+^ exchange on inner membranes and after dissociation of acids in cytosols, possibly microaqueous.

Cytosolic proton transfer has been attributed to taurine, carnitine, carnosine, or homocarnosine behaving as mobile buffers [26]. However, proton transfers from H_3_O^+^ may also occur by a rapid mechanism consistent with behavior described by C.J.T. Grotthuss in 1806 [28]. Proton transfers occur via H-bonded complexes, H_9_O_4_^+^ and H_5_O_2_^+^, or by wires of protonated water molecules linked by interconversions, consistent with behavior of salts and water that Grotthuss observed between electrodes. With these interconversions, a proton that enters a water matrix is not the same proton that exits elsewhere [24,28,29,30,31,32,33,34]. Conformational changes for proton transfer in water occur as a rapid falling-domino effect for wide-reaching delivery of cytoplasmic protons to membrane extruders. Bending of hydrogen bonds to accommodate acceptance of a new proton quickly ripples through a properly aligned water matrix to release another proton distally while snapping back to normal to be ready for the next proton that enters. Hydrogen-bonded water chains mediate proton transfer much faster than diffusion. Rapid transfer of H^+^ in water is also known as a “proton jump” [35]. H^+^ can be transferred to cell exit sites, such as exchangers swapping out H^+^, monocarboxylate transporters venting lactate that requires accompanying protons, exiting through gap junctions, etc. Although the Grotthuss mechanism may not mediate extracellular tumor acidity, it was not ruled out as occurring intracellularly [36]. The Grotthuss mechanism describes an “everywhere, all at once” strategy for delivery of protons to cell exit sites. Therefore, inner-membrane proton exchange with K^+^ is probably highly regulated in normal cells, consistent with K^+^ flux via the large, complex KCN family. Loss of regulation, reflected by differential expressions of KCN genes, allows assessment of potential ionic dynamics related to pH reversal in malignant cells. Genetic damage of KCN family members or altered regulation by oncogenes, modified histones/DNA, etc., putatively plays a role in pH reversal to aid survival of glycolytic cells producing lactate that is not removed via glycogen formation.

Also, loose aggregation of water molecules with potassium is important. Lack of water due to poor circulation, cell crowding, and pressure from fibrotic, stiff surroundings, especially during invasion, produces microaqueous conditions within cancer cells. However, regional hydration may occur by release of water molecules from K^+^-7H_2_O to join protonated water structures close to cell membranes, but this is unknown.

Volume changes related to ions occur in cells during division and migration. Protrusion of pseudopodia (increased volume in cell projections) and shrinkage of their retracted rear cell bodies occur with oscillations of cell membrane potential and osmotic changes via KCl uptake and release through potassium and chloride channels, with aquaporin participation and possibly K^+^ refilling/recycling [37,38,39,40,41,42]. Thus, cell migration produces ion and hydration dynamics that may influence proton transfer at the leading edge of migrating cells. Pseudopodia are glycolytic, based on proteomic analysis [43]. Also, enhanced proton release leading to extracellular acidosis aids tumor invasion via activation of metalloproteases for matrix degradation [44,45,46,47,48,49,50]. Tumor cells’ escape from poorly perfused, microaqueous tissue may be aided by K^+^/H^+^ exchange initiating pH reversal.

### 1.3. Need for Proton Extrusion in Cancer

Metabolism of cancer cells produces excess protons [15,51]. In addition to the acids produced by metabolic pathways, free radicals generated are also acidic [52,53,54]. Extrusion of protons avoids cytoplasmic acidosis, so this not only protects glycolysis but also prevents cytochrome c-mediated activation of caspase-3 to stop apoptosis [55], protects gap junctions [56,57,58,59], and activates ATP citrate lyase (ACLY), optimal pH of 8.5–8.7 [60,61]. ACLY’s removal of citrate (inhibitor of glycolysis) is protective in glioblastomas [62]. Also, by generating acetyl-CoA from citrate, nuclear ACLY promotes histone lysine acetylation, so that its positive charge is removed to allow loosening of chromatin that drives expression of genes for cell adhesion, migration, and integrin signaling for cancer cell invasion [63,64]. Thus, proton removal from the cytoplasm favors tumor cell fitness as well as enabling migration to escape adverse conditions [64,65].

### 1.4. Complexity of K^+^ Flux

The direction and strength of K^+^-channel flow occurs largely via the KCN family, encoded by 90 genes [66,67]. Also, numerous non-KCN regulators are present in biological milieus, such as polyamines, voltage, H^+^, Ca^2+^, Mg^2+^, Zn^2+^, Na^+^, ATP, miR-133b, reactive oxygen species (ROS), reactive nitrogen and sulfur species, nitric oxide, hydrogen sulfide, hypoxia, hyperglycemic glucose, nucleotides, protein kinases A, C, and G, guanosine-triphosphate-binding proteins (G-proteins), regulator of G protein signaling (RGS) proteins, phosphatidyl inositol 4,5 bisphosphate, estrogen, testosterone, cholesterol, sulfonylurea, β-hydroxybutyrate, hypoxia inducible factor-1α, 14-3-3 protein, β-coat protein, dipeptidyl aminopeptidase-like proteins, GABA, NAD(+), NADP(+), NADH, NADPH, glutathione, glutathione disulfide, mechanical stimulation, cell cycle progression, products of *LINGO1,2*, and BKγ1-4, encoded by *LRRC26*,*52*,*55*,*38* [68,69,70,71,72,73,74,75,76,77,78,79,80,81,82,83,84,85,86,87,88,89,90]. Also, 19% of proteins targeted by small-molecule drugs used to treat numerous diseases are ion channels [91,92,93]. ROS are generated via mitochondrial respiratory chain electron transport redox reactions and the NOX family of NADPH oxidases [71,84,94,95,96,97,98]. Colon carcinoma cells with acid-resistant growth showed upregulated expression of *NOX1*/NADPH oxidase 1 [99]. Interestingly, isolated respiring rat brain mitochondria increased ROS production when their medium was alkalinized [100].

The widespread chromosomal distribution of KCN genes and non-family regulators suggests failsafe redundancies exist for genomic damage to maintain levels of K^+^ for survival, without being optimal for differentiation, in dividing tumor cells. Given that monocarboxylate transporters, MCT1 and MCT4, transport negatively charged lactate molecules coupled one-for-one with protons across cell membranes [101] and protons can also be extruded via Na^+^/H^+^ exchanger family members, ATP driven pumps, etc. [12,13,14,15,16,17,18,19,102], there are pertinent genes among the non-KCN DEGs that are also of stochastic interest.

Mediators of potassium flux influencing the relationship of K^+^ and H^+^ at cell membranes should be clarified. K^+^ accumulation due to a lack of normal diffuse K^+^ efflux or buildup of K^+^ around large outwardly conducting potassium channels, could putatively exchange with bound protons on inner membranes or it could happen with deregulated inwardly channeled K^+^ flux (possibly with K^+^ being recycled for refilling as needed if efflux becomes excessive). These events with K^+^ could occur without affecting the functions of key structural proteins that would be seen if Na^+^ was used [103]. Although both potassium and sodium are available to replace membrane-bound H^+^, there should be a preference for potassium due to its weaker binding of water, so that less energy is required to displace water from potassium [104,105]. K^+^ ions permeate their membrane channels in a desolvated (‘naked’) state rapidly, so at this point they release their associated water molecules. There is exclusion of smaller Na^+^ ions, which have stronger water binding [106,107,108]. Also, compared to Na^+^, K^+^ is better at maintaining solubility of cell components containing carboxylates and phosphatesto help explain its higher concentration in the cytoplasm [105,109]. Numerous KCN subfamilies may be involved with anomalous potassium flux. Deregulated diffuse, passive K^+^ entry via the *KCNJ*/Kir (inwardly rectifying, also bidirectional, flux) or disrupted K^+^ outflow via numerous Kv (voltage dependent) KCN subfamily and *KCNK*/K2P subfamily (tandem two-pore “leak”) members are important. Also, Ca^2+^ activated potassium channel family members that mediate outward flow, *KCNMA1*/BK, and beta subunits that regulate it, *KCNN*/SKs, and *KCNN4*/IK, with BK and IK providing large and medium flux, respectively, likely play important roles. Higher flow rates may provide compensatory localized K^+^ release and are likely affected by release of calcium from cancer cells. However, all types of K^+^ transits may be involved, and a role for Na^+^ in releasing H^+^ cannot be ruled out given the high levels of Na^+^ in some tumors [110,111,112]. A few K^+^ channels are influenced by Na^+^.

Loss of normal cellular responses to pH via loss or gain of KCN channels that function with H^+^ sensitivity may alter cancer cells. Glioblastoma cell behavior suggests an opportunistic response to sources of K^+^, thus achieving levels of K^+^ ions at innermembrane surfaces that promote K^+^/H^+^ exchanges to initiate pH reversal for cell survival and invasion. These sources include (i) neurons and axons firing action potentials where K^+^ is released to maintain electroneutrality, (ii) dying cells, including red cells (K^+^, 80–120 mmol/L) and necrotic tissue releasing cytoplasmic K^+^ [113,114,115,116], and (iii) damaged blood vessels leaking plasma (potassium, 3.8–5.0 mmol/L [117]). Deregulation via loss of H^+^-sensitive *KCNK1* (among a small group of genes identified) was advantageous for breast cancer cell survival of induced necrosis [118].

### 1.5. Biological Milieu Constituents to Consider in Studying K^+^ Flux

Relevant details of milieu constituents in studying KCN channels are listed. Serum includes calcium (2.30–2.74 mmol/L), magnesium (0.74–1.23 mmol/L), phospholipids (1.50–3.80 g/L), sodium (136–142 mmol/L, adult plasma value) [117], and polyamines (0.1–1.0 μM, fetal bovine serum [3]). Magnesium and polyamines (positively charged) are important gating molecules of inwardly rectifying *KCNJ*/Kir-family channel members [119]. Removing fetal bovine serum from fibroblasts resulted in decreased intracellular potassium [120]. Also, fetal bovine serum lost the ability to depolarize the resting membrane potential of airway smooth muscle cells after dialysis against distilled water but its freeze-dried dialysate retained this ability after reconstitution [121]. Elevated levels of polyamines occur in cerebrospinal fluid of patients with brain tumors [122] and in tumor-associated myeloid cells infiltrating glioblastomas [123]. Also, polyamines are formed when L-arginine, released from apoptotic bodies, is metabolized by Arg1 [124]. Other modifiers within necrotic tissue include hemoglobin breakdown products [125,126]. Tumor-associated macrophages are influenced to promote malignancy (increased expression of *ARG1, VEGF*, *TGFB*, etc.) by lactate, presumably shuttled from cells of bladder and gastric cancers, and others [127,128,129].

As mentioned earlier, many non-CNS tumors exhibit perineural invasion [130,131,132,133]. There may be reciprocal gain of potassium by tumor cells from nerves and axons as the tumor cells provide lactic acid back to them as an energy substrate for a mutually beneficial interchange. Although reports of specific sources of K^+^ affecting behavior of cells in vivo have not been found, opportunistic sources of potassium may be important for dedifferentiated cancer cells.

### 1.6. Findings Highlighted in This Study

Fetal bovine serum (with and without hepatocyte growth factor (HGF)) promoted glioblastoma cell migratory behavior, including protrusion of pseudopodia through 3 μm filter pores, migration of whole cells through 8 μm filter pores, and invasion through Matrigel and rat brain tissue slices, often under glycolytic conditions [43,62,134,135,136]. Glioblastoma cell clustering around dying cells and their migration to serum, with compelling background information on astrocytes, suggested involvement of anomalous potassium flux in tumor cell behavior. Cell migration of multiple glioblastoma cell lines to serum among the eight tested in this study, and the response in a separate assay to a positive gradient of potassium, suggested abnormal membrane flux as supporting tumor cells. The large number of K^+^-channel candidates for loss or gains in expression under potential influence of numerous modulators led to stochastic analysis of published studies for unbiased data comprised of incidentally detected KCN DEGs and accompanying non-KCN DEGs in genomic landscapes of cancer scenarios and databases. This permitted a search for pertinent functional similarities among KCN DEGs. Meta-analysis of 70 malignant (or related models’) expression studies found that the studies fell into eight categories of seven oncogenes and histone/DNA modifiers. These categories were determined by the authors of the expression (E) studies, E1–E70, if related DEGs were also detected in any of the 70 studies. The studies included 29 types of malignancy (breast, lung, brain, etc.) that incidentally detected KCN DEGs; *KCNMA1* and *KCNN4* were the most frequent. The encoded products of the 53 detected KCN DEGs are predominantly H^+^-sensitive (62.3%), and 52 (74.3%) of the 70 studies identified at least one H^+^-sensitive KCN DEG. There was a significant propensity for KCN DEGs to encode H^+^-sensitive family members in studies among the eight categories of seven oncogene and histone/DNA modifier-related scenarios compared to 20 studies in “no category”. A concurrent search of the National Institutes of Health Repository of Molecular Brain Neoplasia Data (REMBRANDT) database for expression levels of KCN genes in invasive glial tumors (glioblastomas versus oligodendrogliomas that are less invasive) of patients on anti-seizure medications showed a significant decrease in *KCNJ16* and trends in three other KCNs also encoding H^+^-sensitive proteins in glioblastomas. Correlation with a genomic study of six glioblastoma cell lines [22] confirmed relatively decreased RNA expression of *KCNJ16* among KCN genes and provided additional data. Altered expression of KCN family members, possibly compensatory, and non-KCN factors may provide quantities of K^+^ sufficient for preservation of cell membrane integrity and K^+^/H^+^ exchange on inner membranes to release bound H^+^ for pH reversal and invasion. The aim of this study was to search for potassium’s functional relationships in cancer.

## 2. Materials and Methods

### 2.1. Materials and Cell Culture

Chemicals and reagents were from Sigma-Aldrich (RRID:SCR_008988), St. Louis, MO, USA, unless otherwise stated. Anti-Met was from Cell Signaling Technology, Beverly, MA, USA, Cat# 4560, RRID:AB_2143887), 1:1000 dilution, for the beta Met subunit (145 kDa). Diff Quik (Allegiance, McGaw Park, IL, originally Harleco, now Sigma-Aldrich’s Hemacolor Solutions I, II, III [137]. A172 (RRID:CVCL_0131), C6 (TKG Cat# TKG 0242, RRID:CVCL_0194), F98 (ATCC Cat# CRL-2397, RRID:CVCL_3510), LN18 (RRID:CVCL_0392), LN229 (RRID:CVCL_0393), T98G (RRID:CVCL_0556), U87 (RRID:CVCL_0022), and U373 (RRID:CVCL_2219) glioblastoma/astrocytoma cell lines (human (A172, LN18, LN229, U87, U373) and rat (F98 and C6)) were obtained from the American Type Culture Collection, Manassas, VA, USA, RRID:SCR_021346. No cell lines were generated. Cells were maintained in Minimal Essential Media (MEM) with Eagle [138] salts (Cellgro, Mediatech, Herndon, VA, USA) with 10% fetal bovine serum, FBS, (Invitrogen, Carlsbad, CA, USA).

### 2.2. Statistics

Pearson product-moment correlation, R, (*p* values for true correlation > 0), Student’s t-Test, paired and unpaired, and Fisher’s exact test for count data were used in Excel Statistics (RRID:SCR_017294) or R Project for Statistical Computing, v4.3.1 (RRID:SCR_001905).

### 2.3. Databases

For gene information, the National Institutes of Health, https://www.ncbi.nlm.nih.gov, Online Mendelian Inheritance in Man (OMIM) (RRID:SCR_006437), https://omim.gov, GeneCards (RRID:SCR_002773) www.genecards.org (including protein interactions), and Ensembl https://www.ensembl.org/ (RRID:SCR_002344) databases were used. The National Library of Medicine’s PubMed database, https://pubmed.ncbi.nlm.nih.gov/, was searched for numbers of publications, using word searches for subjects of interest. OMIM was searched for KCN genes expressed in the nervous system. The Repository for Molecular Brain Neoplasia Data (REMBRANDT) (RRID:SCR_004704), NIH, included in the Georgetown Database of Cancer (G-DOC), https://sites.google.com/georgetown.edu/g-doc/home or the G-DOC Hub platform, https://gdochub.georgetown.edu/, a project of the Georgetown Lombardi Comprehensive Cancer Center, Washington, D.C., was used to provide translational research tools [20,21]. It was queried for gene expression of KCN genes, selected cancer genes, and housekeeping genes (HKGs). Medians of gene reporter probes in Adobe Flash (RRID:SCR_017258) were available through 31 December 2020. The genomic results of six glioblastoma cell lines were obtained from the supplemental materials of Patil et al. [22].

### 2.4. Cell Settling Assays

Human glioblastoma cells (U87, LN229, A172, T98G) maintained in culture conditions were detached with trypsin on the day of assay, and allowed to recover for 4 h in MEM with 10% FBS at 37 °C in a 5% CO_2_ incubator. For each assay, a Cell Settling Chamber Kit (Neuro Probe, Gaithersburg, MD, USA), with 8 wells, 7 mm diameter, was assembled. A provided glass slide, 25 × 75 mm, was placed in the recessed bottom plate with pre-punched (for wells) blotting paper, pre-moistened with PBS, a gasket, and the upper plate stacked on top. Thumbnuts secured the top plate. Centrifuged (<500 rpm, 5 min) cells were resuspended with gentle pipetting; 500,000 per ml in culture media with 10% FBS. Cell suspensions were added, at 50 μl per well. After 30 min, with media visibly absorbed by blotting paper, the chamber was disassembled, and the slide was removed and stained with Diff Quik followed by rinses in tap water. At least two replicate wells per cell line were prepared. The stained cells were examined microscopically. The cell vacuoles in U87 cells (single and clustered) were counted in settled monolayers. When the vacuoles collectively had a diameter of at least one third of an average nuclear diameter in the same 100× microscopic field, the cell was positive for vacuolization (surrogate of degeneration). The cells were analyzed in a midline sweep of 100× microscopic fields across each well. Pearson product-moment correlation coefficient, R, determinations were performed for the percentage of cells vacuolated versus the number of cells in each cluster. Morphology, ,including cell clustering, was noted. An Olympus BH2 microscope (Olympus Corp., Lake Success, NY, USA) with an Olympus FV II digital camera, using the Olympus Microsuite Five Software for Imaging Applications (Soft Imaging System, Lakewood, CO, USA), was used to capture black and white images. An American Optical Spencer Microscope (Buffalo, NY, USA, now Leica Microsystems, Inc. (Deerfield, IL (RRID:SCR_008960)) with an OptikamB9 Digital Camera 10 MegaPixels (Optika, www.optikamicroscopes.com, Via Rigla 32, 24010 Ponteranica (BG), Italy) with Adobe Photoshop v5.1 and Adobe Photoshop Elements 2024 for Windows (Adobe Systems Incorporated, San Jose, CA, USA (RRID:SCR_014199)) was used to generate color images.

### 2.5. Glioblastoma Cell Migration Assays

Glioblastoma cell migration assays have been described previously [62,135,136]. The A172, C6, F98, LN18, LN229, T98G, U87, and U373 glioblastoma/astrocytoma cell lines were maintained in culture conditions with half volumes of media changed the day before each assay. Cells were detached with trypsin on the day of the assay and allowed to recover in media with 10% FBS for 2 h at 37 °C in a 5% CO_2_ incubator. Centrifuged (<500 rpm, 5 min) cells were resuspended 2 million/mL with gentle pipetting in media for migration. Unless otherwise stated, it was MEM (salts include 5.4 mM KCl [138] but it is 5.3 mM according to Thermo Fisher) with 0.1% bovine serum albumin (BSA). Chemoattractant/chemokinetic stimulants, hepatocyte growth factor (HGF), and/or FBS in MEM or PBS media were added to the bottom wells (29–30 μL total volume) as indicated. Then, 48-well modified Boyden chambers (Neuro Probe) were assembled with porous (8 μm, 1000 pores/mm^2^), air-dried polycarbonate filters, 7 μm thick (Neuro Probe), coated with 0.01% porcine gelatin (solution of 1 mL 10% acetic acid, 1 mL 1% gelatin (from 10% refrigerated stock), and 98 mL H_2_O). One corner of each filter was cut for orientation before assembly. Cell suspensions, 60 μL, were added to each upper well. The chamber was incubated in a large, covered petri dish with a small amount of water at 37 °C for 5 h in a 5% CO_2_ incubator. Upon assay completion, the filter was removed and attached to a large clip and stained with Diff Quik, rinsed in 3 beakers of water, and placed on a glass slide (2” × 3”), with migrated cells on the bottom side of the filter. Unmigrated cells were wiped from the upper filter surface with cotton swabs or Kimwipes with only stained migrated cells remaining, trapped between the filter and slide. There were 4 replicates per data point in most assays. Slides with stained migrated cells on the filters were digitized (HP OfficeJet Plus 8715 printer/scanner (Hewlett Packard (formerly RRID:SCR_011873), Spring, TX, USA). Densitometry (UN-SCAN-IT Gel Analysis Software, 7.1 (RRID:SCR_017291) Silk Scientific, Inc., Provo, UT, USA) with correction for background filter staining was used to quantify cell migration. An Epson Perfection 2450 PHOTO (Epson America, Long Beach, CA, USA) was used for obtaining some images of the assays. The first two rows of migrated cell pellets, digitized, were used to calculate HGF cell migration, i.e., second rows of cells stimulated by HGF divided by the first rows of unstimulated cell migration (background), stated as times (X) background cell migration for each cell line. Additional migration assays of U87 cells evaluated motogenic/chemokinetic properties of FBS and HGF by placing them in either the bottom or both top and bottom wells. Separately, the use of MEM versus Dulbecco’s PBS in the bottom wells in a dose curve of FBS (including no FBS for MEM versus PBS) was evaluated with Migration media (Dulbecco’s PBS (contains 2.7 mM KCl, 1.8 mM KH_2_PO_4_, 137.9 mM NaCl, 10 mM Na_2_HPO_4_) with added 0.210 mM MgCl_2_, 0.243 mM CaCl_2_, 5 mM pyruvate, 5.551 mM glucose, and 0.1% bovine serum albumin) in all top wells.

### 2.6. Immunoblot of Met, Receptor for HGF

One-dimensional gel electrophoresis was performed on lysates from 8 cell lines, equalized for protein (10 μg per lane), in 10% polyacrylamide gels under reducing conditions. The protein standards (MagicMark, MultiMark, Invitrogen (now Thermo Fisher Scientific, Waltham, MA, USA (RRID:SCR_008452)) were 8 μg per lane. The gel was stained with Coomassie blue (Novex Colloidal Blue Stain Kit, Invitrogen, now Thermo Fisher Scientific (RRID:SCR_008452)), photographed, and destained with water. The gel contents were transferred to polyvinylidene difluoride membranes (Invitrogen, now Thermo Fisher Scientific (RRID:SCR_008452)), blocked (Detector Block, Protein Detector Western Blot Kit Lumi-GLO System, Kirkegaard & Perry Laboratories, Gaithersburg, MD, USA, now SeraCare Lifesciences:(RRID:SCR_004535)), and reacted with anti-Met (1:1000), reactive 145 kDa band. The secondary antibody 1:1000 (Kirkegaard & Perry Laboratories, now SeraCare Lifesciences:(RRID:SCR_004535)) was horseradish peroxidase-labeled anti-mouse. Immunoreactive bands were visualized via a luminol-based solution that produced chemiluminescence, and the blot was scanned (Epson Perfection 2450 PHOTO) for densitometry using UN-SCAN-*It* gel, v5.1.

### 2.7. Differentially Expressed Genes of Potassium Channels

Potassium channel (KCN) family members were found as differentially expressed genes (DEGs) in genomic landscapes described in 70 cancer studies (1301 authors, 15 journals, 2007–2024). KCN DEGs were detected with non-KCN DEGs in at least one figure (heatmap, Venn diagram, volcano plot, etc.) or table (none from Supplementary Materials). The authors had no initial focus on KCN genes. All issues of *Cancer Research* (2014–early 2024), *Journal of Molecular Diagnostics* (2020–2024), and *Molecular Cancer Research* (2023) were searched visually for papers reporting KCN DEGs. Also, “heatmap” or “heat map” and notation of “open access” were used for cancer articles in archives of *Nature Communications* (2015–2023) and *Cancer Cell* (2011–early 2023) to select papers for visual detection of KCN DEGs. Fifty-nine of the 70 studies were systematically selected and others were incidental (files and online listserv notifications). The studies included malignancies (or models) of breast, lung, gastrointestinal, brain, bone marrow, etc. Categories (determined after all E1–E70 studies were selected) were according to descriptions by the authors if there was also a relevant DEG reported in at least one of the E1–E70 studies.

### 2.8. Expression of Potassium Related Genes in Glial Tumors

REMBRANDT (G-DOC) contained tumor expression data on 67 oligodendroglioma (Oligo) and 220 glioblastoma (GBM) patients, including subgroups treated with anti-seizure (AS) medications, i.e., 24 Oligo-AS and 75 GBM-AS. Gene expressions were determined by reporter probes on microarrays, with median values provided. Differences in median values for multiple probes (when available) were found with *t*-tests, paired via reporter probes for the two comparison groups when possible, and otherwise, unpaired *t*-tests were performed. Housekeeping genes (HKGs) that lacked relevance for glial tumors were analyzed for comparisons. Additionally, Oligo-minus-GBM (OMG) differences were calculated for each probe. OMG differences for each gene of interest were compared to OMG differences for HKGs (*B2M*, *PPIA*, *RRLP0*, and *YWHAZ*). In OMG comparisons, the *t*-tests were for unpaired samples with unequal variance. Published studies [22,139] were used for confirmation.

## 3. Results

### 3.1. Settled Glioblastoma Cells with Fluid Removed

**Figure 1:**
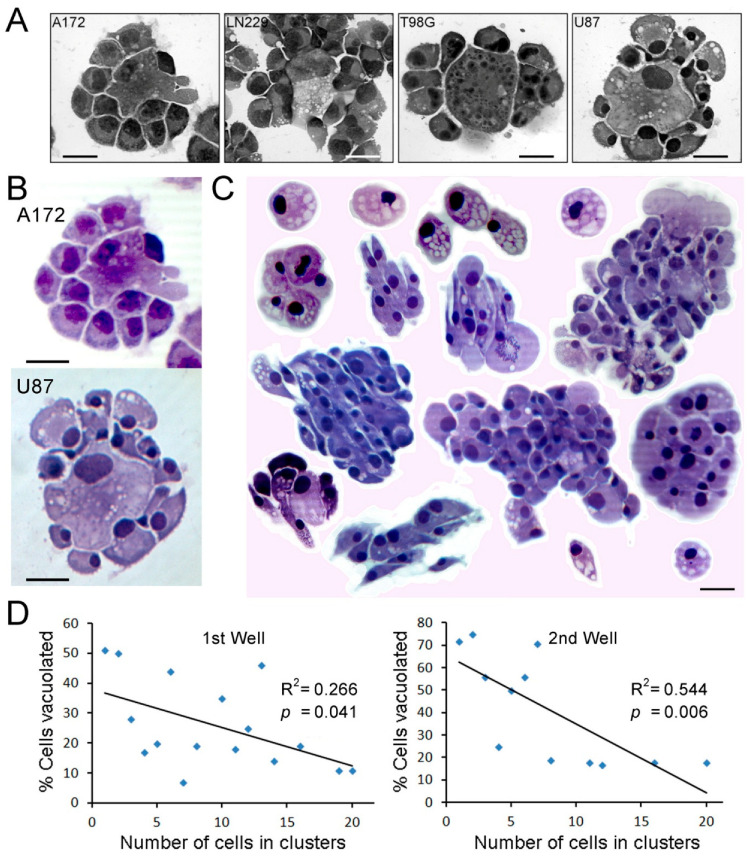
Settled glioblastoma cells deprived of extracellular fluid. (**A**,**B**) The morphology of each cell line (A172, LN229, T98G, and U87) after cell settling with fluid wicked away, included a few clumps of robust appearing, viable tumor cells clustered around single degenerating cells of the same lineage. Although Diff Quik staining varied, some viable clustered cells’ cytoplasm stained basophilic. Black and white photographs suggest some thin strands between cells crossing narrow linear spaces separating them. Single and grouped cells without a visible degenerating central cell were also seen. (**C**) Cytoplasmic vacuolization in U87 cells is shown in representative single and grouped cells, outlined digitally with white strokes around each merged layer. The basophilic cytoplasm seen in some cells would be consistent with an alkaline pH, but no measurements were made. Numbers of vacuoles were highest in single cells and decreased as numbers of cells in cellular groups increased. (**D**) For counts performed on cell monolayers from two separate wells, 51% of 75 and 72% of 25 single cells were vacuolated,1st and 2nd wells, respectively. However, vacuolated cells in groups were only 11% of 122 and 18% of 39 cell groups with greater than twenty cells, 1st and 2nd wells, respectively. Pearson regressions of the numbers of cells in groups with vacuoles had R^2^ values of 0.266 (*p* = 0.0410) and 0.544 (*p* = 0.0062), 1st and 2nd wells, respectively. Diff Quik stain. Scale bars, 25 μm.

**Figure A1:**
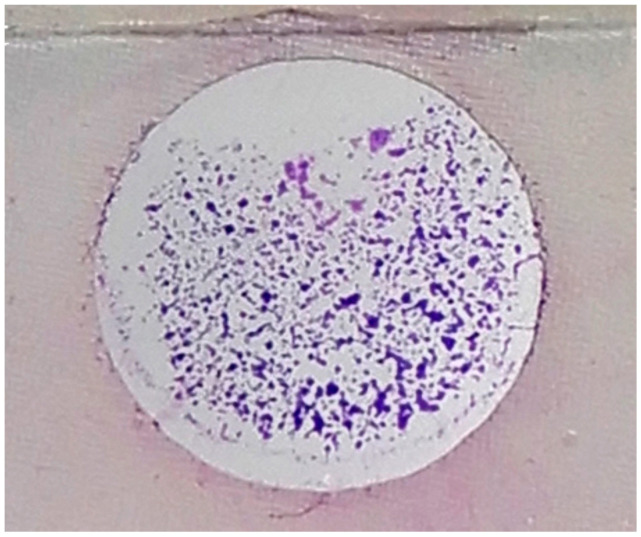
Macroscopic view of settled T98G glioblastoma cells on a Neuro Probe microscopic slide. One of 8 round 7 mm diameter areas that had been the bottom of a settling chamber well. Some cell clumping is noted. Diff Quik stain.

At the end of each cell settling assay, the extracellular fluid had been wicked away. Cell groups were viewed macroscopically; see Appendix A (Figure A1). For each cell line (A172, LN229, T98G, and U87), a striking feature was clustering of robustly viable tumor cells around single degenerating cells (same lineage) in low numbers. Tight clustering of viable cells suggested an opportunistic attraction to dying single cells, possibly their released contents. Some viable cells clustered around single dying cells stained basophilic (Figure 1A,B). Narrow linear spaces with a few thin strands connecting cells were seen in black and white photographs. Cells, single and in groups, without visibly dying centralized cells were also seen. Cytoplasmic vacuolization was most prominent in U87 cells (Figure 1A–C). Vacuole numbers were highest in single cells and decreased as cell clusters increased in size. Representative single and grouped U87 cells, layered together with white strokes around each layer for delineation, are shown in Figure 1C. For counts of vacuolated cells in two separate wells, 51% and 72% of single cells were vacuolated, whereas vacuolated cells accounted for only 11% and 18% in cell groups of greater than twenty cells, first and second wells, respectively. Pearson regressions of cells in clusters with vacuoles had significant R^2^ values of 0.266 (*p* = 0.0410) and 0.544 (*p* = 0.0062), first and second wells, respectively, as shown in Figure 1D. Cells in clusters exhibited less vacuolization/degeneration, possibly due to sharing cytoplasmic contents and contributions of contents from dying cells.

### 3.2. Cell Migration with HGF and Serum Chemoattraction

**Figure 2:**
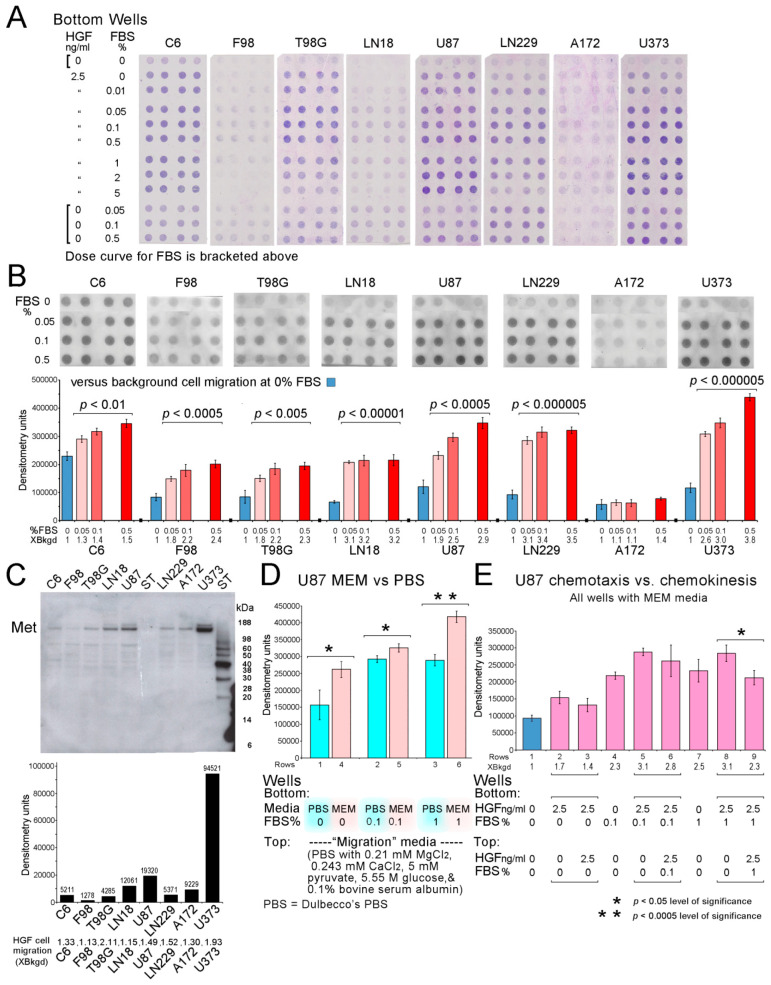
Migration of glioblastoma cell lines to hepatocyte growth factor (HGF) and fetal bovine serum (FBS). (**A**) Representative Boyden assays of 2 rat astrocytoma (C6 and F98) and 6 glioblastoma cell lines (T98G, LN18, U87, LN229, A172, and U373) showed background (unstimulated) cell migration (row 1), cell migration to HGF (row 2), HGF and FBS (rows 3–9), and to FBS alone (rows 10–12) using concentrations in the bottom wells as indicated on the left. With the same amount of HGF present, adding increasing amounts of FBS increased chemotactic cell migration in all cell lines except A172. Images were obtained with a transparency scanner. (**B**) Portions of assays (regular scanner) with FBS alone as the chemoattractant were digitized for densitometry, with results (average total pixels) shown. All cell lines showed significant migration to FBS except A172, which only had a trend, *p* = 0.0638, for 0.5% FBS. All assays (3 per cell line) are in Appendix A, for C6, F98, T98G, and LN18 (Figure A2) and U87, LN229, A172, and U373 (Figure A3). (**C**) Immunoblot with densitometry (total pixels) underneath quantifies HGF’s receptor, Met (β subunit, 145 kDa), among cell lines. For comparison, HGF-stimulated cell migration is below the bar graph as times (X) background cell migration and lacks notable correlation. The immunoblot’s gel is in Appendix A (Figure A4A). (**D**) MEM as media in the bottom wells, with and without FBS, outperformed Dulbecco’s PBS for U87 cell migration with “Migration” media in all top wells. Migration was 1.7X, 1.1X, and 1.4X greater (significantly, *p* < 0.05) using MEM compared to PBS for 0, 0.1, and 1% FBS in the bottom wells, respectively. MEM’s greater potassium content (5.3 mM) in the bottom wells than in “Migration” media (4.5 mM K^+^; see text) in the top wells putatively provided a chemoattractant K^+^ gradient. *p* =0.0144 in MEM versus Dulbecco’s PBS with 0% FBS, and with 0.1 and 1% FBS it was also present, *p* = 0.0136 and 0.000422, respectively. The scanned image of the assay is in Appendix A (Figure A4B). (**E**) Chemotaxis to HGF and FBS outperformed chemokinesis and FBS enhanced HGF’s effects on U87 cells in MEM (top and bottom wells). Chemotaxis was consistently greater than chemokinesis in paired comparisons designated by brackets under the x-axis with significance, *p* = 0.012, for row 8 versus row 9. The assay image is in Appendix A (Figure A4C). Averages of rows’ densitometry units indicated by bar heights with 95% confidence intervals. Variable staining of background migration and assay-to-assay variation for each cell line in Appendix A are routine. Each circle of migrated cells is 3 mm in diameter.

Earlier studies of U87 and LN229 glioblastoma cell lines and a clinical specimen’s cells reported chemoattraction to HGF and serum [43,62,134,135,136]. Testing on additional glioblastoma cell lines for migration to HGF and FBS, comparing levels of HGF’s receptor, Met, among them, comparing MEM (provided a positive K+ gradient) versus Dulbecco’s PBS (no K+ gradient), and more extensive testing of chemokinetic effects, are reported here. Boyden assays (48 wells), three per cell line, for two rat astrocytoma cell lines (C6 and F98) and five human glioblastoma cell lines (T98G, LN18, U87, LN229, and U373), showed cell migration to HGF alone as a chemoattractant (row 2), HGF and FBS together as chemoattractants (rows 3–9), and to FBS alone as a chemoattractant in a dose curve (rows 10–12), compared to background (unstimulated) cell migration (row 1). The concentrations of chemoattractants are indicated in the left columns. With the same amount of HGF present, adding increased amounts of FBS increased cell migration in seven of eight cell lines (Figure 2A).

**Figure A2:**
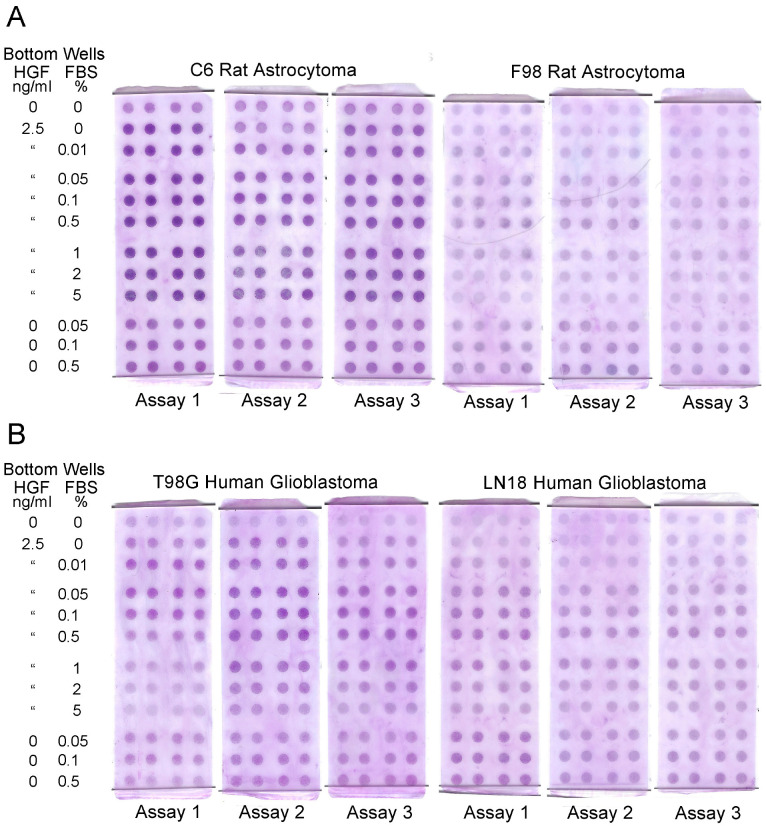
All three migration assays for each cell line (C6, F98, T98G, and LN18) in studies of HGF and FBS chemoattraction. (**A**) Three migration assays for C6 and F98 cell line. (**B**) Three migration assays for T98G and LN18 cell line. One assay per cell line is shown in Figure 2A. Portions of assays that were digitized and analyzed are shown in Figure 2B. Text in Section 3.2. and the Figure 2 legend provides additional information. Each circle of stained migrated cells is 3 mm in diameter.

**Figure A3:**
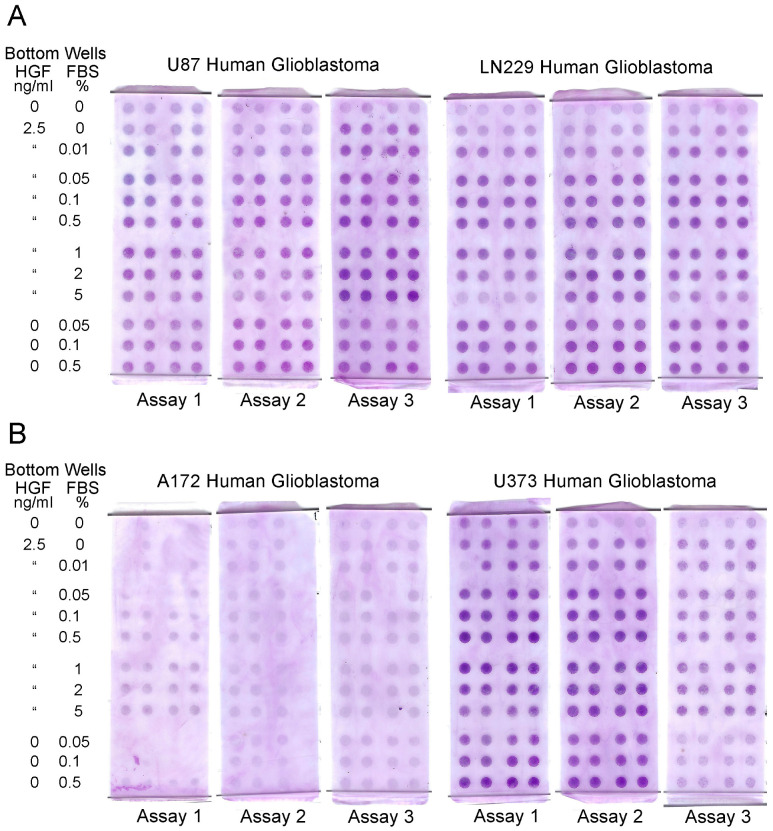
All three migration assays for each cell line (U87, LN229, A172, and U373) in studies of HGF and FBS chemoattraction. (**A**) Three migration assays for U87 and LN229 cell line. (**B**) Three migration assays for A172 and U373 cell line. One assay per cell line is shown in Figure 2A. Portions of assays that were digitized and analyzed are shown in Figure 2B. Text in Section 3.2. and the Figure 2 legend provides additional information. Each circle of stained migrated cells is 3 mm in diameter.

**Figure A4:**
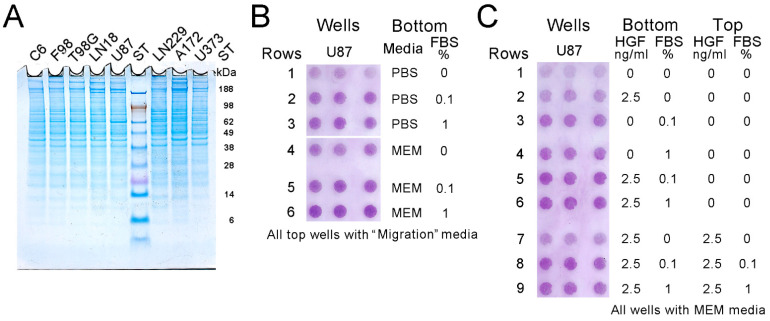
Additional images for Figure 2. (**A**) Gel image for the immunoblot in Figure 2C, showing equal loading of protein in each lane for all 8 cell lines. (**B**) Scanned image of the U87 migration assay shown in Figure 2D. Dulbecco’s PBS and MEM were tested as media in the lower wells for migration without and with FBS (2 concentrations) present. The image of the filter removed (white horizontal line) an unrelated portion of the assay that tested a different batch of FBS for suitability in future migration assays. (**C**) Scanned image of the U87 migration assay shown in Figure 2E. Chemoattraction of HGF and 2 concentrations of FBS and chemokinesis of HGF, with and without 2 concentrations of FBS, were analyzed in U87 cells. Text in Section 3.2. and the Figure 2 legend provides additional information. Each circle of stained, migrated cells is 3 mm in diameter.

Images of FBS alone as the chemoattractant in a dose curve are shown as migrated cell pellets with densitometry results in bar charts. All cell lines, except A172, showed significant migration to FBS alone. The U87 and U373 results showed impressive stepwise increases in chemotactic migration, with *p* values < 0.0005 and 0.000005, respectively. U373 chemotaxis to 0.5% FBS, 3.8 times background migration, was highest for all cell lines. A172 cells showed a trend for increased migration to 0.5% FBS; *p* = 0.0638. Some cell lines showed flattening of responses as levels of FBS increased (Figure 2B). All assays (three per cell line) are shown in Appendix A for C6, F98, T98G, and LN18 (Figure A2) and U87, LN229, A172, and U373 (Figure A3). Cell migration specifically for HGF in each cell line is stated as a multiple (X) of background cell migration (second row of assays’ cell pellet densitometry results divided by corresponding first row of background cell migration); see bottom of Figure 2C. HGF cell migration from highest to lowest for all three assays of each cell line was T98G (2.11X), U373 (1.93X), LN229 (1.52X), U87 (1.49X), C6 (1.33X), A172 (1.30X), LN18 (1.15X), and F98 (1.13X). Twelve of 92 data points showing the migrated HGF cell pellets’ densitometry being slightly less than 1X background cell migration were rounded to 1X. A few replicates did not show visible cell pellets. The immunoblot shows reactivity for HGF’s receptor, Met (β subunit, 145 kDa). Met reactivity with U87 and LN229 [134] is shown here with six other cell lines; there is no correlation with the cell motility results shown below (Figure 2C). The stained gel image showing equal protein loading for cell line lysates in the immunoblot is in Appendix A (Figure A4A).

Another assay used to evaluate MEM for migration studies included gradients of K^+^ and Cl^−^ in migration assays of U87 cells. Both ions are redistributed with volume changes during cell migration. In the absence of FBS, Dulbecco’s PBS in the bottom wells provided K^+^ as 2.7 mM KCl + 1.8 mM KH_2_PO_4_ (4.5 mM K^+^) and Cl^−^ as 2.7 KCl + 137.9 mM NaCl (140.6 mM Cl^−^). MEM in the lower wells provided K^+^ as 5.3 mM KCl (5.3 mM K^+^) and Cl^−^ as 5.3 mM KCl + 1.8 mM CaCl_2_-2H_2_O (8.9 mM Cl^−^). See Methods for a minor issue with KCl molarity in MEM. For all upper wells without FBS, “Migration” media (includes Dulbecco’s PBS) provided K^+^ as 2.7 mM KCl + 1.8 mM KH_2_PO_4_ (4.5 mM K^+^) and Cl^−^ as 2.7 KCl + 137.9 mM NaCl + 0.21 mM MgCl_2_ + 0.243 mM CaCl_2_ (141.5 mM Cl^−^). The filter pores between upper and lower wells were 8 × 7 μm (with cells obscuring openings). Potassium did not generate any gradient for cells migrating from upper to lower wells when Dulbecco’s PBS was used in the bottom wells and there was almost no gradient for chloride. However, when MEM was used in the lower wells, a reverse gradient for Cl^−^ was generated. With MEM in the lower wells, there was a positive potassium gradient for migrating cells (5.3 mM in lower versus 4.5 mM in upper wells). The positive gradient for K^+^ may have contributed to significantly increased migration, *p* = 0.0144, without FBS present. MEM as a medium in the lower wells was also associated with significantly increased cell migration when 0.1 and 1% FBS was present, *p* = 0.0136 and *p* = 0.000442, respectively, compared to using Dulbecco’s PBS in the lower wells (Figure 2D). MEM contains 5.6 mM dextrose, 1 mM pyruvate, and 2 mM l-glutamine, whereas the “Migration” media contained 5.551 mM glucose, 5 mM pyruvate, and 0.1% bovine serum albumin as ingredients not already mentioned. Therefore, MEM as a source of fuel in the bottom wells was probably not a significant differentiating factor in the results. MEM also contains 17.856 mM sodium bicarbonate, 0.8 mM magnesium sulfate (more Mg^2+^ than “Migration” media (0.21 mM MgCl_2_)), less sodium than Dulbecco’s PBS and “Migration” media, and amino acids, vitamins, etc. An assay image is shown in Appendix A (Figure A4B). These results support the role of K^+^ in enhancing cancer cell migration.

Earlier studies had noted chemokinesis with HGF and 0.1% FBS in U87 cell assays [136] and chemokinesis with 5% FBS alone in LN229 cell invasion assays [135]. The dose curve for FBS includes additional data points, as shown in Figure 2E. The chemoattractant strength of HGF alone (row 2), and with 0.1% and 1% FBS (rows 5 and 8, respectively) was enhanced by both FBS concentrations in U87 cells. The chemokinetic strength of HGF alone (row 3), with 0.1% and 1% FBS (rows 6 and 9, respectively), was also enhanced by FBS. Chemotaxis and chemokinesis with 0, 0.1, and 1% FBS demonstrated strong enhancements provided by FBS versus HGF alone in either type of U87 cell migration. The combination of HGF, 2.5 ng/mL, and 1% FBS for chemoattraction significantly, *p* = 0.012, outperformed their chemokinetic effects on cell migration (Figure 2E). A scanned assay image is shown in Appendix A (Figure A4C).

Variable background migration (Figure 2A,B) and assay-to-assay variation for each cell line, as shown in files in Appendix A (Figure A2 and Figure A3), are routine for cell migration assays.

### 3.3. KCN Genes Differentially Expressed in 70 Studies (E1–E70)

#### 3.3.1. General Features of Detected Differentially Expressed KCN Genes

Availability of K^+^ for glioblastoma cell fitness and migration led to consideration of KCN genes being altered in cancer cells. Genomic landscapes, i.e., heat maps (46 studies), Venn diagrams, volcano plots, tables, etc., in the 70 cancer studies included one or more of 53 KCN differentially expressed genes (DEGs) with co-expressed non-KCN DEGs. Selection criteria (mostly *p* < 0.05 differences, chance of false discovery, etc.) were set by the authors, who incidentally detected KCN DEGs among groups of DEGs, as listed in Appendix B (Table A1, sample view; entire list is in Supplementary Materials: Table S1: FullTblB1DEGalphaAZ). Parameters were applied for a particular type of malignancy, without or with perturbations, such as tumor treatment, transcription factor, over-expression of oncogene(s), survival, disease progression, primary versus metastasis, recurrence, etc. The 53 KCN DEGs are from 17 chromosomes (1–3, 5–12, 17–21, and X). Many encode membrane pore subunits with selectivity for K^+^ and some provide modulatory/regulatory effects. Subfamilies, Kv (voltage dependent, *KCNA*, *KCNQ*, *KCNH*, etc.), Kir (inwardly rectifying, *KCNJ*), K2P (two pore domain, *KCNK)*, and KCa (calcium dependent, *KCNM*, *KCNN*, etc.) were found in 32 (46%), 10 (14%), 14 (20%), and 29 (41%), respectively, of the E1–E70 studies (some with multiple subfamily members).

#### 3.3.2. Proton (H^+^) Sensitivity of Detected KCN DEGs

**Figure 3:**
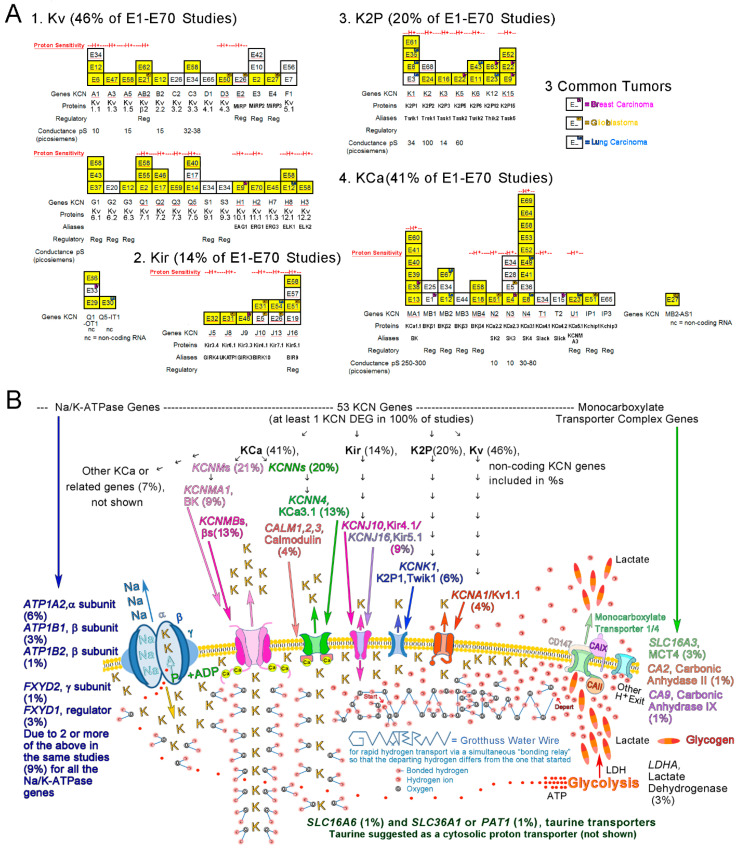
KCN DEGs and related DEGs detected in E1–E70 studies. (**A**) The 53 KCN DEGs are listed horizontally, with specific studies listed vertically showing their frequency in 70 studies. The 3 most frequent tumors (breast, glioblastoma, and lung) have color-coded superscripts. The 33 (62.3%) KCN DEGs that encode H^+^-sensitive proteins are labeled with red H^+^ above each column and have their names underlined in red. A total of 52 (74.3%) of 70 studies contain at least one KCN DEG encoding an H^+^-sensitive protein. Yellow shading indicates the study was in 1 or more of 8 categories (see Figure 4A). Non-coding KCN DEGs are in separate histograms. (**B**) A stochastic view of K^+^-related dynamics is based on collective incidences of KCN and related DEGs in E1–E70. The most frequent members of KCN subfamilies with pertinent non-KCN proteins shown reflect literature representations [140,141,142,143,144,145,146,147]. Relatively few studies in E1–E70 detected DEGs encoding Na/K-ATPases (9% for 5 genes), the monocarboxylate transporter complex (5% for 3 genes), lactate dehydrogenases (3% for 1 gene), taurine transporters (2% for 2 genes), and 0% for those not listed. Therefore, it is proposed that abnormal K^+^ flux via KCN DEGs (assorted combinations) initiates pH changes by releasing protons for transport (putatively Grotthuss-type transport) to exit sites using relatively intact supporting systems. Specifically, abnormal accumulation of K^+^ at inner-membrane surfaces that forces exchange with bound H^+^ is proposed to initiate pH reversal. Also, movement of K^+^-7H_2_O (2 H_2_O per K^+^ in “cross-section”) may bring water to inner-membrane regions to enhance Grotthuss conditions in cancer cells. Putative K^+^-related changes in cancer cells maintaining relatively normal expression of pertinent non-KCN genes, as seen in most of the 70 KCN DEG studies, would be consistent with pH reversal, as shown (**B**). E1–E70 citations are listed in Section 3.3.6.

In keeping with K^+^ and H^+^ being monovalent cation relatives, the H^+^ sensitivity of KCN DEG-encoded proteins is relevant. At least 33 (62.3%) of the 53 KCN DEGs encode gene products that are H^+^-sensitive (130 publications in Appendix B, Table A2). Fifty-two (74.8%) of the 70 studies contained at least one KCN DEG identified as H^+^-sensitive. One, two, three, and six H^+^-sensitive KCN DEG genes were found in 36, 14, 1, and 1 of the studies, respectively. Genes for H^+^-sensitive proteins in Kv, Kir, K2P, and KCa subfamilies were found as DEGs in 17 (24%), 9 (13%), 13 (19%), and 21 (30%) of the 70 studies, respectively. The most frequently detected DEGs, *KCNN4* and *KCNMA1*, present in nine and six studies, respectively, encode H^+^-sensitive proteins (red H^+^ above columns) and are also Ca^2+^-sensitive (Figure 3A).

#### 3.3.3. Coding and Multiplicity of KCN DEGs and Non-KCN DEGs

Four of the 70 studies identified only non-coding KCN DEGs: *KCNQ1-OT1* (E29, E33, and E66) and *KCNQ5-IT1* (E30). Study E27 identified *KCNMB2-AS1* and *KCNE4* as DEGs. Multiple (two to nine) KCN DEGs were found in 18 (27.3%) of 66 studies with protein-coding KCN DEGs. Thirty-eight (71.7%) of 53 KCN DEGs were detected with other coding KCN DEGs in 66 of the studies. There were 102 instances of 53 KCN DEGs in 3239 DEG instances (2490 without repeats). The average numbers of KCN DEGs and all DEGs per study were 1.53 +/− 0.33 (95% CI) and 45.91+/− 7.92 (95% CI), respectively. The correlation coefficient for the number of KCN DEGs detected and size of the DEG groups was 0.379. Fifty-two (78.8%) of 66 studies with coding KCN DEGs detected at least one H^+^-sensitive KCN DEG (Figure 3A).

#### 3.3.4. Malignancies in E1–E70

**Figure A5:**
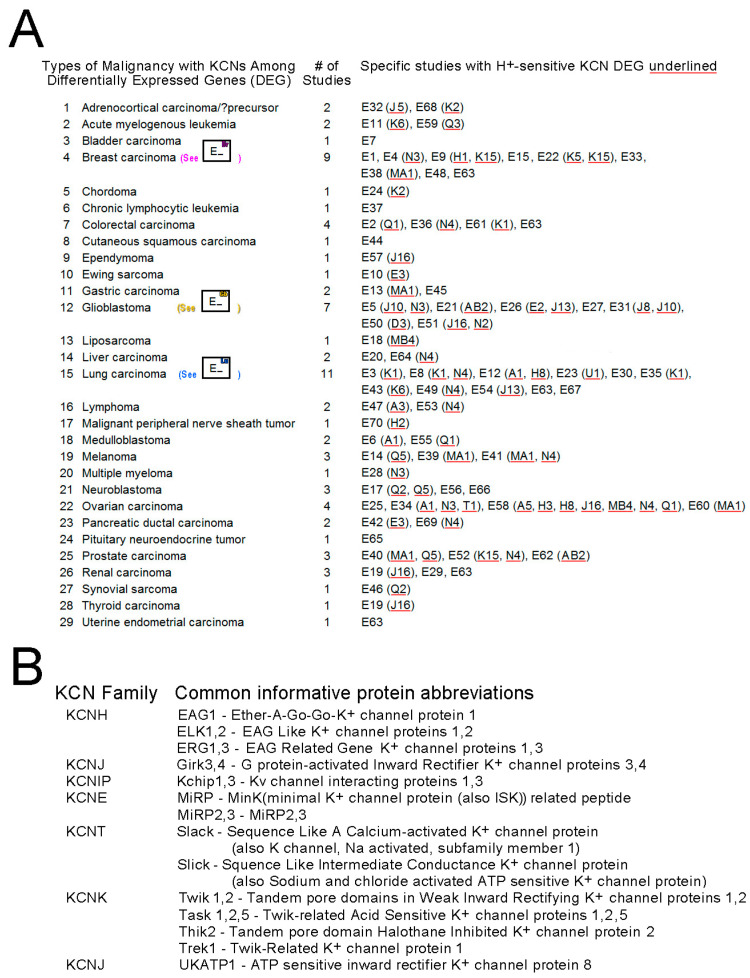
Types of malignancies studied in E1–E70 and informative protein abbreviations for proteins in the KCN family. (**A**) Specific studies for 29 malignancies with their detected KCN DEGs. Red underlining indicates KCN DEGs that encode H^+^-sensitive proteins. Boxes that correspond with studies of glioblastoma, breast cancer, and lung cancer are color-coded: yellow, pink, and blue, respectively. These graphics are also used in the histograms shown in Figure 3 and Figure 4. (**B**) Some common protein abbreviations for KCN family members in Figure 3, indicating functional information not explained in the text.

The three most frequent malignancies in E1–E70 were lung cancer, breast cancer, and glioblastoma, with detected genes encoding H^+^-sensitive products **bolded** as follows. Lung cancer studies included E3 (***KCNK1***), E8 (***KCNK1***, ***KCNN4***), E12 (***KCNH8***, *KCNMB2*), E23 (***KCNU1***), E30 (*KCNQ5-IT1*), E35 (***KCNK1***), E43 (***KCNK6***), E49 (***KCNN4***), 2), E54 (***KCNJ13***), E63 (*KCNK12*), and E67 (*KCNMB2*). Breast cancer studies included E1 (*KCNMB1*), E4 (***KCNN3***), E9 (***KCNH1***, ***KCNK15***), E15 (*KCNT2*), E22 (***KCNK5***, ***KCNK15***), E33 (*KCNQ1-OT1*), E38 (***KCNMA1***), E48 (*KCNJ9*), and E63 (*KCNK12*). Glioblastoma studies included E5 (***KCNJ10***, ***KCNN3***), E21 (***KCNAB2***), E26 (***KCNE2***, ***KCNJ13***), E27 (KCNE4, KCNMB2-AS1), E31 (***KCNJ10***), E50 (***KCND3***), and E51 (KCNIP1, ***KCNJ16***, ***KCNN2***). H^+^-sensitive coding KCN DEGs were in 86%, 73%, and 44% of 7 glioblastoma, 11 lung cancer, and 9 breast cancer studies, respectively. All three melanoma studies detected H^+^-sensitive KCN DEGs. Numerous types of malignancy detected H^+^-sensitive KCN DEGs; see Appendix B (Figure A5A). Informative KCN protein abbreviations are listed in Figure A5B. E1–E70 citations for E1–E70 are given in Section 3.3.6.

#### 3.3.5. Stochastic View of K^+^-Related Dynamics in E1–E70

A stochastic view (percentages) of K^+^-related gene expression in cancer cells is presented based on incidences of KCN and related non-KCN DEGs. Images of the most frequent members of the KCN subfamilies with pertinent non-KCN proteins are consistent with literature representations [140,141,142,143,144,145,146,147]. The relatively small number of 70 studies with differential expressions detected for genes, shown in Figure 3B, that encode supporting systems, i.e., Na/K-ATPase (9% for five genes), monocarboxylate transporter complex (5% for three genes), lactate dehydrogenases (3% for one gene), taurine transporters (2% for two genes), and 0% for genes encoding functional analogues not listed, support the implication of abnormal K^+^ flux in cancers with KCN DEGs forcing K^+^/H^+^ exchange to initiate pH changes. Rapid cytosolic H^+^ transport (putatively Grotthuss water wires) to exit sites for proton release with lactate completes the pH reversal outcome. Pertinent DEGs lacking in E1–E70 include glycogen synthases that could mediate anomalous storage of lactate without extrusion, since its storage as glycogen occurs in humans, etc. [148,149,150,151]. Accumulations of K^+^ may happen due to genetic loss or downregulation of KCN proteins mediating outward flux or increased inward flux due to gains or losses in copy number and/or expression of KCN DEGs in E1–E70. Movement of K^+^-7H_2_O within cells may bring water to inner membranes, possibly promoting Grotthuss conditions. Putative K^+^-related changes in cells that maintain relatively normal expression levels of pertinent non-KCN genes, as seen in most of the KCN DEG studies, support the processes shown in Figure 3B.

#### 3.3.6. Categories Among Studies with E1–E70 Citations

**Figure 4:**
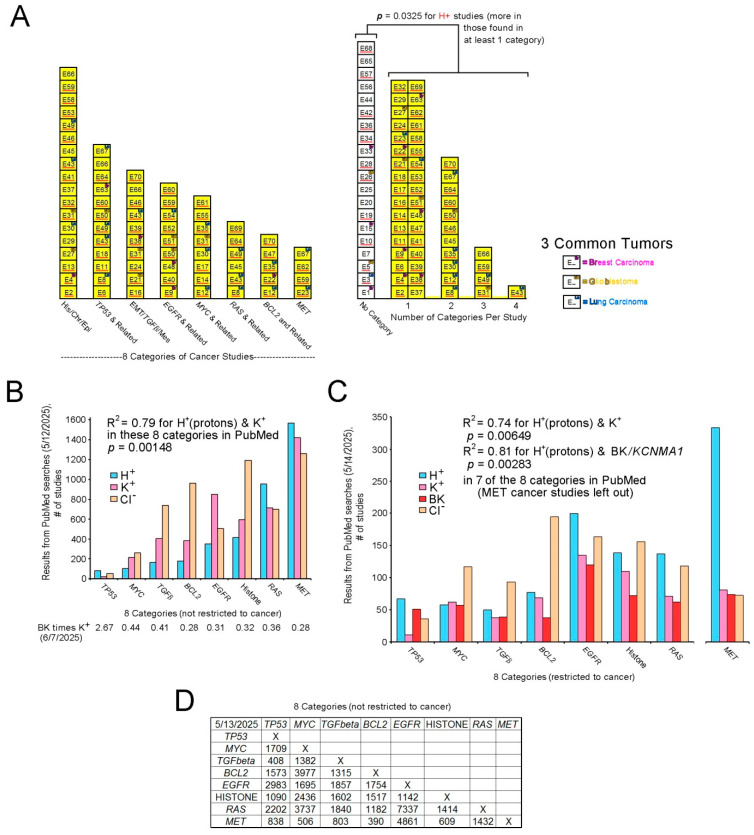
Oncogenes and DNA modifiers may play a role in cancer studies via KCN DEGs encoding H^+^-sensitive proteins. (**A**) KCN DEGs that encode H^+^-sensitive proteins are highlighted with red underlining of study names. Histograms of 8 categories for cancer studies among E1–E70 are shown on the left (yellow boxes). The right histogram also includes 20 studies in “no category” (white boxes). Comparison of studies in “no category” versus those in at least one category shown in the left histogram found a significant difference in the number of studies detecting KCN DEGs encoding H^+^-sensitive proteins using Fisher’s exact test, *p* = 0.0325, indicating a higher likelihood for studies included in at least one category. (**B**–**D**) Word searches of PubMed for numbers of oncogene and histone publications related to H^+^, K^+^, BK(*KCNMA1*), and Cl^−^ (contrasting ion) were performed to further evaluate categories in E1–E70. (**B**) On 12 May 2025, searches for each category of “TP53”, “MYC”, “TGFbeta”, “BCL2”, “EGFR”, “histone”, “RAS”, and “MET” were performed separately for “proton”, “potassium”, and “chloride”. For all 8 categories, the numbers of citations for “proton”, “potassium”, and “chloride” were 1565, 1418, and 1261, respectively, and Pearson’s product-moment correlations for “proton” with “potassium” and “proton” with “chloride” were R = 0.891 (*p* = 0.00148) and 0.599 (*p* = 0.0584), respectively. Pearson’s product-moment correlation for “potassium” and “chloride” was 0.689 (*p* = 0.0295). R^2^ for “proton” with “potassium” was 0.79. “BK” and “potassium” searches on 7 June 2025revealed “BK” as a potential large component of “potassium” citations, approximately one-third for 7 categories. (**C**) When searches also included “cancer” as a third word, the numbers of citations for “proton”, “potassium”, “BK”, and “chloride” on 14 May 2025, were 1061, 577, 513 (88.9% of “potassium” search results), and 952, respectively. When MET was left out for better “proton” correlations, the numbers of citations for “proton”, “potassium”, “BK”, and “chloride” on 14 May 2025, were 728, 496, 439 (88.5% of “potassium” search results), and 879, respectively, and Pearson’s product-moment correlations for “proton” with “potassium” and “chloride” were R = 0.860 (*p* = 0.00649) and 0.462 (*p* = 0.148), respectively, across the 7 remaining categories. Pearson’s product-moment correlation for “proton” with “BK” was R = 0.901 (*p* = 0.00283) across 7 categories. R^2^ for “proton” with “BK” was 0.81. R^2^ for “proton” with “potassium” was 0.74. Results among the ions for correlation, with numbers of “Met” citations included, differed markedly in “cancer” searches compared to search results not restricted to “cancer” for unknown reasons. (**D**) Numbers of citations shared among categories of 7 oncogenes and histone are shown. E1–E70 citations are listed in Section 3.3.6.

Eight categories describe 50 of the 70 studies (Figure 4A). These were designated by the authors and accepted if there were relevant DEGs in any of the E1–E70 studies. Some studies were in multiple categories and 20 studies were in no category.

1. Of 18 studies in the first category of histone/chromatin/epigenetic (His/Chr/Epi), 10 had category-specific genes described by the authors, including E2 (*N6AMT1*, previously *KMT9*), E13 (*MSH2*, *BET*), E32 (*H3K27me3*, *EZH2*), E41 (*HDAC8*), E46 (*KDM2B*), E49 (*KMT2D*), and E53 (*H3K27*). Also, E27 and E29 focused on chromatin access and imprinting, respectively. A total of 21 imprinted DEGs (*AXL*, *DLK1*, *GDAP1L1*, *GNG7*, *GRB10*, *IGF2*, *KCNQ1*, *KCNQOT1*, *LRRTM1*, *MEG3*, *NDN*, *NNAT*, *NTM*, *PAX8*, *PEG3*, *PEG10*, *PHLDA2*, *PLAGL1*, *RHOBTB3*, *RTL1*, and *TFPI2*) accounted for 32 instances of imprinted genes among 3239 DEG instances (repeats included) for a rate of 0.99% in E1–E70. Detection of 0.99% imprinted genes is expected from the genome (approximately 200, i.e., <1% of all genes) [152]. Therefore, imprinted genes were not used as an inclusion criterion. Eight additional studies detected His/Chr/Epi-related DEGs as follows: E4 (*DNMT3B*), E30 (*KDMA-AS1*), E31 (*H2A.X*, previously *H2AFX*), E37 (*H2AC18*), E43 (*H2AC18*, *H2BC5*, *H2BC12*), E45 (*NUPR1*), E58 (*DNMT3B*), and E66 (*BDR4*, *H1-4* (previously *HIST1HIE*)). The His/Chr/Epi category includes the following studies (with citations): E2 [153], E4 [154], E13 [155], E27 [156], E29 [157], E30 [158], E31 [159], E32 [160], E37 [161], E41 [162], E43 [163], E45 [164], E46 [165], E49 [166], E53 [167], E58 [168], E59 [169], and E66 [170] (Figure 4A, left histogram).

2. In the second category there were nine studies on *TP53* according to the authors, in E6, E8, E11, E49, E49, E60, E63, E64, and E67. Additionally, three studies found *TP53* and related genes as DEGs as follows: E18 (*MDM2*), E43 (*TP53INP1A*), and E50 (*TP53*). The twelve studies in this category are E6 [171], E8 [172], E11 [173], E18 [174], E43 [163], E49 [166], E50 [175], E60 [176], E63 [177], E64 [178], E66 [170], and E67 [179] (Figure 4A, left histogram).

3. In the third category there were seven studies on epithelial mesenchymal transition (EMT)/*TGFb*/mesenchymal tumor (EMT/*TGFb*/Mes) according to the authors, with specific genes *CDH1*,*2*, *VIM*, and *TGFb1*,*2*,*3* in studies as follows: E16 (*CDH1*,*2*, *TGFb1*, *VIM*), E24 (*TGFb1*), E31, E38, E39, E43 (*CDH2*), and E46. Three additional studies also found these genes: E21 (*TGFb1*), E66 (*VIM*), and E70 (*CDH2*, *TGFb2*, *TGFb3*). The ten studies in this category are as follows: E16 [180], E21 [181], E24 [182], E31 [159], E38 [183], E39 [184], E43 [163], E46 [165], E66 [170], and E70 [185] (Figure 4A, left histogram).

4. In the fourth category there were five studies on *EGFR* and related genes according to the authors, with corresponding DEGs in the studies as listed: E9, E48, E50 (*EGFR*, *ERBB2*), E51 (*EGFR*), and E52. Additionally, four studies found related DEGs as follows: E40 (*NRG1*), E54 (*NRG2*), E59 (*NRG1*), and E60 (*ERBB4*). The nine studies in this category are E9 [186], E40 [187], E48 [188], E50 [175], E51 [189], E52 [190], E54 [191], E59 [169], and E60 [176] (Figure 4A, left histogram).

5. In the fifth category there were five studies on *MYC* according to the authors, with *MYC* as a DEG in two of these studies: E14 (*MYC*), E30, E31, E55, and E61 (*MYC*). Additionally, three studies had *MYC* or related genes: E12 (*MYCL*), E17 (*MYCN*), and E35 (*MYC*). The eight studies in this category are E12 [192], E14 [193], E17 [194], E30 [158], E31 [159], E35 [195], E55 [196], and E61 [197] (Figure 4A, left histogram).

6. The sixth category was comprised of six studies on *RAS* per the authors’ interest, with *KRAS* found as a DEG in two of the studies: E8, E43, E45, E49, E64 (*KRAS*), and E69 (*KRAS*). The six studies in this category are E8 [172], E43 [163], E45 [164], E49 [166], E64 [178], and E69 [198] (Figure 4A, left histogram).

7. In the seventh category the authors focused on *BCL2* in E47 but without listing it as a DEG. However, other studies identified *BCL2* as a DEG, and one also identified *BCL2L1*, as listed: E12 (*BCL2*), E22 (*BCL2*), E35 (*BCL2*), and E70 (*BCL2*, *BCL2L1*). The five studies in this category are E12 [192], E22 [199], E35 [195], E47 [200], and E70 [185] (Figure 4A, left histogram).

8. In the eighth category, the authors of one study focused on *MET* and found it as a DEG, E23 (*MET*). Three studies also identified *MET* as a DEG, E59 (*MET*), E62 (*MET*), and E67 (*MET*). The four studies in this category are E23 [201], E59 [169], E62 [202], and E67 [179] (Figure 4A, left histogram).

All the studies in the eight categories are shaded yellow in Figure 4.

9. No category. Twenty studies were not in any of the eight categories: E1 [203], E3 [204], E5 [205], E7 [206], E10 [207], E15 [208], E19 [209], E20 [210], E25 [211], E26 [212], E28 [213], E33 [214], E34 [215], E36 [216], E42 [217], E44 [218], E56 [219], E57 [220], E65 [221], and E68 [222] (Figure 4A, right histogram, no yellow shading).

#### 3.3.7. H^+^ Sensitivity in E1–E70 Studies Within Eight Categories

The histogram in Figure 4 (right side) shows studies collectively in the eight categories versus those not in any category, with studies that detected H^+^-sensitive KCN DEGs underlined with red. The difference in detection of H^+^-sensitive KCN DEGs was significant between the two groups: *p* = 0.0325, Fisher’s exact test for count data. All eight categories were among those shared in the studies (Figure 4A**,** right histogram, yellow shading). The citations for E1–E70 are listed in Section 3.3.6.

#### 3.3.8. PubMed Citations for “Proton”, “Potassium”, Etc., in Categories

Word searches of PubMed for oncogene and histone publications related to those for H^+^, K^+^, BK (*KCNMA1*), and Cl^−^ (contrasting ion), without and with “cancer”, were performed to further evaluate the categories. On 12 May 2025, searches for each category of TP53, MYC, TGFbeta, BCL2, EGFR, histone, RAS, and MET were performed separately for “proton”, “potassium”, and “chloride”. For all eight categories, the total citations for “proton”, “potassium” and “chloride” were 1565, 1418, and 1261, respectively, and the Pearson’s product-moment correlations for “proton” with “potassium” and “chloride” were R = 0.891 (*p* = 0.00148) and 0.599 (*p* = 0.0584), respectively. Pearson’s product-moment correlation for “potassium” with “chloride” was 0.689 (*p* = 0.0295). The R^2^ for proton with potassium was 0.79. Later searches on June 7 2025for “BK” and “potassium” revealed “BK” citations were about one-third of those for “potassium” in seven categories (TP53 was much greater); they are listed below the x-axis in Figure 4B. When searches also included “cancer” as a third word, the numbers of citations for “proton”, “potassium”, “BK”, and “chloride” on 14 May 2025, were 1061, 577, 513, and 952, respectively. However, when MET was left out, the numbers of citations for “proton”, “potassium”, “BK”, and “chloride” on 14 May 2025, were 728, 496, 439, and 879, respectively, and the Pearson’s product-moment correlations for “proton” with “potassium” and “chloride” were improved, R = 0.860 (*p* = 0.00649) and 0.462 (*p* = 0.4623), respectively, using only seven categories.. The R^2^ for “proton” with “potassium” for seven categories after MET removal was 0.74. The Pearson’s product-moment correlation across the seven categories for “proton” with “BK” was R = 0.901 (*p* = 0.00283). The R^2^ for the correlation of “proton” with “BK” across the seven categories was 0.81. It is unknown why citations in the MET category no longer resulted in the correlation seen in previous searches without “cancer” for “proton” and “potassium”, but the possibilities include unpublished negative studies. The answer is beyond the scope of this study. However, see Figure 2C for negative results for Met in this study. Also, compared to searches without “cancer”, as shown in Figure 4B, “BK” citations appeared to constitute an even larger proportion of the “potassium” searches when “cancer” was included (Figure 4C). Numerous citations were shared pairwise in two-word searches (not restricted to cancer) among the eight categories of seven oncogenes and histone (Figure 4D). Thus, studies involving the eight categories may often describe them collectively. Citation searches for KCN proteins other than BK yielded relatively low numbers.

#### 3.3.9. Non-KCN DEG Repeats in E1–E70

**Figure 5:**
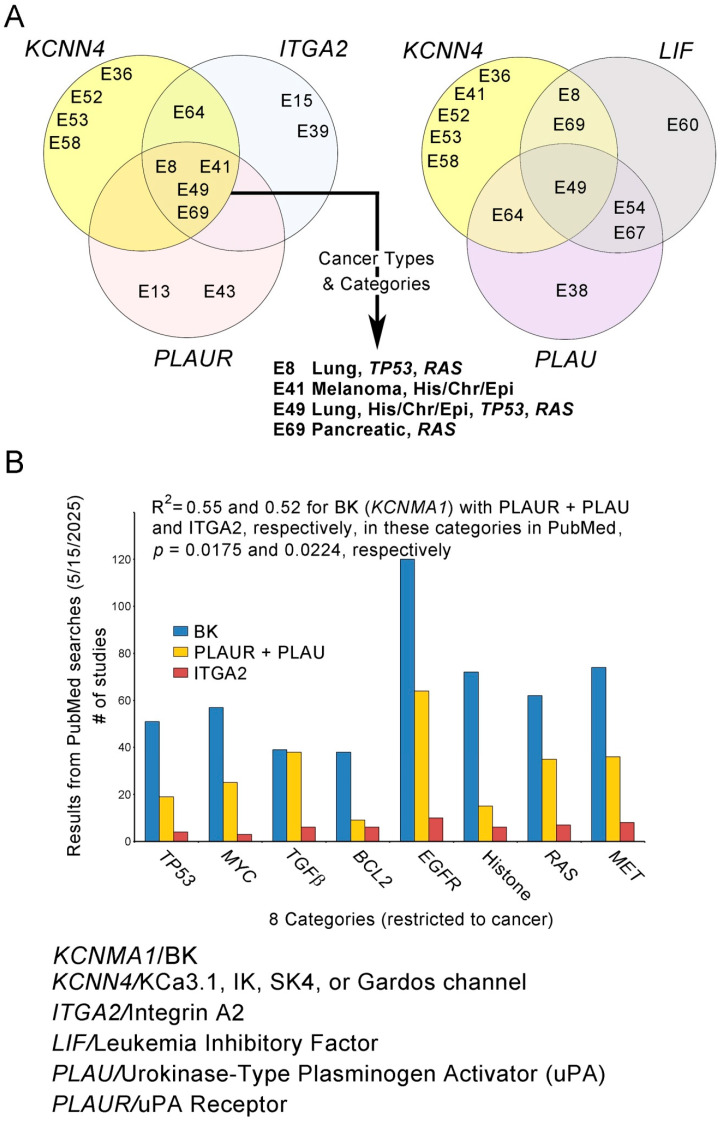
Repeat non-KCN DEGs were often found with *KCNN4* and some with *KCNMA1*. (**A**) Among non-KCN DEGs, *ITGA2*/Integrin Alpha-2 was most frequent (7 studies). Six also included H^+^-sensitive coding KCN DEGs (bolded): **E8** (***KCNK1*, *-N4***)**,** E15, **E39** (***KCNMA1***), **E41** (***KCNMA1***, ***-N4***), **E49** (***KCNN4***), **E64** (***KCNN4***), and **E69** (***KCNN4***). *KCNN4* was the most frequent, 9 times in total and was also in 5 of 7 studies with *ITGA2*. Studies of co-expressed *KCNN4*, *ITGA2*, and *PLAUR*, E8 [172], E41 [162], E49 [166], and E69 [198], included 2 lung cancers, 1 melanoma, and 1 pancreatic cancer, in categories of *His/Chr/Epi*, *TP53*, and *RAS* (top left Venn diagram). E49 detected *KCNN4*, *LIF*, and *PLAU* together (top right Venn diagram). E41 [162] detected *KCNMA1*, *KCNN4, ITGA2*, and *PLAUR* (not shown). (**B**) PubMed word searches for shared citations among “BK” (*KCNMA1*) and “PLAUR” + “PLAU” and “ITGA2” found correlations, R^2^ = 0.55, *p* = 0.0175 and R^2^ = 0.52, *p* = 0.0224, respectively, across 8 categories. # = number.

Non-KCN DEGs found in multiple (five to seven) studies were often detected with *KCNN4* and some with *KCNMA1*. Among non-KCN repeat DEGs, *ITGA2*/Integrin Alpha-2 was most frequent (seven studies). Six also included H^+^-sensitive coding KCN DEGs (bolded): **E8** (***KCNK1*, *-N4***)**,** E15, **E39** (***KCNMA1***), **E41** (***KCNMA1***, ***-N4***), **E49** (***KCNN4***), **E64** (***KCNN4***), and **E69** (***KCNN4***). *KCNN4* was the most frequently found DEG, nine times in total and in five of seven studies with *ITGA2*. Studies of co-expressed *KCNN4*, *ITGA2*, and *PLAUR*, E8 [172], E41 [162], E49 [166], and E69 [198], included two lung cancers, one melanoma, and one pancreatic cancer, in the categories of *His/Chr/Epi*, *TP53*, and *RAS* (Figure 5A**,** left Venn diagram). *HMGA2*/High Mobility Group AT-hook 2, *LIF*/Leukemia Inhibitory Factor, and *PLAUR*/Plasminogen Activator, Urokinase Receptor (UPAR) were DEGs found in six studies each. These studies also frequently detected *KCNN4* as a DEG.

One study detected *KCNN4*, *LIF*, and *PLAU* (Figure 5A, right Venn diagram). Two studies detected *KCNN4*, *ITGA2*, and *PLAU* (not shown). The *PLAUR* and *PLAU* DEGs were found together in 1 study among 10 studies that detected either of them. E41 [162] detected *KCNMA1*, *KCNN4, ITGA2*, and *PLAUR* (not shown).

Searches of PubMed for shared citations for “BK” with “PLAUR” + “PLAU” (combined separate searches) and “ITGA2” in three-word searches including “cancer” found significant Pearson’s product-moment correlations of R = 0.742 (*p* = 0.0175) and 0.718 (*p* = 0.0224), respectively, and R^2^ = 0.55 and 0.52, respectively, across the eight categories (Figure 4B).

Also, some of the non-KCN DEGs found in E1–E70 are shared with studies of genes encoding circulating proteins associated with cancer risk. The shared DEGs from the E1–E70 studies, with their frequencies, are *FGFR3* (5X), *GDF15* (4X), *IGFBP3* (5X), *IGFBP7* (4X), *KRT19* (4X), and *PLAUR* (6X) [223,224].

#### 3.3.10. Non-KCN DEG Superfamilies in E1–E70

Nine superfamilies of non-KCN DEGs were detected in E1–E70, with at least 15 occurrences, including 74X for SLC (Solute Carrier), 32X for COL (Collagen), 27X for ITG (Integrin), 22X for TMEM (Transmembrane), 21X for CD (Cluster of Differentiation), 19X for GPR (G-Protein-coupled Receptor), 18X for SOX (Sry-related HMG bOX), 15X for SERPIN (Serine Proteinase Inhibitors), and 15X for TNFRSF (Tumor Necrosis Factor Receptor Super Family).

The largest numbers of repeat DEG occurrences from superfamilies were *ITGA2* (previously mentioned, 7X), *SOX2* (5X), and *SLC1A2*/Excitatory Amino Acid Transporter 2 (EAAT2) (4X). EAAT2 clears glutamate from extracellular space at synapses [225,226,227]. Six Regulators of G protein Signaling (RGS) family members were found in seven studies. Eighteen (25.7%) of E1–E70 had either GPR- or RGS-family member DEGs. Among GPR DEG proteins, none are known as being H^+^-sensitive; see Appendix B (Text A1).

#### 3.3.11. KCN H^+^ Sensitivity in E1–E70 and Cancer Reviews

Ten reviews describing KCN family members in malignancy were compared with detection of KCN members in E1–E70. The numbers of KCN genes described in the reviews are as follows: 7 for glioblastomas [228], 3 in a report for a cancer colloquium [229], 23 for 18 cancer types [230], 13 for 10 cancer types [231], 9 for 17 cancer types [232], 21 for 14 cancer types [233], 12 for 13 cancer types [234], 15 for 7 cancer types [235], 13 for 13 cancer types [236], and 13 for 11 cancer types [237]. Twenty KCNs found in 35 (half) of the E1–E70 studies overlap with those in the reviews: *KCNA1*, *A3*, *A5*, *D1*, *H1*, *H2*, *H7*, *J5*, *J8*, *J10*, *K2*, *K3*, *K5*, *K15*, *MA1*, *N2*, *N3*, *N4*, *Q1*, and *T2*. The ten reviews combined with E1–E70 highlight KCN genes that encode H^+^-sensitive proteins. At least one KCN gene in each of 29 studies (82.9% of 35 overlapping E1–E70 studies) encodes an H^+^-sensitive protein; these studies are E4, E5, E6, E8, E9, E12, E13, E16, E22, E24, E28, E31, E32, E34, E36, E38–E40, E41, E47, E49, E52, E53, E58, E60, E64, and E68–E70. The remaining studies include E15, E29, E33, E45, E65, and E66. Citations for E1–E70 are given in Section 3.3.6.

#### 3.3.12. Long Introns in E1–E70’s KCN DEGs

Many KCN genes have long introns (>20,000 bp). Long introns may affect gene expression and regulation and can be genetically damaged. Thirty-one (58.5%) of 53 KCN DEGs in E1–E70 contain long introns, some very long. Seven KCN DEGs (*KCNC2*, *D3*, *H1*, *H8*, *MA1*, *Q1*, and *T2*) contain at least one intron 100,000–199,999 bp long. Five KCN DEGs (*KCNH7*, *IP1*, *Q3*, *MA1*, and *MB2*) contain at least one 200,000–299,999 bp intron and *KCNB2* and *KCNQ5* contain at least one intron 300,000–399,999 bp long. Fourteen KCN DEGs in E1–E70 contain multiple long introns. *KCNMA1* and *KCNT2* each contain seven long introns. *KCNN4* is among the KCN DEGs without any long introns. Forty-one (58.6%) of the E1–E70 studies contained at least one KCN DEG with at least one long intron. Nine studies had multiple (two to four) KCN DEGs with long introns. However, *KCNA3*, *A5,* and *F1* in E1–E70 do not contain any introns. Intron data from Ensembl was last accessed on 22 June 2025. The contrast between *KCNMA1* (seven long introns) and *KCNN4* (no long introns) is notable.

### 3.4. Study of Selected KCN DEGs in REMBRANDT

#### 3.4.1. Preliminary REMBRANDT Survey

The possibility of potassium acting as a chemoattractant in the CNS led to a search for KCN DEGs in the brain tumor database REMBRANDT while gathering the E1–E70 studies. Initial queries in the Online Mendelian Inheritance in Man (OMIM) database, 2018–2019, and literature descriptions of KCN genes [238], yielded 41 KCN genes with neuronal and astrocytic expressions. All 41 genes are in loci corresponding to known regions of chromosomal losses, gains, or both in glioblastomas [1]. These genes are distributed as follows: Chromosome (Ch) 1 (*KCNA2*, *A3*, *C4*, *N3*, *J9*, *J10*, *K2*, *K1*); Ch2 (*KCNJ3*, *J13*); Ch3 (*KCNMB2*, *MB3*); Ch5 (*KCNN2*, *MB1*); Ch6 (*KCNK17*); Ch8 (*KCNQ3*); Ch9 (*KCNT1*); Ch10 (*KCNMA1, K18*); Ch11 (*KCNJ11*, *C1*, *A4*, *K4*, *J5*); Ch12 (*KCNA1*, *A5*, *A6*, *J8*, *MB4*); Ch14 (*KCNK10*); Ch17 (*KCNJ12*, *J16*, *J2*); Ch19 (*KCNN1*, *N4*, *C3*); Ch20 (*KCNB1*, *Q2*); Ch21 (*KCNJ15*, *J6*); and Ch22 (*KCNJ4*). Most numerous were the KCNJ subfamily genes (*KCNJ2*, *3*, *4*, *5, 6*, *8*, *9*, *10*, *11*, *12*, *13*, *15*, and *16*) encoding inwardly rectifying potassium channel forming proteins and modulators/regulators; the Kir2.1, 3.1, 2.3, 3.4, 3.2, 6.1, 3.3, 4.1, 6.2, 2.2, 7.1, 4.2, and 5.1 proteins. Kir channels can be occluded by Mg^2+^ or polyamines when cell membrane potentials are positive relative to the equilibrium potential of K^+^, allowing only a small outward current. At membrane potentials that are negative relative to the equilibrium potential of K^+^, Mg^2+^ and polyamines leave the channels by flowing into the cell, so a large inward K^+^ current can occur [239,240]. Kirs, including Kir4.1, may also extrude K^+^. Kir 4.1 and 5.1 are present in astrocytes and oligodendrocytes [39,241].

A preliminary survey of REMBRANDT for expression of 21 KCN family members, 12 other potassium flow related genes, and 4 housekeeping genes (HKGs) checked for differences in glioblastomas from patients with and without a history of seizure medications. Seizures could be an indicator for abnormal levels of potassium [242,243,244]. With “unknowns for gene expression” included, there were 84 and 18 glioblastomas with and without a history of anti-seizure medication, respectively. Compared to the absolute difference in the expression of HKG (*PPIA*, *RPLP0*, *YWHAZ*, and *B2M*) between the two groups, single or average absolute median values of reporter probes for *KCNJ10*, *KCNK3*, *ATP1A1*, *KCNN3*, *KCNJ13*, *KCNMB2*, *SLC12A7*/KCC4, *KCNN2*, *KCNMB1*, *GJA1*, *KCND2*, *KCNK9*, *SLC1A2*/EAAT2, *KCNJ2*, and *KCNJ16* exceeded the average HKG difference (0.1131) of median values. Others, including *ATP1A2*, *KCNA2*, *KCNA3*, *KCNK1*, *KCNIP3*, *KCNMB3*, *SLC1A3*/EAAT1, *SLC12A2*/NKCC1, *KCNT1*, *KCNMA1*, *LRRC55*, *KCNA1*, *KCNMB4*, *GJB2*, *GJB6*, *SLC12A6*/KCC3, and *FXYD7*, did not exceed the average HKG difference in expression. Preliminary data suggested that the subset of glioblastomas in patients with seizures (medication history) had expressions of KCN family member genes that could be compared to the same genes in oligodendrogliomas (less invasive glial tumor) from patients also on seizure medications. Abnormalities in gene expressions among KCN family members resulting in potassium flux could be related to malignant behavior differences between oligodendrogliomas and glioblastomas.

#### 3.4.2. Gene Expressions Reflecting Cell of Origin

**Figure 6:**
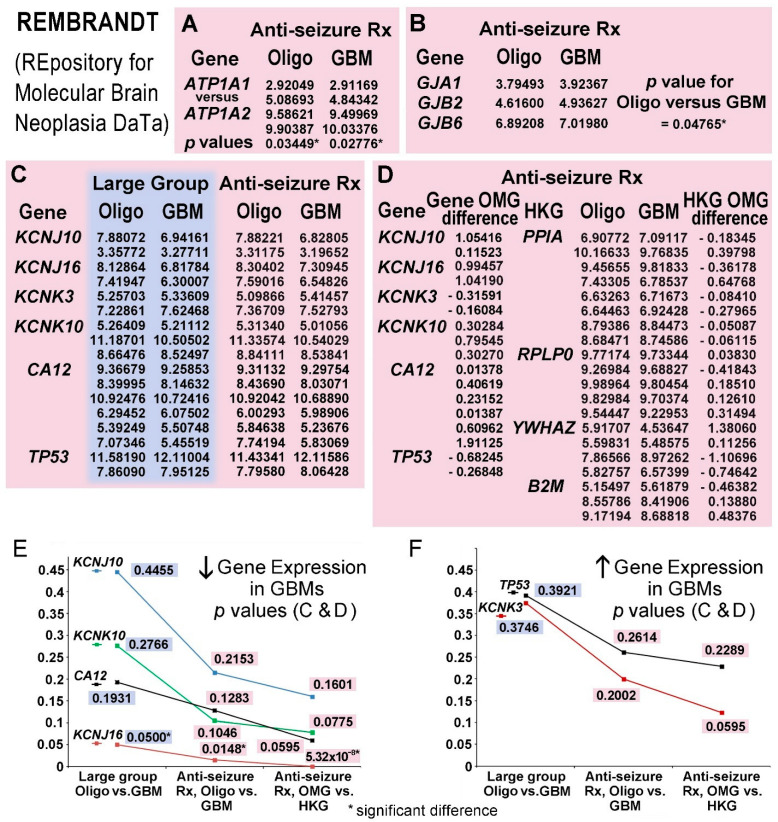
Results for oligodendrogliomas (Oligos) and glioblastomas (GBMs) from REMBRANDT. (**A**–**D**) Median reporter probe values of microarray gene expression results from Oligo and GBM tumor tissue are shown. Pink backgrounds (**A**–**F**) indicate results are from patients on anti-seizure medications (Anti-seizure Rx). Results with a blue background (C, E, F) are from large-group patients (medication history not used). (**A**) Comparisons of *ATP1A1*/Na/K-ATPase subunit alpha 1 (neural-specific) versus *ATP1A2*/Na/K-ATPase subunit alpha 2 (glial-specific) in Oligos and GBMs with significant differences confirmed glial origins. (**B**) Median reporter probe values for astrocytic-specific (versus oligodendroglial) values for gap junction genes with higher levels of expression in GBMs versus Oligos. (**C**) Values for median reporter probes for 4 KCN genes, *CA12*/carbonic anhydrase 12, and *TP53*/p53. (**D**) Another method to evaluate gene expression calculated Oligo-minus-GBM (OMG) differences of median probe results for each gene of interest and the housekeeping genes (HKGs). This method provided correction for variation in tissue handling, assay-to-assay variation within and between institutions, etc. (**E**,**F**) The *p* values are graphed for decreased and increased gene expressions in GBMs. Significance improved for the *KCNJ16* difference and trends strengthened for other genes. Oligo = oligodendroglioma, GBM = glioblastoma, Rx = medication, HKG = housekeeping gene. REMBRANDT (part of the Georgetown Database of Cancer (G-DOC)). * *p* < 0.05.

Checks for expected findings were performed. Expression of *ATP1A2* (alpha subunit of Na/K-ATPase specific for glial cells) was higher in both glial tumors versus neuronal-specific *ATP1A1* in patients on anti-seizure medications. The medians of two reporter probes available for each gene yielded significant differences, *p* = 0.0345 and 0.0278, respectively, for oligodendrogliomas and glioblastomas using two-tailed, unpaired *t*-tests (Figure 6A). Specificity of expression for connexin genes in astrocytic cells, *GJA1*/Cx43, *GJB6*/Cx30, and *GJB2*/Cx26, was used to verify expected cells of origin for the glial tumors, oligodendrogliomas, and glioblastomas [241,245,246] in REMBRANDT patients on anti-seizure drugs. The median values of single-reporter probes for expression of astrocytic-type connexins, collectively as a group, were significantly greater in glioblastomas; *p* = 0.0477 (one-tailed, paired t-test) (Figure 6B).

#### 3.4.3. KCN Gene Expression in Patients on Anti-Seizure Medications

Limiting tumor comparisons to patients on anti-seizure medications improved detection of differences between types of invasive glial tumors. Without using the history of seizure medications, a difference in expression of *KCNJ16*/Kir5.1 in glioblastomas (less expression), *p* = 0.05, became significant in glioblastomas using the smaller group of patients on anti-seizure drugs; *p* = 0.0148. Trends also improved for differences in expressions of *KCNJ10*/Kir4.1, *KCNK3*/TASK1, and *KCNK10*/TREK2 (all H^+^-sensitive, Appendix B (Table A2) and [247,248]) with reporter probe data (Figure 6C) and *p* values (Figure 6E,F); the same was also shown with *CA12*/carbonic anhydrase 12 and *TP53*/p53.

#### 3.4.4. Oligodendroglioma-Minus-Glioblastoma (OMG) Normalization

Housekeeping gene (HKG) normalization corrected for loss of genomic integrity, differences in tissue handling, assay-to-assay variation within and between institutions, etc., in comparisons for patients on anti-seizure medications. Oligodendroglioma-minus-glioblastoma (OMG) differences (increased or decreased) were calculated for each reporter probe’s median expression. These OMG differences for each gene of interest were compared to the OMG differences for all four HKGs, resulting in better *p* values (Figure 6D–F). The OMG difference for *KCNJ16*, decreased in glioblastomas, became highly significant, *p* = 5.532 × 10^−8^, and trends for other genes improved. These *t*-tests were calculated for unpaired samples with unequal variance. Median data for probes via Adobe Flash, shown in Figure 6A–D, are no longer available online (see Section 2.3).

### 3.5. KCN DEGs Shared in E1–E70, REMBRANDT, and Glioblastoma Cell Lines [22]

Whole-exome and RNA sequencing data of LN18, LN229, T98G, U87, U343, and U373 human glioblastoma cell lines in Patil et al.’s supplementary materials [22] revealed data for many KCN DEGs and genes of interest found in the E1–E70 and REMBRANDT studies. Also, three and five of their cell lines were used for the cell settling and migration studies, respectively, described earlier. Their data availability is described in the Data Availability Statement following this article’s text. The methodology of Patil et al. is described in [22], with some information included here. For each GBM cell line, a 62 Mb genome region was targeted. Alignment was to human reference genome hg19. A single-nucleotide variant (SNV) was a cancer-specific mutation (CSM) if present in the COSMIC database (reference 18 in [22]) and/or was present in GBM TCGA exome sequencing data (references 8 and 9 in [22]). Data for four cell lines, U87, T98G, LN229, and LN18, had an average concordance of 65.3% with COSMIC (reference 18 in [22]) and 74.7% with the Broad Institute’s CCLE database (reference 19 in [22]). Patil et al. reported small insertions and deletions (indels), −49 to +29 bases. Their differential gene expression data was obtained via comparisons of their RNA-seq data from the six GBM cell lines with expression data of five normal brain samples from TCGA, with a cut-off value of a 2-fold change in absolute expression. Their results for 2214 genes compared with those also present in TCGA GBM’s tumor samples had 73% concordance. Of the 2214 genes from cell lines, 1831 (82.7%) were downregulated. For the 1621 genes similarly up- or downregulated in both the TCGA and Patil et al. studies, 79.6% were downregulated.

**Figure 7:**
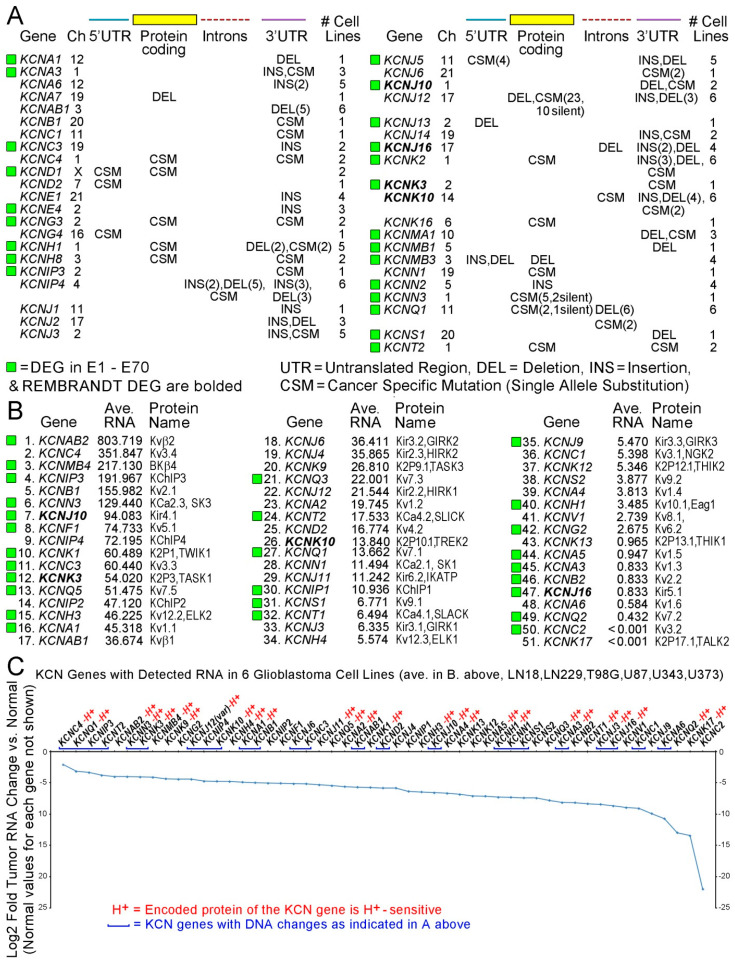
Many KCN DEGs detected in E1–E70 studies (Figure 3, Figure 4 and Figure 5) and in REMBRANDT (Figure 6) were included in whole-genome and expression studies of 6 glioblastoma cell lines, LN18, LN229, T98G, U87, U343, and U373, by Patil et al. [22]. For comparisons, shared KCN DEGs in E1–E70 (29 cancers including 7 glioblastomas) are marked with green squares and KCN genes of interest found in REMBRANDT are bolded (**A**,**B**). (**A**) Sequencing revealed DNA alterations including cancer-specific mutations (CSMs), insertions (INSs), and deletions (DELs). A total of 23 KCN DEGs (of 53 in E1–E70; see Figure 3 and Figure 4) and those of interest in REMBRANDT (3 with trends and 1 significantly differentially expressed; see Figure 6C–F) had changes in genomes of 6 cell lines reported by Patil et al. Several shared KCN DEGs were genetically altered in all 6 cell lines. (**B**) Average RNA expressions determined by Patil et al. are listed in descending order of abundance, including 27 and 4 of E1–E70 KCN DEGs and REMBRANDT’s KCN DEGs and genes of interest, respectively, with names of proteins. (**C**) Log2 fold RNA changes in combined tumor cell lines versus normal (see text) are shown with KCNs encoding H^+^-sensitive proteins indicated with red (H^+^) and KCNs with DNA changes in (**A**) with underlying blue brackets (˽). Of 51 KCNs in the glioblastoma cell lines, 27 (52.9%) encoded H^+^-sensitive proteins. Of 53 KCNs in E1–E70, 38 (71.7%) were found in their glioblastoma cell lines, with altered DNA and/or RNA expression. Of seven GBM studies in E1–E70, six (85.7%) shared 1–3 KCN DEGs with those described by Patil et al. [22]. See the text and Appendix B (Figure A5A) for specific glioblastoma studies in E1–E70. References for studies are in Section 3.3.6.

Forty-two KCN genes in their cell lines had CSMs and indels. DNA changes in 18 of the 42 KCN genes occurred in at least three glioblastoma cell lines. The KCNJ subfamily had the most members (ten) with DNA changes, including *KCNJ1*, *2*, *3*, *5*, *6*, *10*, *12*, *13*, *14*, and *16*. Among all KCN genes there were 69 CSMs, representing 4.3% of 1594 CSMs found in all genes sequenced, whereas 68 indels (25 insertions and 43 deletions) that they found in KCN genes represented 1.8% of all 3892 indels found among six glioblastoma cell lines. KCN DEGs in E1–E70 and genes of interest in REMBRANDT were among their KCN genes with CSMs, INSs, and DELs. Twenty-three of the E1–E70 KCN DEGs and four of the REMBRANDT study’s KCN genes of interest had changes described in genomes of six glioblastoma cell lines by Patil et al. Those detected in E1–E70 (glioblastomas (7) and 28 other cancers) are marked with green squares on the right-hand side of lists in Figure 7A, and genes of interest (significant expression difference or trends) found in REMBRANDT are bolded. All 51 expressed KCN genes detected in six glioblastoma cell lines were downregulated and represented 1.5% of all the 3428 differentially expressed genes in the cell lines. The average cell line KCN gene expression levels are listed by descending abundance, including 27 and 4 of those shared with E1–E70 and REMBRANDT, respectively, with the names of the encoded proteins in Figure 7B. *KCNJ16*, significantly downregulated in REMBRANDT’s glioblastomas compared to oligodendrogliomas, was among the genes with the lowest expression in cell lines studied by Patil et al. (Figure 7B). The log2 fold RNA changes from normal calculated using their supplementary table S6 [22] are graphed for 51 genes, with 27 genes (52.9%) that encode H^+^-sensitive proteins and 24 KCN genes with DNA changes highlighted, in Figure 7C. Of the 53 KCNs in E1–E70, there are 38 (71.7%) also described by Patil et al. with DNA changes and/or RNA expression detected. Of seven GBM studies in E1–E70, six (85.7%), E5, E21, E26, E27, E31, and E51, shared 1–3 KCN DEGs with those described by Patil et al. KCNs encoding proteins with H^+^ sensitivity in E1–E70 and REMBRANDT overlap with those described by Patil et al. (Figure 7C). References for H^+^ sensitivities are cited in Appendix B, Table A2, and in Section 3.4.3, for the encoded proteins of *KCNA1*, *KCNA3*, *KCNA5*, *KCNAB2*, *KCNH1*, *KCNH3*, *KCNJ10*, *KCNJ16*, *KCNK1*, *KCNK3*, *KCNMB4*, *KCNN3*, *KCNQ1*, *KCNQ2*, *KCNQ3*, *KCNQ5*, and *KCNT1*. References for the H^+^ sensitivity of proteins encoded by additional KCN genes in Figure 7C are as follows: *KCNA2*/Kv1.2 [249], *KCNA4*/Kv1.4 [250,251], *KCNC4*/Kv3.4 [252], *KCNH4*/ELK1 [253], *KCNJ3*/GIRK1 [254,255], *KCNJ11*/Kir6.2, IKATP [256,257], *KCNJ12*/Kir2.2, HIRK1 [258] (variant), *KCNK9*/K2P9, TASK3 [259,260], and *KCNK17*/K2P17.1, TALK2, TASK4 [261].

## 4. Discussion

### 4.1. Input of Functional U87 Studies

The decision to stochastically investigate potassium channel genes in cancer papers and a database was based on glioblastoma cell migration to serum and attraction to dying cells in settling assays with fluid deprivation. Tight cell adherence of robustly viable cells to dying settled cells was a surprise. Uncentrifuged filtered cancer cells from clinical fluid specimens can be viewed microscopically after dissolution of filters [262,263]. They do not have this appearance routinely. However, the clustering morphology of viable cells around single necrotic cells resembled rimming of deeply stained cells (“pseudopalisades”) around necrotic foci in glioblastoma tumor tissue, as described in the Introduction. Also, basophilic cells of tumors with central necrosis and more alkaline cytoplasmic pH detected in necrotic areas have been seen experimentally in cervical cancer [264]. Initial clues suggested potassium and pH involvement. Specifically, some viable glioblastoma cells rimming single dying cells, seemingly protected from the negative effect of fluid removal, had relatively darker staining, possibly due to an internal pH change, and as ATP is depleted in dying cells, K^+^ is released before cells rupture [265,266]. Potassium efflux has been seen in cancer cells [267].

A chemotactic role for potassium has been supported in numerous cell migration assays with a serum constituent acting as a chemoattractant. Also, U87 cells migrated significantly to a positive gradient of K^+^ generated by MEM, with improvement in the presence of FBS (Figure 2D). Flattening of dose curves and band-like rimming of glioblastoma cells around necrotic regions in tissue suggest that available ranges of potassium levels may need to match whatever functional complement of potassium channels is present in tumor cells. Elucidation of relationships in cancer cells between K^+^ and H^+^, possibly to incite reversal of pH, inspired investigation of KCN genes for differential expression in cancer studies and a brain tumor database.

### 4.2. Mechanistic Proposal for a New Route to pH Reversal

KCN family members, many of which are pH sensitive, cooperate to control potassium levels at cell membranes in a putatively H^+^-sensitive manner. The hypothesis is that K^+^, as a monovalent exchange cation for H^+^, frees bound protons from inner cell membranes for rapid cytoplasmic transfers to proton extruders, and instigates pH reversal in cancer if control of potassium flux is deregulated and rapid delivery of protons to cell exits is possible. The Grotthuss mechanism in protonated water wires is a new consideration to explain how freed protons rapidly transfer to cell exits to achieve pH reversal. Proton transfer via “water wires” has been described for proteins and the blood–brain barrier [268,269,270]. However, at this time cytoplasmic Grotthuss-type proton transfer in cancer cells has not been recognized, so direct mechanistic proof of K^+^/H^+^ exchange driving pH reversal is lacking, representing a limitation. However, as migrating tumor cells flatten and squeeze into small spaces, such as between a nerve fiber and its surrounding nerve sheath or along and then through dense basement membranes, bone, cartilage, etc., microaqueous intracellular regions are generated in tumor cells. As cell settling assays removed fluid to create a microaqueous condition for all cells, there was simultaneous release of K^+^ from single dying cells to surrounding viable cells. These centralized dying cells could also have become “sinks” for protons released from clustered viable tumor cells, possibly with some undergoing pH reversal to appear basophilic while dying central cells became more acidic.

Putatively, entry of excess K^+^ into viable cells exchanges with H^+^ bound to inner-membrane and cytosolic anions to release H^+^ (almost immediately H_3_O^+^) into surrounding cytoplasm containing Grotthuss water wires, that can rapidly supply protons to extruders, resulting in pH reversal and associated changes. In tumor tissue in vivo, the initiating potassium flux may be a response to stress or oncogenes to achieve pHi > pHe that acidifies the microenvironment for activation of proteases, etc., permitting cell migration and a higher cytosolic pH that activates alkaline-sensitive proteins, promoting cell fitness. An alkaline pHi leads to maximal tumor cell proliferation coupled with increased glycolysis and adaptation to hypoxia in silico [271] and is associated with increased flux of glycolytic enzymes, migration, and invasion experimentally [272]. Also, when pHi increases, H^+^ diffusion increases [273], possibly enhancing pH reversal for survival and dedifferentiation as single, migrating cells.

### 4.3. Potassium and KCN Genes in Cancer with Related Negative Findings

The KCN family offers a variety and range of dynamic distributions of K^+^ via numerous members, networking, biological milieus, modulation by oncogenes, histone/DNA modifiers, etc. Potassium is supplied by blood pulsing through the vasculature, although locally haphazard in many cancers, body fluids, and by nerves firing action potentials. Formation of synapses between tumor cells and neurons provides K^+^ as well as other synaptic substances [274]. Activation of proteases by extracellular acidosis that degrade connective tissues increases access to neural and vascular K^+^ to enhance migration and metastasis. Studies of KCN genes of interest in REMBRANDT, KCN cancer reviews, and six glioblastoma cell lines [22] included many genes encoding proteins with H^+^ sensitivity, to support similar findings in stochastic detection of KCN DEGs in unbiased E1–E70 cancer studies. KCNs in E1–E70 overlapped with most of the KCN genes of interest in REMBRANDT (*KCNJ16*, *KCNJ10*, and *KCNK3*,, all encoding H^+^-sensitive proteins), and with 20 KCN genes covered in the ten reviews. At least one KCN DEG in 29 of 35 studies from E1–E70 overlapping with KCNs in the ten reviews encodes an H^+^-sensitive protein. Also, in support of the REMBRANDT findings, *KCNJ16* was predicted to be negatively affected in glioblastoma cells by both Patil et al. and Clark et al. [22,139]. Potassium ions may redistribute in cancer cells to exchange with H^+^ in pH reversal via differential expression of KCN family members that encode H^+^-sensitive proteins, possibly responding to oncogenes, genetic damage, etc.

The E1–E70 studies allowed stochastic investigation of H+-related non-KCN DEGs associated with expression of KCN DEGs in malignancy to verify that genes encoding non-KCN components, needed in several steps of pH reversal, were relatively intact by detection of only a few or no DEGs that would be supportive. Multiple proton extruders have been proposed for glycolytic/plurimetabolic-type glioblastoma cells [275]. Adaptations for viability occur in cell settling assays and in some invasive scenarios without extracellular fluid being available. Aquaporin genes, *AQP1* and *AQP4*, whose products mediate water flux, were only found in three of the E1–E70 studies. *AQP3*, found in four studies, encodes aquaglyceroporin that mediates glycerol, uncharged solutes, and water flux [276]. In the E1–E70 studies, important negatives included non-KCN DEGs representing proton extruders, carbonic anhydrases, taurine and carnitine transporters, etc. Importantly, only a few studies found *ATP1A2*-encoding Na/K-ATPase as a DEG in E1–E70. Its role in pH reversal, as an active inward K^+^ transporter, is supported by a report that digoxin, an inhibitor of Na^+^/K^+^ ATPase, reduced the size of circulating tumor cell clusters in women with metastatic breast cancer by −2.2 cells per cluster [277].

In support of finding *KCNK* genes as DEGs in 20% of the E1–E70 studies, with *KCNK1*/K2P1 being most frequent, loss of *KCNK1* was associated with breast cancer cell survival when necrosis was induced [118]. Also, the ten reviews of KCNs in cancer that shared genes with those found in E1–E70, included four *KCNK*/K2P and four KCa subfamily (*KCNM* and *KCNN* groups) genes. Also, *KCNN4*/KCa3.1/IK/SK4 and *KCNMA1*/BK were the most frequently detected KCN DEGs in E1–E70.

### 4.4. E1–E70 Studies Related to Seven Oncogenes and Histone/DNA Modulation

Among the E1–E70 studies, 50 were in eight categories of oncogenes and histone/DNA modulator involvement, with some in multiple categories. Migration of seven glioblastoma cell lines responded to HGF; the ligand of *MET*’s encoded Met [278,279]. Although there was no correlation of Met with the level of HGF-stimulated migration detected, its role was not completely ruled out with additional studies (multiple Met antibodies, etc.). Others have proposed a role for multiple oncogenes, including *EGFR*, *RAS* (*KRAS* and *HRAS*), *MYC*, and *TGFβ*, that overlap with oncogenes involved in E1–E70, to interact on paths to malignancy in a context-driven manner [280]. Lactate can activate *TGFβ* [281,282,283,284]. Also, “upregulation of oncogenic pathways” has been described in clinically evident precancerous lesions of 12 different types of malignancies [285]. The collective influence of multiple oncogenes on downstream potassium channels may promote K^+^ flux via H^+^-sensitive KCN proteins. KCN studies related to seven oncogenes and/or histone/DNA modulation were significantly (*p* = 0.0325) more likely to have at least one or more KCN DEG encoding an H^+^-sensitive protein than the other KCN studies in “no category”. Although oncogenes may promote K^+^/H^+^ exchange, leading to pH reversal, this has not been proven.

Retrieval of citation data from PubMed revealed a significant correlation in the numbers of studies of H^+^ with K^+^ across all eight categories of the seven oncogenes and histone in two-word searches without restriction to cancer. However, the category for MET had to be removed to achieve a comparable correlation when searches were restricted to “cancer”. Although any explanation requires definitive studies, this may reflect under-reporting of negative results, and this study also found unexpected negative results for Met in HGF-stimulated migration studies. Also, the PubMed citations for BK (*KCNMA1* encoded) probably constituted a large proportion (approximately one-third) of studies involving K^+^ and H^+^ across the categories of seven oncogenes and “histone” not restricted to cancer and most of the studies when restricted to “cancer”. The stochastic findings in this study suggest that at least some other H^+^-sensitive KCN DEGs are also influenced by oncogenes in H^+^ dynamics.

### 4.5. Non-KCN DEGs in Multiple E1–E70 Studies

In E1–E70, the most frequent non-KCN DEGs found were *ITGA2, HMGA2*, *LIF*, and *PLAUR*, with each found in six to seven studies. To support the relevance of cancer gene expression studies with unbiased detection of KCN DEGs, six DEGs, i.e., *FGFR3*, *GDF15*, *IGFBP3*, *IGFBP7*, *KRT19*, and *PLAUR*, each included in at least four of the E1–E70 studies, were also identified by others as encoding circulating proteins associated with a risk of cancer [223,224]. *PLAUR*, found in six of the E1–E70 studies, was co-expressed with the most frequent KCN genes that are also H^+^-sensitive, *KCNN4* and *KCNMA1* in four and two studies, respectively. *KCNN4*, *PLAUR*, and *PLAU* were detected together as DEGs in a study associated with three categories, RAS, TP53, and His/Chr/Epi. BK (*KCNMA1*) citations in PubMed correlated with these for PLAUR + PLAU and ITGA2. Co-expression of non-KCN DEGs may become relevant functionally and/or as a signature of K^+^ dynamics if they occur in additional studies.

### 4.6. Specific Roles for Redistribution of K^+^ in Cancer

In studies on intracellular pH-regulating systems, potassium channel inhibitors have been used with interesting results, including “slower recovery from CO_2_ acidification” in early frog skeletal muscle experiments [286]. Potassium channels have been recognized as playing important roles in cancer, including volume changes [287,288], but without also considering K^+^ exchange with H^+^ on fixed anions as inciting pH reversal. Movement of hydrated potassium (K^+^-7H_2_O) from cell interiors to inner-membrane regions may redistribute water via outward flux of K^+^ requiring separation from its loosely aggregated water molecules to achieve its passage through KCN channels. Large outward (*KCNMA1*/BK), intermediate outward (*KCNN4*/IK), and bidirectional (*KCNJ10*-*KCNJ16*/Kir4.1-Kir5.1 or Kir4.1 only) fluxes, etc., may alter distributions of both K^+^ and H_2_O. Simultaneous changes in Cl^−^ channels (CLCN genes) were detected only once (*CLCN7* as a DEG) in E1–E70. Aquaporin participation seemed unlikely in E1–E70 stochastically. Na^+^ binds water tightly. Redistribution of water regionally within cells may promote Grotthuss conditions near inner membranes to aid pH reversal but this is unknown. Additionally, redistribution of K^+^ away from mitochondria within cell interiors may affect mitochondrial K^+^-dependent enzymes to influence malignant behavior. The results of the E1–E70 studies only revealed gene expression that crossed thresholds set by the study authors for significance. Lower gene expression levels may collectively also be biologically relevant in a network of KCN-encoded proteins mediating potassium flux but would require bioinformatic searches of supplemental data.

## 5. Conclusions

Migration and settling assays provided data to support and focus further investigation of potassium flux in cancer. Although pH reversal in cancer cells generated via proton export by multiple extruders has already been established, if initiating events involve potassium, then therapeutic options may widen. Multiple tumor models and study approaches have produced stochastic evidence for potassium flux via the KCN family playing an H^+^-related role in at least a subset of malignancies. Therapeutic suggestions regarding alterations of potassium channels carry concern regarding effects on the heart [289]. However, potassium channels are already being targeted in cancer [290]. Any potential therapeutic benefit through diet is considered since foods rich in potassium salts of metabolizable anions, such as citrate, etc., characteristic of fruits and vegetables, should deplete hydrogen ions and have an alkalizing effect [291]. The stochastic view of cancers based on unbiased studies detecting KCN genes incidentally in genomic landscapes suggests that encoded H^+^-sensitive KCN proteins may be downstream effectors of one or more drivers in cancer. Oncogenes may promote adaptation to adverse events and genetic damage to cells, including KCN genes, to optimize cell fitness for survival, possibly as a spectrum of ongoing changes that include the extreme outcome of becoming dedifferentiated migrating cells, via pH reversal. Also, cancer-specific structural alterations of surface proteins encoded by genetically flawed KCN genes may add to the repertoire of targets for immune attack in personalized medical treatments. Author curated, unbiased, published gene landscapes in complex cancer studies are useful for stochastically identifying candidates in the large KCN gene family that may encode downstream effectors of oncogenes that may initiate pH reversal. Also, tumor models for KCN genes and encoded proteins are identified, where significant changes in KCN gene expression have been seen in many types of malignancy.

## Data Availability

Data generated in this study will be available upon request from the author. Please note that data from REMBRANDT per Adobe Flash (not available after 31 December 2020) can be seen in Figure 6, except for a few instances described, which can be made available on request. KCN gene data obtained from the published manuscript by Patil, V. et al. [22] follows the terms of their statement “an open access article distributed under the terms of the Creative Commons Attribution License, which permits unrestricted use, distribution, and reproduction in any medium, provided the original author and source are credited”. Of the papers used in the E1–E70 studies, 34%, 31%, 13%, and 6% were found in *Cancer Research*, *Cancer Cell*, *Nature Communications*, and *Journal of Molecular Diagnostics*, respectively. Others were from 11 journals, one paper each. Papers for E1–E70 are cited in Section 3.3.6.

## Supplementary Materials

The following supporting information can be downloaded at https://www.mdpi.com/article/10.3390/biom15081177/s1, Table S1: FullTblB1DEGalphaAZ.

## Abbreviations

The following abbreviations are used in this manuscript.

ACLY	ATP citrate lyase
Arg1	Arginase 1
AS	Anti-seizure
BK	Big K (encoded by *KCNMA1*)
BSA	Bovine serum albumin
CD	Cluster Differentiation
*CLCN*	Chloride Channels gene family, *CLCN1*,*2*,*3*, etc.
COL	Collagen
CSM	Cancer-specific mutation
Cx	Connexin
DEG	Differentially expressed gene
E	Expression
EAAT	Excitatory Amino Acid Transporter
EAG1	Ether-A-Go-Go-K^+^ channel protein 1
ELK1,2	EAG-like K^+^ channel proteins 1,2
EMT	Epithelial mesenchymal transition
ERG1,3	EAG-related gene K^+^ channel proteins 1,3
FBS	Fetal bovine serum
GABA	Gamma-aminobutyric acid
GBM	Glioblastoma
G-DOC	Georgetown Database of Cancer
Girk3,4	G protein-activated inward rectif-ier K^+^ channel proteins, 3,4
GPR	G-protein-coupled receptor
G-proteins	Guanosine-triphosphate-binding proteins
GWATERW	Grotthuss water wire
HGF	Hepatocyte growth factor
His/Chr/Epi	Histone/chromatin/epigenetic
HKG	Housekeeping gene
IK	Intermediate K (encoded by *KCNN4*)
Indels	Insertions and deletions
ITAG2	Integrin 2
ITG	Integrin
K2P	Tandem 2 pore domain KCN subfamily proteins
Kca	Calcium-stimulated KCN subfamily proteins
Kchip1,3	Kv channel interacting proteins 1,3
*KCNA*	Subgroup of KCN genes, *KCNA1*,*2*,*3*, etc.
*KCNAB*	Subgroup of KCN genes, *KCNAB1*,*2*,*3*
*KCNB*	Subgroup of KCN genes, *KCNB1*,*2*
*KCNC*	Subgroup of KCN genes, *KCNC1*,*2*,*3*, etc.
*KCND*	Subgroup of KCN genes, *KCND1*,*2*,*3*
*KCNE*	Subgroup of KCN genes, *KCNE1*,*2*,*3*, etc.
*KCNG*	Subgroup of KCN genes, *KCNG1*,*2*,*3*, etc.
*KCNH*	Subgroup of KCN genes, *KCNH1*,*2*,*3*, etc.
*KCNIP*	Subgroup of KCN genes, *KCNIP1*,*2*,*3*, etc.
*KCNJ*	Subgroup of KCN genes, *KCNJ1*,*2*,*3*, etc.
*KCNK*	Subgroup of KCN genes, *KCNK1*,*2*,*3*, etc.
*KCNM*	Subgroup of KCN genes, *KCNM*,*A1*,*B1*,*B2*, etc.
*KCNN*	Subgroup of KCN genes, *KCNN1*,*2*,*3*, etc.
*KCNQ*	Subgroup of KCN genes, *KCNQ1*,*2*,*3*, etc.
*KCNS*	Subgroup of KCN genes, *KCNS1*,*2*,*3*, etc.
*KCNT*	Subgroup of KCN genes, *KCNT1*,*2*
Kir	Inwardly directing KCN subfamily proteins
Kv	Voltage-dependent KCN subfamily proteins
LDH	Lactate dehydrogenase
LIF	Leukemia Inhibitory Factor
MCT	Monocarboxylate transporter
MEM	Minimal Essential Media
Mes	Mesenchymal
MiRP	MinK (minimal K^+^channel Protein-related peptide)
Na/K ATPase	Sodium/potassium ATPase
NOX	NADPH oxidases
Oligo	Oligodendroglioma
OMG	Oligodendroglioma minus glioblastoma
OMIM	Online Mendelian Inheritance in Man
PBS	Phosphate-buffered saline
pHe	Extracellular pH
pHi	Intracellular pH
PIP2	Phosphatidyl inositol 4,5 bisphosphate
R	Pearson product-moment correlation
REMBRANDT	Repository of Molecular Brain Neoplasia Data
RGS	Regulator of G protein signaling
ROS	Reactive oxygen species
RRID	Research Resource Identifier
SERPIN	Serine proteinase inhibitors
SNV	Single-nucleotide variant
SK	Small K (encoded by some *KCNN* genes)
Slack	Sequence like a calcium-activated K^+^ channel protein
SLC	Solute Carrier
Slick	Sequence like an intermediate conductance K^+^ channel protein
SOX	Sry-related HMG BOX
TasK1,2,5	Twik-related acid-sensitive K^+^ channel proteins 1,2,5
Thik2	Tandem pore domain halothane-inhibited Inhibited K^+^ channel protein 2
TMEM	Transmembrane
TNFRSF	Tumor Necrosis Factor Receptor Super Family
Trek1	Twik-related K^+^ channel protein 1
TwiK1,2	Tandem pore domain in weak inward rectifying K^+^ channel proteins 1,2
uPA	Urokinase-type plasminogen activator

## Appendix B

**Table A1:** A sample of alphabetized differentially expressed genes (DEGs) in E1–E70 studies. See Supplementary Materials for the entire DEG list; Table S1: FullTblB1DEGalphaAZ can be downloaded as a Word file at Table S1.

DEG Name	Study Number
A2M	E21
AAK1	E65
AATK	E54
AB113AP	E1
ABCA1	E50
ABCA12	E54
ABCB10	E48
ABCB1A	E69
ABCC3	E70
ABCD1	E31
See full list…	See full list…

**Table A2:** KCN genes in E1–E70 studies that encode H^+^-sensitive proteins.

KCN Gene	Protein	Reference
*KCNA1*	Kv1.1	[292]
*KCNA3*	Kv1.3	[293,294]
*KCNA5*	Kv1.5	[295,296,297,298,299,300,301]
*KCNAB2*	Kvbeta2	[302,303]
*KCND3*	Kv4.3	[304,305,306]
*KCNE2*	MiRP1	[87,307,308,309,310,311]
*KCNE3*	MiRP2, R83H variant	[252]
*KCNH1*	Kv10.1, EAG1	[253,312]
*KCNH2*	Kv11.1, HERG1	[253,313,314,315,316,317,318,319,320,321,322,323,324,325]
*KCNH3*	Kv12.2, ELK2	[253]
*KCNH8*	Kv12.1, ELK3	[253,326]
*KCNJ5*	Kir3.4, GIRK4	[254,255,327]
*KCNJ8*	Kir6.1, KATP-1	[327,328,329,330,331,332,333]
*KCNJ10*	Kir4.1, BIRK10	[334,335,336]
*KCNJ10/KCNJ16*	Kir4.1/Kir5.1heterodimer	[72,326,337,338,339,340,341,342,343]
*KCNJ13*	Kir7.1	[72,327,344]
*KCNK1*	K2P1, TWIK1	[343,345,346,347,348,349,350]
*KCNK2*	K2P2, TREK1	[343,348,351,352,353,354,355,356,357]
*KCNK3*	K2P3, TASK1	[343,346,348,355,358,359,360,361]
*KCNK5*	K2P5, TASK2	[236,343,346,348,362,363,364,365,366,367,368,369]
*KCNK6*	K2P6, TWIK2	[346,370,371]
*KCNK15*	K2P15, TASK5	[372]
*KCNMA1*	KCa1.1, BK, Slo1, maxiK	[96,373,374,375,376,377,378,379,380,381,382,383,384,385,386,387,388]
*KCNMB4*	BKbeta4	[389,390,391,392,393]
*KCNN2*	KCa2.2, SK2	[394]
*KCNN3*	KCa2.3, SK3	[394]
*KCNN4*	KCa3.1, SK4, Gardos	[395,396,397,398]
*KCNQ1*	Kv7.1, KvLQT1	[85,236,307,308,309,311,399,400,401,402,403,404,405]
*KCNQ2*	Kv7.2	[402] (described data) [406,407]
*KCNQ3*	Kv7.3	[406]
*KCNQ5*	Kv7.5	[408]
*KCNT1*	KCa4.1, Slo2.2, KNa1.1	[409,410,411,412]
*KCNU1*	KCa5.1, Slo3, KCNMA3	[389,412,413,414,415,416,417,418]

**Text A1.** Potential interactions of KCN proteins with H^+^-sensitive G protein-coupled receptors. Multiple KCN proteins have interactions with four G protein-coupled receptors encoded by *GPR4*, *65*, *68*, and *151*, that are among six reported to be H^+^-sensitive [419,420,421,422,423,424,425]. H^+^-sensitive proteins encoded by GPR genes are found in many types of tumors [423,424,426,427]. Functional complementation between proteins encoded by *GPR4* and *KCNK5*/TASK2 [427,428,429] and related expression profiles of proteins encoded by *GPR31*, *GPR151*, *KCNK3*/TASK1, and *KCNK9*/TASK3 have been found in tumors [425]. STRING interactions (Gene Cards, 17 October 2024) of proteins encoded by *GPR4*, *65*, *68*, and *151* as a group included proteins encoded by nine KCNs: *KCNJ8*, *K1, K2, K4, K5*, *K10*, *K16*, *K17*, and *K18*. Among these, 8 of the 70 studies (E3, E8, E22, E24, E31, E35, E61, E68) detected *KCNJ8*, *K1, K2,* and *K5* as DEGs. Also, although *GPR39*’s encoded protein is not reported to be H^+^-sensitive, it interacts with the four H^+^-sensitive proteins encoded by *GPR4*, *65*, *68*, and *132*. *GPR39* is a DEG found in 2 of the 70 studies, E14 [193] and E60 [176], that also detected *KCNQ5* and *KCNMA1*, respectively. The ligand of the protein encoded by *GPR39* is encoded by *GPNMB* [430], and *GPNMB* was a DEG in three studies: E26 [212], E29 [157], and E49 [166]. *GPR35*’s protein (also not H^+^-sensitive) interacts with *GPR4*’s encoded H^+^-sensitive receptor protein. *GPR35* is a DEG that was found in two studies that found *KCNG2* and *KCNE3*, E20 [210] and E42 [217], respectively. The ligand for *GPR35*’s receptor protein is kynurenic acid [431]. *GPR84*’s protein (also not H^+^-sensitive) interacts with *GPR65*’s encoded H^+^-sensitive receptor protein. *GPR84* is a DEG that was found in two studies, with *KCNK6* found in E11 [173] and *KCNK12* and *KCNU1* in E23 [201]. The ligands for *GPR84*’s receptor protein are medium-chain fatty acids [432]. Thirteen of the 14 studies (out of 70) with potential indirect GPR protein sensitivity to H^+^ also detected KCN DEGs that encode H^+^-sensitive proteins. Potentially, KCN DEG-encoded proteins in 13 (18.6%) of the E1–E70 studies have indirect involvement with H^+^-sensitive G protein-coupled receptor proteins via STRING interactions (Gene Cards, 17 October 2024) or as described above according to the cited literature. They may function collaboratively with GPR-encoded H^+^-sensitive proteins in tumors.
