# Supplementary material for: Cell Settling, Migration, and Stochastic Cancer Gene Expression Suggest Potassium Membrane Flux May Initiate pH Reversal
Source: Biomolecules. 2025 Aug 16;15(8):1177. doi: 10.3390/biom15081177 (PMC12384032; doi:10.3390/biom15081177)
Supplement: Supplementary file 1 [file biomolecules-15-01177-s001.zip › biomolecules-3758455-supplementary.pdf]

1	E21	A2M
2	E65	AAK1
3	E54	AATK
4	E1	AB113AP
5	E50	ABCA1
6	E54	ABCA12
7	E48	ABCB10
8	E69	Abcb1a
9	E70	Abcc3
10	E31	ABCD1
11	E9	ABHD11
12	E58	AC004019.18
13	E14	AC012501.3
14	E58	AC079602.1
15	E25	AC092162.1
16	E33	ACAA2
17	E21	ACACA
18	E24	ACAN
19	E69	Ace
20	E20	ACSM1
21	E2	ACSM3
22	E29	ACTA2
23	E1	ACTA2
24	E43	ACTA2
25	E66	ACTA2
26	E45	Actb
27	E4	ACTC1
28	E1	ACTG2
29	E21	ACTG2
30	E43	ACTG2
31	E4	ACTN2
32	E16	ACTR3
33	E35	ACTR3
34	E59	Actr3b
35	E1	ACVR2A
36	E70	Acvrl1
37	E38	ADAM12
38	E39	ADAM12
39	E21	ADAM19
40	E38	ADAM19
41	E43	ADAM19

42	E28	ADAM28	
43	E49	Adam8	
44	E45	Adam9	
45	E49	Adamdec1	
46	E14	ADAMTS15	
47	E1	ADAMTS2	
48	E21	ADAMTS4	
49	E26	Adarb1	
50	E26	Adcy1	
51	E4	ADCY2	
52	E26	Adcy5	
53	E4	ADCY6	
54	E4	ADCY8	
55	E62	ADCY9	
56	E34	ADCYAP1	
57	E5	ADCYAP1R1	
58	E27	ADD2	
59	E21	ADGRG1	
60	E34	ADIPOR2	
61	E15	ADORA2A	
62	E38	ADRA1D	
63	E2	ADRA2A	
64	E52	ADRA2A	
65	E40	AEBP1	
66	E30	AFAP1-AS1	
67	E2	Afap1l1	
68	E25	AFAP1L1	
69	E23	AFAP1L2	
70	E14	AFMID	
71	E21	AGAP2	
72	E57	AGBL2	
73	E20	AGFG2	
74	E50	AGO2	
75	E14	AGO3	
76	E58	AGPAT9	
77	E2	AGR3	
78	E42	AGR3	
79	E31	AGRN	
80	E34	AGT	
81	E51	AGT	
82	E34	AGTR1	
83	E70	Agtr1	
84	E26	Ahl1	

85	E21	AHRR	
86	E37	AICDA	
87	E54	AICDA	
88	E21	AIFM1	
89	E23	AIM2	
90	E14	AJ239322.1	
91	E70	Ak1	
92	E70	Ak5	
93	E15	AK8	
94	E37	AKAP12	
95	E38	AKAP12	
96	E31	AKAP13	
97	E16	AKR1B1	
98	E45	Akr1b10	
99	E45	Akr1b3	
100	E45	Akr1b8	
101	E23	AKR1C2	
102	E42	AKR1C3	
103	E23	AKR1C4	
104	E35	AKT3	
105	E52	AL354984.1	
106	E21	ALDH18A1	
107	E54	ALDH1A1	
108	E20	ALDH1A2	
109	E49	Aldh1a2	
110	E54	ALDH1A2	
111	E1	ALDH1A3	
112	E69	Aldh1a3	
113	E57	ALDH1L1	
114	E21	ALDH3B1	
115	E44	ALG3	
116	E2	ALMS1	
117	E43	ALOX5AP	
118	E64	ALOX5AP	
119	E54	ALPPL2	
120	E21	AMDHD2	
121	E55	Amhr2	
122	E11	AMIG02	
123	E13	AMN	
124	E58	AMOT	
125	E20	AMOTL2	
126	E70	Amotl2	
127	E2	ANGPT2	

128	E1	ANGPTL2	
129	E29	ANGPTL4	
130	E33	ANGPTL4	
131	E43	ANGPTL4	
132	E69	Angptl4	
133	E65	ANGPTL7	
134	E20	ANKRD1	
135	E31	ANKRD46	
136	E20	ANKRD7	
137	E63	ANLN	
138	E70	Anln	
139	E66	ANP32B	
140	E41	ANTXR2	
141	E56	ANTXR2	
142	E41	ANXA1	
143	E70	Anxa1	
144	E49	Anxa10	
145	E69	Anxa10	
146	E42	ANXA13	
147	E60	ANXA13	
148	E37	ANXA2	
149	E45	Anxa2	
150	E70	Anxa3	
151	E29	ANXA4	
152	E16	ANXA6	
153	E21	ANXA6	
154	E40	ANXA8L2	
155	E22	ANXA9	
156	E58	AOC1	
157	E58	AP001626.1	
158	E44	AP2M1	
159	E48	APBA2	
160	E66	APC2	
161	E32	APCDD1	
162	E56	APCDD1	
163	E34	APLN	
164	E37	APOBEC3G	
165	E26	Apod	
166	E1	APOE	
167	E14	APOE	
168	E21	APOE	
169	E59	Apoe	
170	E14	APOL3	

171	E2	APP	
172	E2	AQP1	
173	E51	AQP1	
174	E59	Aqp1	
175	E25	AQP3	
176	E8	AQP3	
177	E22	AQP3	
178	E58	AQP3	
179	E51	AQP4	
180	E54	AR	
181	E65	AR	
182	E70	Arap3	
183	E21	ARC	
184	E51	ARC	
185	E52	AREG	
186	E35	ARF4	
187	E43	ARF4	
188	E21	ARFGEF3	
189	E1	ARHGAP20	
190	E35	ARHGAP20	
191	E1	ARHGAP24	
192	E40	ARHGAP25	
193	E58	ARHGAP26	
194	E43	ARHGDIB	
195	E3	ARHGEF16	
196	E27	ARHGEF26-AS1	
197	E67	Arid5b	
198	E41	ARL4C	
199	E14	ARL4C	
200	E4	ARL6IP5	
201	E1	ARMH4	
202	E23	ARNTL2	
203	E38	ARNTL2	
204	E7	ARRB1	
205	E62	ARRDC3	
206	E31	ARSG	
207	E58	ARSI	
208	E65	ARX	
209	E31	ASAH1	
210	E20	ASAP1	
211	E12	ASCL1	
212	E17	ASCL1	
213	E35	ASCL1	

214	E65	ASCL1	
215	E2	ASCL2	
216	E46	ASCL2	
217	E15	ASIC4	
218	E54	ASNS	
219	E2	ASPM	
220	E17	ATCAY	
221	E60	ATF3	
222	E35	ATG14	
223	E60	ATM	
224	E66	ATN1	
225	E26	Atp1a2	
226	E4	ATP1A2	
227	E5	ATP1A2	
228	E51	ATP1A2	
229	E29	ATP1B1	
230	E19	ATP1B1	
231	E51	ATP1B2	
232	E15	ATP2B1	
233	E58	ATP2C2	
234	E66	ATP5D	
235	E70	Atp6v0e1	
236	E48	ATP6V1C1	
237	E42	ATP7B	
238	E25	AURKA	
239	E67	Auts2	
240	E58	AVPI1	
241	E34	AVPR2	
242	E68	AXIN2	
243	E41	AXL	
244	E14	B2M	
245	E67	B2m	
246	E21	B4GALT5	
247	E33	B4GALT5	
248	E54	B4GALT6	
249	E41	BAALC	
250	E42	BACE2	
251	E56	BACE2	
252	E22	BAG1	
253	E13	BAIAP2L1	
254	E42	BAIAP2L2	
255	E63	BAIAP2L2	
256	E55	Bambi	

257	E62	BANF1	
258	E37	BANK1	
259	E56	BANK1	
260	E46	BARX2	
261	E23	BATF3	
262	E70	Bbc3	
263	E60	BBC3(PUMA)	
264	E70	Bbp4	
265	E59	BC064078	
266	E26	Bc1	
267	E60	BCAM	
268	E14	BCAN	
269	E35	BCAR3	
270	E60	BCAS3	
271	E63	BCAT1	
272	E21	BCAT2	
273	E20	BCL11A	
274	E12	BCL2	
275	E22	BCL2	
276	E35	BCL2	
277	E70	Bcl2	
278	E70	Bcl2l1	
279	E1	BCL3	
280	E2	BCL7A	
281	E14	BCL7A	
282	E37	BCL7A	
283	E46	BCOR	
284	E66	BCORL1	
285	E70	Bdnf	
286	E65	BEND2	
287	E14	BEND3	
288	E14	BEND6	
289	E26	Bgn	
290	E21	BHLHE40	
291	E20	BICC1	
292	E58	BICC1	
293	E1	BIK	
294	E49	Birc3	
295	E60	BIRC3	
296	E20	BIRC5	
297	E63	BIRC5	
298	E14	BIRC7	
299	E39	BIRC7	

300	E3	BLNK	
301	E15	BLVRA	
302	E38	BMP2	
303	E41	BMP2	
304	E49	Bmp2	
305	E60	BMP4	
306	E5	BMP7	
307	E56	BMX	
308	E40	BNC1	
309	E29	BNIP3	
310	E16	BNIP3	
311	E66	BOLA2B	
312	E15	BRAF	
313	E2	BRCA2	
314	E66	BRD4	
315	E34	BRN3A	
316	E15	BRSK2	
317	E33	BSG	
318	E11	BSLC18A2	
319	E41	BST1	
320	E67	Bst2	
321	E11	BTBD11	
322	E69	Btc	
323	E54	BTG2	
324	E60	BTG2	
325	E66	BTG2	
326	E53	BTK	
327	E15	BTN2A2	
328	E42	BTNL8	
329	E54	C10orf10	
330	E58	C10orf99	
331	E65	C11orf87	
332	E14	C11orf96	
333	E23	C12orf39	
334	E23	C12orf56	
335	E41	C12orf75	
336	E29	C15orf48	
337	E23	C15orf48	
338	E41	C16orf45	
339	E40	C16orf74	
340	E43	C17orf91	
341	E14	C1orf115	
342	E66	C1orf173	

343	E21	C1orf95	
344	E34	C1QBP	
345	E58	C1QC	
346	E56	C1QTNF1	
347	E23	C20orf70	
348	E58	C2CD4B	
349	E34	C3AR1	
350	E54	C4orf34	
351	E45	C5orf51	
352	E11	C6orf192	
353	E58	C9orf152	
354	E29	CA12	
355	E19	CA12	
356	E21	CA14	
357	E42	CA2	
358	E29	CA9	
359	E69	Cab39l	
360	E58	CACNA1A	
361	E4	CACNA1B	
362	E34	CACNA1C	
363	E4	CACNA1E	
364	E11	CACNA2D3	
365	E58	CACNB1	
366	E3	CACNB3	
367	E28	CADM1	
368	E29	CADM1	
369	E41	CADM1	
370	E41	CADM4	
371	E58	CADPS	
372	E23	CALB1	
373	E57	CALB1	
374	E26	Calb2	
375	E52	CALCA	
376	E60	CALD1	
377	E21	CALM1	
378	E50	CALM1	
379	E31	CALM3	
380	E30	CALML3-AS1	
381	E26	Calml4	
382	E42	CALML4	
383	E62	CALU	
384	E51	CAMK2B	
385	E58	CAMK4	

386	E26	Campk2a	
387	E16	CAP1	
388	E21	CAPN5	
389	E59	Car2	
390	E60	CARD10	
391	E26	Cartpt	
392	E34	CASK	
393	E59	Casp1	
394	E37	CASP3	
395	E44	CASP4	
396	E20	CAT	
397	E23	CATSPER1	
398	E66	CATSPERD	
399	E28	CAV1	
400	E41	CAV1	
401	E60	CAV1	
402	E20	CAVIN2	
403	E50	CBL	
404	E45	Cbr1	
405	E45	Cbr3	
406	E69	Cbr4	
407	E60	CBS	
408	E31	CBX5	
409	E2	CC2D2A	
410	E20	CCDC114	
411	E26	Ccdc136	
412	E26	Ccdc153	
413	E2	CCDC18	
414	E54	CCDC33	
415	E54	CCDC46	
416	E66	CCDC85B	
417	E60	CCDN1	
418	E26	Cck	
419	E11	CCL1	
420	E49	Ccl20	
421	E68	CCN3	
422	E22	CCND1	
423	E35	CCND1	
424	E45	Ccnd1	
425	E70	Ccnd1	
426	E37	CCND2	
427	E45	Ccnd2	
428	E65	CCND2	

429	E53	CCND3	
430	E35	CCNE1	
431	E60	CCNE2	
432	E21	CCNG1	
433	E33	CCNL1	
434	E66	CCNL2	
435	E28	CCR10	
436	E18	CCT2	
437	E41	CD163L1	
438	E14	CD163L1	
439	E28	CD19	
440	E28	CD22	
441	E53	CD22	
442	E28	CD24	
443	E58	CD247	
444	E23	CD274	
445	E53	CD36	
446	E28	CD38	
447	E54	CD4	
448	E28	CD40	
449	E13	CD44	
450	E45	Cd44	
451	E34	CD55	
452	E29	CD68	
453	E23	CD70	
454	E28	CD79A	
455	E28	CD79B	
456	E37	CD86	
457	E23	CD8A	
458	E41	CD96	
459	E11	CD99	
460	E23	CDA	
461	E14	CDC16	
462	E20	CDC20	
463	E63	CDC20	
464	E21	CDC25B	
465	E2	CDC42EP1	
466	E60	CDC42EP3	
467	E2	CDCA7	
468	E14	CDCA7	
469	E2	Cdca7l	
470	E50	CDCP1	
471	E16	CDH1	

472	E39	CDH13	
473	E40	CDH13	
474	E41	CDH13	
475	E14	CDH13	
476	E42	CDH17	
477	E16	CDH2	
478	E43	CDH2	
479	E70	Cdh2	
480	E15	CDH7	
481	E39	CDK14	
482	E70	Cdk14	
483	E20	CDK18	
484	E70	Cdk18	
485	E14	CDK2	
486	E18	CDK4	
487	E39	CDK5R1	
488	E63	CDK6	
489	E65	CDK6	
490	E10	CDKN1A	
491	E43	CDKN1A	
492	E66	CDKN1A	
493	E67	Cdkn1a	
494	E60	CDKN1A(p21)	
495	E65	CDKN1B	
496	E18	CDKN2A	
497	E46	CDKN2A/B	
498	E63	CDKN2C	
499	E2	CDO1	
500	E46	CDX2	
501	E64	CEACAM5	
502	E11	CEBPD	
503	E33	CEBPD	
504	E14	CECR5	
505	E31	CELF1	
506	E56	CELF6	
507	E45	Cend1	
508	E2	CENPF	
509	E63	CENPF	
510	E36	CENPH	
511	E31	CEP126	
512	E2	CEP192	
513	E58	CEP44	
514	E14	CEP68	

515	E43	CES1	
516	E54	CES1	
517	E59	Ces2g	
518	E35	CFLAR	
519	E11	CFR	
520	E52	CGA	
521	E43	CGB1	
522	E43	CGB5	
523	E52	CHAC1	
524	E58	CHDC2	
525	E60	CHECK1	
526	E17	CHGA	
527	E17	CHGB	
528	E54	CHGB	
529	E56	CHODL	
530	E52	CHRNA2	
531	E17	CHRNA3	
532	E58	CHRNA5	
533	E14	CHRNA9	
534	E27	CHST11	
535	E56	CHST15	
536	E70	Cib1	
537	E69	Cidea	
538	E54	CIITA	
539	E59	Cish	
540	E2	CIT	
541	E26	Cit	
542	E25	CITED1	
543	E70	Cited2	
544	E2	CKAP2	
545	E16	CKAP4	
546	E33	CKB	
547	E51	CKB	
548	E42	CKMT1A	
549	E44	CKMT2	
550	E61	CLASPIN	
551	E41	CLCF1	
552	E56	CLCF1	
553	E21	CLCN7	
554	E26	Cldn11	
555	E7	CLDN3	
556	E13	CLDN4	
557	E59	Clec12a	

558	E11	CLEC4D	
559	E58	CLEC4E	
560	E59	Clec7a	
561	E37	CLECSF2	
562	E26	Clic6	
563	E66	CLIP3	
564	E14	CLN6	
565	E4	CLN8	
566	E31	CLOCK	
567	E62	CLSTN1	
568	E45	Clu	
569	E29	CMBL	
570	E67	Cmklr1	
571	E11	CMTM8	
572	E56	CMTM8	
573	E51	CNGA3	
574	E1	CNN1	
575	E21	CNN2	
576	E70	Cnn2	
577	E2	CNN3	
578	E16	CNN3	
579	E18	CNOT2	
580	E26	Cnp	
581	E35	CNTF	
582	E5	CNTN1	
583	E40	CNTNAP3B	
584	E70	Cobl	
585	E37	COBLL1	
586	E26	Col11a1	
587	E26	Col11a2	
588	E39	COL13A1	
589	E41	COL13A1	
590	E70	Col14a1	
591	E1	COL16A1	
592	E40	COL17A1	
593	E33	COL18A1	
594	E70	Col18a1	
595	E26	Col19a2	
596	E43	COL22A1	
597	E1	COL23A1	
598	E58	COL26A1	
599	E24	COL2A1	
600	E26	Col2a1	

601	E43	COL4A1	
602	E57	COL4A1	
603	E70	Col4a1	
604	E70	Col4a2	
605	E63	COL4A3	
606	E21	COL4A3BP	
607	E58	COL4A4	
608	E40	COL4A6	
609	E43	COL5A1	
610	E3	COL5A2	
611	E33	COL6A1	
612	E60	COL6A1	
613	E33	COL6A2	
614	E14	COL8A1	
615	E26	Col9a1	
616	E1	COL9A2	
617	E58	COLCA1	
618	E31	COP1	
619	E70	Coro1c	
620	E11	COTL1	
621	E61	COX4L1	
622	E61	COX7B	
623	E61	COX7C	
624	E59	Cp	
625	E29	CP	
626	E20	CPA2	
627	E11	CPA3	
628	E31	CPD	
629	E33	CPE	
630	E26	Cplx2	
631	E18	CPM	
632	E2	CPN1	
633	E20	CPS1	
634	E23	CPS1	
635	E11	CR1	
636	E45	Creg1	
637	E34	CRHR1	
638	E20	CRIM1	
639	E41	CRIP2	
640	E54	CRISPLD2	
641	E37	CRY1	
642	E23	CSAG2	
643	E1	CSDC2	

644	E33	CSDE1	
645	E67	Csf1	
646	E38	CSF2	
647	E49	Csf2	
648	E69	Csf2	
649	E23	CSF2	
650	E11	CSF2RB	
651	E38	CSF3	
652	E70	Csk	
653	E40	CSMD2	
654	E15	CSNK1A1L	
655	E15	CSNK2A	
656	E24	CSPG4	
657	E51	CSPG5	
658	E26	Csrp1	
659	E43	CSRP1	
660	E41	CSRP2	
661	E33	CST3	
662	E38	CTBP1	
663	E58	CTD-2114J12.1	
664	E65	CTD-2192J16.22	
665	E25	CTD-2303H24.2	
666	E66	CTDNEP1	
667	E18	CTDSP2	
668	E20	CTGF	
669	E43	CTGF	
670	E70	Ctgf	
671	E17	CTNNA2	
672	E70	Ctnna2	
673	E13	CTNNB1	
674	E50	CTNNB1	
675	E37	CTPS	
676	E21	CTSA	
677	E37	CTSB	
678	E42	CTSE	
679	E11	CTSH	
680	E49	Ctss	
681	E43	CTXN1	
682	E65	CUX2	
683	E21	CX3CL1	
684	E17	CXADR	
685	E58	CXADR	

686	E66	CXADR	
687	E8	CXCL1	
688	E38	CXCL1	
689	E49	Cxcl10	
690	E67	Cxcl10	
691	E70	Cxcl10	
692	E8	CXCL2	
693	E20	CXCL8	
694	E38	CXCR1	
695	E32	CYB5A	
696	E68	CYB5A	
697	E41	CYB5R2	
698	E13	CYBA	
699	E70	Cyba	
700	E47	CYBB	
701	E38	CYGB	
702	E11	CYorf15A	
703	E32	CYP11B1	
704	E32	CYP11B2	
705	E32	CYP17A1	
706	E29	CYP1B1	
707	E57	CYP1B1	
708	E18	CYP27B1	
709	E38	CYP27B1	
710	E42	CYP2C18	
711	E58	CYP2C18	
712	E58	CYP2C19	
713	E45	Cyp2c70	
714	E2	CYP2E1	
715	E42	CYP2S1	
716	E47	CYP2U1	
717	E58	CYP4B1	
718	E63	CYP4B1	
719	E66	CYP4F2	
720	E66	CYP4F3	
721	E25	CYPP19A1	
722	E20	CYR61	
723	E41	CYR61	
724	E67	Cyr61	
725	E58	CYS1	
726	E65	DACH2	
727	E70	Dact3	
728	E14	DAP	

729	E14	DAPK1	
730	E58	DAPK1	
731	E65	DAPK1	
732	E2	DAPK2	
733	E70	Dapk3	
734	E17	DBH	
735	E70	Dbn1	
736	E31	DBP	
737	E58	DCAF12L1	
738	E23	DCBLD2	
739	E25	DCC	
740	E28	DCC	
741	E58	DCDC2	
742	E2	DCTD	
743	E63	DDB2	
744	E66	DDB2	
745	E17	DDC	
746	E42	DDC	
747	E54	DDIT3	
748	E33	DDIT4	
749	E52	DDIT4	
750	E54	DDIT4	
751	E26	Ddn	
752	E65	DDR2	
753	E67	Ddx60	
754	E33	DEAF1	
755	E48	DEDD	
756	E29	DEFB1	
757	E31	DENND6A	
758	E16	DFNA5	
759	E30	DGCR5	
760	E38	DHRS2	
761	E11	DHRS3	
762	E59	Diap2	
763	E14	DIAPH2-AS1	
764	E47	DIRAS1	
765	E31	DIXDC1	
766	E41	DKK1	
767	E52	DKK1	
768	E60	DKK1	
769	E1	DKK3	
770	E40	DKK3	
771	E43	DKK3	

772	E40	DLC1	
773	E14	DLG3	
774	E17	DLK1	
775	E40	DLK2	
776	E12	DLL1	
777	E14	DLL3	
778	E56	DLX5	
779	E63	DLX5	
780	E58	DLX6	
781	E58	DLX6-AS1	
782	E37	DMD	
783	E2	DMPK	
784	E65	DMRT2	
785	E65	DMRT3	
786	E65	DMRTA1	
787	E65	DMRTA2	
788	E59	Dnahc8	
789	E58	DNAJC22	
790	E17	DNAJC6	
791	E66	DNHD1	
792	E31	DNLZ	
793	E49	Dnmbp	
794	E69	Dnmbp	
795	E4	DNMT3B	
796	E58	DNMT3B	
797	E41	DOCK5	
798	E55	Dock9	
799	E42	DOK4	
800	E27	DOK5	
801	E42	DPCR1	
802	E28	DPEP1	
803	E20	DPF3	
804	E37	DPP4	
805	E51	DPP6	
806	E4	DPPA4	
807	E62	DPYSL3	
808	E43	DPYSL4	
809	E60	DRAM1	
810	E66	DRAM1	
811	E67	Dram1	
812	E38	DRAS1	
813	E41	DRD2	
814	E27	DSCR8	

815	E13	DST	
816	E2	DTL	
817	E21	DTNA	
818	E58	DUOX2	
819	E38	DUSP10	
820	E67	Dusp2	
821	E4	DUSP4	
822	E8	DUSP4	
823	E8	DUSP5	
824	E67	Dusp5	
825	E59	Dusp6	
826	E69	Dusp6	
827	E52	DUX4L8	
828	E52	DUX4L9	
829	E26	Dynlrb2	
830	E18	DYRK2	
831	E70	Dyrk3	
832	E27	DYSF	
833	E54	DYSF	
834	E56	DYSF	
835	E5	ECHDC2	
836	E1	ECRG4	
837	E26	Ecrg4	
838	E20	ECT2	
839	E63	ECT2	
840	E66	EDA2R	
841	E70	Edn1	
842	E60	EDN2	
843	E28	EDNRB	
844	E17	EEF1A2	
845	E43	EEF1A2	
846	E56	EFCAB4A	
847	E20	EFHB	
848	E57	EFHB	
849	E65	EFHD1	
850	E46	EFNA2/3	
851	E7	EFNB1	
852	E70	Efnb1	
853	E27	EGFEM1P	
854	E24	EGFLAM	
855	E50	EGFR	
856	E51	EGFR	
857	E29	EGLN3	

858	E24	EGR1	
859	E21	EGR1	
860	E21	EGR2	
861	E60	EGR2	
862	E21	EGR3	
863	E2	EHF	
864	E54	EIF4EBP1	
865	E58	ELAPOR1	
866	E17	ELAVL2	
867	E17	ELAVL3	
868	E17	ELAVL4	
869	E29	ELF3	
870	E1	ELF3	
871	E46	ELFN2/3	
872	E38	ELK3	
873	E2	ELL3	
874	E57	ELMOD1	
875	E59	Elovl7	
876	E54	EMID1	
877	E55	Emid1	
878	E59	Emilin2	
879	E41	EML1	
880	E69	Emp1	
881	E20	EMP2	
882	E16	EMP3	
883	E67	Emp3	
884	E15	EMR1	
885	E59	Emr1	
886	E46	EN2	
887	E59	Enah	
888	E26	Enc	
889	E26	Enc1	
890	E2	ENGASE	
891	E54	ENOX1	
892	E24	ENPP1	
893	E26	Enpp2	
894	E1	ENPP2	
895	E58	ENPP3	
896	E11	ENTPD1	
897	E15	ENTPD1	
898	E6	EOMES	
899	E50	EPB41	
900	E1	EPCAM	

901	E8	EPHA2	
902	E64	EPHA2	
903	E70	Epha2	
904	E43	EPHB1	
905	E27	EPHB2	
906	E3	EPHB2	
907	E49	Ephb2	
908	E2	EPHB3	
909	E41	EPHB3	
910	E38	EPN3	
911	E59	Epx	
912	E14	ERAP1	
913	E50	ERBB2	
914	E60	ERBB4	
915	E8	EREG	
916	E49	Ereg	
917	E54	EREG	
918	E69	Ereg	
919	E7	ERF	
920	E40	ERG	
921	E2	ERI2	
922	E26	Ermn	
923	E58	ERN2	
924	E56	ERP27	
925	E21	ERRFI1	
926	E41	ERRFI1	
927	E2	ESAM	
928	E2	ESPL1	
929	E50	ESR1	
930	E58	ESR1	
931	E65	ESR1	
932	E34	ETV1	
933	E49	Etv1	
934	E69	Etv1	
935	E31	ETV4	
936	E69	Etv4	
937	E20	ETV5	
938	E37	ETV5	
939	E49	Etv5	
940	E69	Etv5	
941	E46	EVX1	
942	E27	EXOC6B	
943	E16	EXT1	

944	E58	EYA2	
945	E57	EYA4	
946	E70	Eya4	
947	E23	F2	
948	E44	F2R	
949	E39	F2RL1	
950	E69	F2rl1	
951	E23	F7	
952	E14	FABP5	
953	E29	FABP6	
954	E51	FABP7	
955	E2	FADS1	
956	E8	FAM107B	
957	E50	FAM129B	
958	E26	Fam131a	
959	E17	FAM163A	
960	E2	FAM171A1	
961	E14	FAM180B	
962	E5	FAM181B	
963	E26	Fam183b	
964	E70	Fam20a	
965	E14	FAM3C2	
966	E62	FAM49B	
967	E14	FAM69B	
968	E14	FAM69C	
969	E38	FAM83A	
970	E42	FAM83E	
971	E41	FAM83G	
972	E35	FAM83H	
973	E58	FAR2P1	
974	E40	FAT3	
975	E1	FBLN7	
976	E65	FBN3	
977	E55	Fbx17	
978	E31	FBXL17	
979	E56	FBXL21	
980	E31	FBXL3	
981	E58	FBXO17	
982	E38	FBXO32	
983	E23	FBXO32	
984	E43	FBXO32	
985	E56	FBXO43	
986	E58	FBXO8	

987	E28	FCER2	
988	E59	Fcgr2b	
989	E10	FCGRT	
990	E33	FCGRT	
991	E28	FCRL1	
992	E28	FCRL2	
993	E28	FCRL5	
994	E65	FEV	
995	E41	FEZ1	
996	E67	Ffar2	
997	E38	FFRMT1	
998	E11	FGD5	
999	E68	FGF12	
1000	E4	FGF17	
1001	E46	FGF18	
1002	E46	FGF19	
1003	E4	FGF2	
1004	E39	FGF2	
1005	E60	FGF2	
1006	E46	FGF3	
1007	E65	FGF5	
1008	E4	FGF8	
1009	E46	FGF8	
1010	E21	FGFBP3	
1011	E37	FGFR1	
1012	E5	FGFR3	
1013	E40	FGFR3	
1014	E46	FGFR3	
1015	E51	FGFR3	
1016	E58	FGFR3	
1017	E2	FGFR4	
1018	E18	FGFR4	
1019	E58	FGFR4	
1020	E15	FGFRL1	
1021	E68	FGG	
1022	E37	FGL2	
1023	E67	Fgl2	
1024	E2	FHDC1	
1025	E40	FHL1	
1026	E29	FHL2	
1027	E15	FITM10	
1028	E40	FJX1	
1029	E41	FJX1	

1030	E21	FLCN	
1031	E54	FLG	
1032	E16	FLNA	
1033	E41	FLNB	
1034	E43	FLNC	
1035	E40	FLRT2	
1036	E34	FLRT3	
1037	E41	FLT1	
1038	E54	FLT3	
1039	E56	FLT4	
1040	E26	Fmod	
1041	E1	FMOD	
1042	E16	FN1	
1043	E33	FN1	
1044	E44	FNDC3B	
1045	E15	FOCAD	
1046	E26	Folr1	
1047	E67	Fos	
1048	E41	FOSL1	
1049	E54	FOSL1	
1050	E10	FOSL2	
1051	E16	FOSL2	
1052	E66	FOSL2	
1053	E34	FOXA2	
1054	E35	FOXA2	
1055	E42	FOXA3	
1056	E54	FOXD1	
1057	E19	FOXE1S	
1058	E41	FOXF1	
1059	E4	FOXH1	
1060	E40	FOXI1	
1061	E26	Foxj1	
1062	E54	FOXJ1	
1063	E70	Foxj1	
1064	E65	FOXL2	
1065	E62	FOXM1	
1066	E31	FOXO3	
1067	E8	FOXQ1	
1068	E41	FREM2	
1069	E60	FRMD4A	
1070	E43	FRMD6	
1071	E66	FRMPD2	
1072	E14	FRMPD4	

1073	E18	FRS2	
1074	E20	FSCN1	
1075	E63	FSCN1	
1076	E39	FST	
1077	E43	FST	
1078	E27	FTCDNL1	
1079	E45	Fth1	
1080	E66	FTX	
1081	E1	FUT3	
1082	E20	FUT5	
1083	E37	FUT8	
1084	E63	FUT8	
1085	E31	FUT9	
1086	E5	FXYD1	
1087	E51	FXYD1	
1088	E29	FXYD2	
1089	E43	FXYD5	
1090	E65	FXYD7	
1091	E58	FYB	
1092	E46	FZD10	
1093	E70	Fzd2	
1094	E42	FZD5	
1095	E15	FZD6	
1096	E2	FZD7	
1097	E21	FZD7	
1098	E50	GAB1	
1099	E58	GABARAPL1	
1100	E31	GABBR1	
1101	E65	GABBR1	
1102	E67	Gabbr1	
1103	E15	GABBR2	
1104	E31	GABPA	
1105	E49	Gabra3	
1106	E55	Gabra5	
1107	E51	GABRB1	
1108	E4	GABRB3	
1109	E34	GABRG1	
1110	E4	GABRP	
1111	E58	GABRP	
1112	E54	GADD45A	
1113	E60	GADD45B	
1114	E60	GADD45G	
1115	E17	GAL	

1116	E43	GAL	
1117	E42	GAL3ST1	
1118	E70	Galk1	
1119	E69	Galnt3	
1120	E62	GALNT7	
1121	E17	GAP43	
1122	E14	GAPDHS	
1123	E34	GARS	
1124	E54	GARS	
1125	E51	GAS1	
1126	E63	GAS2L3	
1127	E41	GAS7	
1128	E59	Gas7	
1129	E59	Gata1	
1130	E59	Gata2	
1131	E62	GATA2	
1132	E65	GATA2	
1133	E17	GATA3	
1134	E65	GATA3	
1135	E42	GATA6	
1136	E54	GATA6	
1137	E70	Gbx2	
1138	E11	GCA	
1139	E20	GCLC	
1140	E45	Gclc	
1141	E45	Gclm	
1142	E42	GCNT3	
1143	E45	Gcnt3	
1144	E58	GCNT3	
1145	E17	GDAP1L1	
1146	E59	Gdf10	
1147	E8	GDF15	
1148	E52	GDF15	
1149	E63	GDF15	
1150	E66	GDF15	
1151	E4	GDF3	
1152	E35	GDF3	
1153	E25	GDF6	
1154	E7	GDPD3	
1155	E1	GEM	
1156	E38	GEM	
1157	E59	Gem	
1158	E26	Gfap	

1159	E37	GFI1	
1160	E49	Gfpt2	
1161	E22	GFRA1	
1162	E41	GFRA1	
1163	E70	Gfra3	
1164	E35	GGCX	
1165	E20	GGH	
1166	E63	GGH	
1167	E45	Ggt6	
1168	E58	GGT6	
1169	E54	GGTLC1	
1170	E11	GIMAP1	
1171	E40	GIMAP8	
1172	E2	GINS1	
1173	E70	Gipc1	
1174	E38	GJB2	
1175	E15	GK5	
1176	E2	GKN3	
1177	E31	GLCCI1	
1178	E21	GLDC	
1179	E41	GLI2	
1180	E16	GLIPR1	
1181	E21	GLIPR1	
1182	E43	GLIPR1	
1183	E43	GLIPR2	
1184	E58	GLRX	
1185	E63	GLS	
1186	E45	Glt28d2	
1187	E26	Glul	
1188	E59	Gm14005	
1189	E45	Gm34240	
1190	E26	Gm42418	
1191	E42	GMDS	
1192	E48	GMDS	
1193	E62	GNA12	
1194	E56	GNAO1	
1195	E66	GNAO1	
1196	E4	GNG13	
1197	E17	GNG3	
1198	E17	GNG4	
1199	E23	GNG4	
1200	E26	Gng7	
1201	E11	GNPDA1	

1202	E61	GNPDA1	
1203	E63	GOLGA2B	
1204	E56	GOLGA7B	
1205	E62	GOPC	
1206	E8	GOS2	
1207	E32	GOS2	
1208	E11	GP1BA	
1209	E59	Gp5	
1210	E42	GPA33	
1211	E1	GPC3	
1212	E64	GPD2	
1213	E54	GPM6A	
1214	E26	Gpnmb	
1215	E29	GPNMB	
1216	E49	Gpnmb	
1217	E58	GPR111	
1218	E59	Gpr141	
1219	E31	GPR155	
1220	E28	GPR160	
1221	E41	GPR176	
1222	E31	GPR180	
1223	E20	GPR35	
1224	E42	GPR35	
1225	E14	GPR39	
1226	E60	GPR39	
1227	E56	GPR56	
1228	E11	GPR84	
1229	E23	GPR84	
1230	E15	GPR85	
1231	E54	GPR87	
1232	E26	Gpr88	
1233	E58	GPR97	
1234	E21	GPRC5B	
1235	E28	GPRC5D	
1236	E63	GPT2	
1237	E42	GPX2	
1238	E19	GPX3	
1239	E58	GPX3	
1240	E33	GPX4	
1241	E58	GR1A2	
1242	E54	GRB10	
1243	E59	Grb10	
1244	E58	GRB14	

1245	E50	GRB2	
1246	E2	GRB7	
1247	E43	GREM1	
1248	E17	GRIA2	
1249	E66	GRIA2	
1250	E4	GRIA4	
1251	E34	GRID1	
1252	E34	GRID2	
1253	E39	GRIM2	
1254	E4	GRIN2B	
1255	E12	GRIP1	
1256	E10	GRK5	
1257	E54	GRRP1	
1258	E14	GS1-257G1.1	
1259	E55	Gsn	
1260	E37	GSS	
1261	E45	Gsta3	
1262	E45	Gstm1	
1263	E45	Gstm3	
1264	E31	GTF2I	
1265	E14	GYG2	
1266	E58	GZMA	
1267	E23	GZMB	
1268	E58	GZMB	
1269	E66	H1F0	
1270	E31	H2AFX	
1271	E67	H2-D1	
1272	E67	H2-Dma	
1273	E67	H2-Q4	
1274	E26	Hap1	
1275	E38	HAS2	
1276	E2	HAUS4	
1277	E58	HAVCR1	
1278	E49	Hbegf	
1279	E67	Hbegf	
1280	E69	Hbegf	
1281	E31	HBP1	
1282	E57	HCN1	
1283	E67	HD-K1	
1284	E23	HEPACAM2	
1285	E42	HEPH	
1286	E38	HEPHL1	
1287	E43	HERPUD1	

1288	E31	HES1	
1289	E33	HES1	
1290	E67	Hes1	
1291	E70	Hes1	
1292	E46	HES2/3/5	
1293	E37	HFE	
1294	E42	HHLA2	
1295	E5	HIF3A	
1296	E65	HIF3A	
1297	E29	HILPDA	
1298	E2	HIRIP3	
1299	E66	HIST1H1E	
1300	E43	HIST1H2BD	
1301	E43	HIST1H2BK	
1302	E37	HIST2H2AA	
1303	E43	HIST2H2AA3	
1304	E66	HK2	
1305	E42	HKDC1	
1306	E14	HLA-B	
1307	E14	HLA-C	
1308	E15	HLA-DMA	
1309	E54	HLA-DMB	
1310	E54	HLA-DPA1	
1311	E68	HLA-DQA1	
1312	E68	HLA-DRA	
1313	E4	HMGA2	
1314	E18	HMGA2	
1315	E40	HMGA2	
1316	E54	HMGA2	
1317	E65	HMGA2	
1318	E70	Hmga2	
1319	E66	HMGCS1	
1320	E63	HMMR	
1321	E46	HMX1/2	
1322	E11	HNRPLL	
1323	E58	HOMER2	
1324	E54	HOPX	
1325	E30	HOXA11-AS	
1326	E65	HOXB5	
1327	E70	Hoxb9	
1328	E23	HOXD11	
1329	E23	HOXD13	
1330	E26	Hpcal4	

1331	E56	HPCAL4	
1332	E54	HPN	
1333	E37	HPRT1	
1334	E22	HR	
1335	E41	HRH1	
1336	E5	HRSP12	
1337	E39	HS3ST3A1	
1338	E21	HSD17B14	
1339	E52	HSD17B14	
1340	E42	HSD17B2	
1341	E66	HSD17B7	
1342	E32	HSD3B2	
1343	E21	HSF4	
1344	E66	HSPA5	
1345	E11	HSPA6	
1346	E31	HSPB1	
1347	E70	Hspb1	
1348	E11	HSPC159	
1349	E65	HTR1E	
1350	E58	HTR3A	
1351	E28	HVCN1	
1352	E21	IARS2	
1353	E17	ICA1	
1354	E31	ICA1	
1355	E60	ICAM1	
1356	E67	Icam1	
1357	E67	Icosl	
1358	E59	Id1	
1359	E19	ID4	
1360	E49	Ida	
1361	E45	Idh1	
1362	E53	IDH2	
1363	E66	IDI1	
1364	E8	IER3	
1365	E41	IER3	
1366	E54	IER3	
1367	E60	IER3	
1368	E31	IER5	
1369	E67	Ifi30	
1370	E59	Ifitm1	
1371	E26	Ifitm3	
1372	E2	IFITM3	
1373	E67	Ifitm3	

1374	E7	IGDCC3	
1375	E58	IGDCC3	
1376	E34	IGF1	
1377	E59	Igf1r	
1378	E34	IGF2	
1379	E62	IGF2	
1380	E66	IGF2	
1381	E4	IGF2BP1	
1382	E23	IGF2BP1	
1383	E4	IGF2BP2	
1384	E4	IGF2BP3	
1385	E11	IGF2R	
1386	E34	IGF2R	
1387	E59	Igfals	
1388	E29	IGFBP1	
1389	E70	Igfbp2	
1390	E29	IGFBP3	
1391	E21	IGFBP3	
1392	E38	IGFBP3	
1393	E60	IGFBP3	
1394	E69	Igfbp3	
1395	E19	IGFBP7	
1396	E40	IGFBP7	
1397	E43	IGFBP7	
1398	E60	IGFBP7	
1399	E29	IGKC	
1400	E29	IGLC2	
1401	E14	IGSF11	
1402	E58	IGSF9	
1403	E41	IL11	
1404	E43	IL11	
1405	E54	IL11	
1406	E11	IL15	
1407	E1	IL17B	
1408	E70	Il17rc	
1409	E15	IL18R1	
1410	E11	IL18RAP	
1411	E40	IL1A	
1412	E38	IL1B	
1413	E14	IL1B	
1414	E49	Il1b	
1415	E54	IL1B	
1416	E54	IL1R2	

1417	E10	IL1RAP	
1418	E15	IL1RAPL1	
1419	E25	IL1RL1	
1420	E49	Il1rl2	
1421	E58	IL20RA	
1422	E54	IL2RB	
1423	E43	IL32	
1424	E40	IL33	
1425	E49	Il33	
1426	E69	Il33	
1427	E38	IL36G	
1428	E15	IL4	
1429	E28	IL5RA	
1430	E24	IL6R	
1431	E8	IL8	
1432	E43	IL8	
1433	E1	ILDR1	
1434	E2	ILF3	
1435	E37	ILK	
1436	E2	IMPDH2	
1437	E68	INHA	
1438	E38	INHBA	
1439	E54	INHBE	
1440	E66	INPP5D	
1441	E66	INSIG1	
1442	E25	INSL3	
1443	E23	INSLA4	
1444	E12	INSM1	
1445	E65	IPO11	
1446	E1	IRAG1	
1447	E33	IRF1	
1448	E7	IRF2	
1449	E60	IRF5	
1450	E49	Irf8	
1451	E67	Irf8	
1452	E69	Irf8	
1453	E35	IRS2	
1454	E65	IRX2	
1455	E31	IRX3	
1456	E67	Isg15	
1457	E67	Isg20	
1458	E17	ISL1	
1459	E65	ISL1	

1460	E25	ISM1	
1461	E20	ITCB2	
1462	E33	ITGA10	
1463	E8	ITGA2	
1464	E15	ITGA2	
1465	E39	ITGA2	
1466	E41	ITGA2	
1467	E49	Itga2	
1468	E64	ITGA2	
1469	E69	Itga2	
1470	E59	Itga2b	
1471	E64	ITGA2B	
1472	E70	Itga3	
1473	E70	Itga4	
1474	E43	ITGA5	
1475	E21	ITGA7	
1476	E28	ITGA8	
1477	E59	Itga9	
1478	E58	ITGAD	
1479	E34	ITGAM	
1480	E59	Itgam	
1481	E24	ITGB1	
1482	E13	ITGB1	
1483	E49	Itgb2	
1484	E38	ITGB3	
1485	E67	Itgb3	
1486	E33	ITGB4	
1487	E64	ITGB6	
1488	E41	ITGB8	
1489	E39	IVNS1ABP	
1490	E42	IYD	
1491	E38	JAG1	
1492	E40	JAG2	
1493	E50	JAK2	
1494	E1	JAM2	
1495	E70	Jam2	
1496	E40	JAM3	
1497	E56	JPH2	
1498	E17	JPH4	
1499	E33	JUNB	
1500	E67	Junb	
1501	E38	KAL1	
1502	E5	KAT2B	

1503	E27	KAZN	
1504	E6	KCNA1	
1505	E12	KCNA1	
1506	E34	KCNA1	
1507	E47	KCNA3	
1508	E58	KCNA5	
1509	E21	KCNAB2	
1510	E62	KCNAB2	
1511	E12	KCNB2	
1512	E26	Kcnc2	
1513	E34	KCNC3	
1514	E58	KCNC3	
1515	E65	KCND1	
1516	E50	KCND3	
1517	E26	Kcne2	
1518	E2	KCNE3	
1519	E10	KCNE3	
1520	E42	KCNE3	
1521	E27	KCNE4	
1522	E7	KCNF1	
1523	E56	KCNF1	
1524	E37	KCNG1	
1525	E43	KCNG1	
1526	E58	KCNG1	
1527	E20	KCNG2	
1528	E12	KCNG3	
1529	E9	KCNH1	
1530	E70	Kcnh2	
1531	E58	KCNH3	
1532	E45	Kcnh7	
1533	E12	KCNH8	
1534	E58	KCNH8	
1535	E51	KCNIP1	
1536	E65	KCNIP3	
1537	E5	KCNJ10	
1538	E31	KCNJ10	
1539	E26	Kcnj13	
1540	E54	KCNJ13	
1541	E19	KCNJ16	
1542	E51	KCNJ16	
1543	E57	KCNJ16	
1544	E58	KCNJ16	
1545	E32	KCNJ5	

1546	E31	KCNJ8	
1547	E48	KCNJ9	
1548	E3	KCNK1	
1549	E8	KCNK1	
1550	E35	KCNK1	
1551	E61	KCNK1	
1552	E23	KCNK12	
1553	E63	KCNK12	
1554	E9	KCNK15	
1555	E22	KCNK15	
1556	E52	KCNK15	
1557	E24	KCNK2	
1558	E68	KCNK2	
1559	E16	KCNK3	
1560	E22	KCNK5	
1561	E11	KCNK6	
1562	E43	KCNK6	
1563	E13	KCNMA1	
1564	E38	KCNMA1	
1565	E39	KCNMA1	
1566	E40	KCNMA1	
1567	E41	KCNMA1	
1568	E60	KCNMA1	
1569	E25	KCNMB1	
1570	E1	KCNMB1	
1571	E12	KCNMB2	
1572	E34	KCNMB2	
1573	E67	Kcnmb2	
1574	E27	KCNMB2-AS1	
1575	E44	KCNMB3	
1576	E18	KCNMB4	
1577	E58	KCNMB4	
1578	E51	KCNN2	
1579	E28	KCNN3	
1580	E4	KCNN3	
1581	E5	KCNN3	
1582	E34	KCNN3	
1583	E8	KCNN4	
1584	E36	KCNN4	
1585	E41	KCNN4	
1586	E49	Kcnn4	
1587	E52	KCNN4	
1588	E53	KCNN4	

1589	E58	KCNN4	
1590	E64	KCNN4	
1591	E69	Kcnn4	
1592	E2	KCNQ1	
1593	E55	Kcnq1	
1594	E58	KCNQ1	
1595	E29	KCNQ1OT1	
1596	E33	KCNQ1OT1	
1597	E66	KCNQ1OT1	
1598	E17	KCNQ2	
1599	E46	KCNQ2	
1600	E59	Kcnq3	
1601	E14	KCNQ5	
1602	E17	KCNQ5	
1603	E40	KCNQ5	
1604	E30	KCNQ5-IT1	
1605	E34	KCNS1	
1606	E34	KCNS3	
1607	E34	KCNT1	
1608	E15	KCNT2	
1609	E23	KCNU1	
1610	E46	KDM2B	
1611	E30	KDM4A-AS1	
1612	E66	KDM6B	
1613	E67	Kdm6b	
1614	E6	KHBRBS2	
1615	E38	KIAA1644	
1616	E2	KIF12	
1617	E2	KIF20A	
1618	E17	KIF21A	
1619	E65	KIF21B	
1620	E14	KIF3B	
1621	E69	Kif5c	
1622	E40	KIRREL	
1623	E34	KISS1	
1624	E59	Kit	
1625	E26	Kl	
1626	E59	Klf1	
1627	E33	KLF10	
1628	E67	Klf10	
1629	E31	KLF12	
1630	E66	KLF16	
1631	E69	Klf4	

1632	E67	Klf6	
1633	E17	KLHL13	
1634	E21	KLHL24	
1635	E21	KLHL4	
1636	E52	KLK13	
1637	E23	KLK14	
1638	E37	KLK2	
1639	E15	KLRK1	
1640	E10	KMO	
1641	E64	KRAS	
1642	E69	Kras	
1643	E22	KRT15	
1644	E60	KRT17	
1645	E45	Krt18	
1646	E24	KRT19	
1647	E29	KRT19	
1648	E22	KRT19	
1649	E60	KRT19	
1650	E45	Krt222	
1651	E58	KRT23	
1652	E7	KRT5	
1653	E54	KRT6A	
1654	E54	KRT6B	
1655	E38	KRT76	
1656	E45	Krt8	
1657	E38	KRTAP19	
1658	E70	L1cam	
1659	E58	LAMA1	
1660	E57	LAMA2	
1661	E56	LAMA4	
1662	E33	LAMA5	
1663	E33	LAMB2	
1664	E41	LAMB3	
1665	E54	LAMC2	
1666	E20	LAMP5	
1667	E37	LASS6	
1668	E59	Lass6	
1669	E28	LAX1	
1670	E54	LBH	
1671	E26	Lbp	
1672	E31	LBR	
1673	E53	LCK	
1674	E65	LCN12	

1675	E21	LCNL1	
1676	E2	LCP1	
1677	E43	LCP1	
1678	E29	LDHA	
1679	E55	Ldha	
1680	E66	LDLR	
1681	E67	Ldlr	
1682	E37	LDOC1	
1683	E2	LECT2	
1684	E32	LEF1	
1685	E55	Lef1	
1686	E68	LEF1	
1687	E4	LEFTY1	
1688	E4	LEFTY2	
1689	E27	LEKR1	
1690	E34	LEP	
1691	E34	LEPR	
1692	E31	LFNG	
1693	E26	Lgals1	
1694	E16	LGALS1	
1695	E29	LGALS3	
1696	E59	Lgals3	
1697	E42	LGALS4	
1698	E51	LGI1	
1699	E2	LGR5	
1700	E36	LGR5	
1701	E57	LGR6	
1702	E25	LHCGR	
1703	E70	Lhfp	
1704	E59	Lhfpl2	
1705	E46	LHX3	
1706	E65	LHX3	
1707	E46	LHX3/4	
1708	E49	Lif	
1709	E54	LIF	
1710	E60	LIF	
1711	E67	Lif	
1712	E69	Lif	
1713	E8	LIF	
1714	E1	LIFR	
1715	E15	LIMK2	
1716	E1	LIMS2	
1717	E4	LIN28A	

1718	E65	LIN28B	
1719	E14	LINC00282	
1720	E14	LINC00348	
1721	E30	LINC00511	
1722	E25	LINC00607	
1723	E29	LINC00626	
1724	E27	LINC00886	
1725	E66	LINC01021	
1726	E25	LINC01116	
1727	E58	LINC01132	
1728	E29	LINC01320	
1729	E14	LINC01531	
1730	E58	LINC01671	
1731	E58	LINC02532	
1732	E52	LINC02683	
1733	E26	Lingo1	
1734	E57	LINGO2	
1735	E21	LIPG	
1736	E1	LIPH	
1737	E2	LIPT2	
1738	E70	Llgl2	
1739	E5	LMO3	
1740	E51	LMO3	
1741	E1	LMOD1	
1742	E65	LMX1A	
1743	E43	LOC100132564	
1744	E23	LOC100190940	
1745	E24	LOC100192378	
1746	E66	LOC729966	
1747	E17	LONRF2	
1748	E16	LOXL2	
1749	E39	LOXL2	
1750	E41	LOXL2	
1751	E65	LPA	
1752	E34	LPAR3	
1753	E21	LPCAT1	
1754	E31	LPGAT1	
1755	E37	LPL	
1756	E43	LPXN	
1757	E11	LRG1	
1758	E2	LRIG1	
1759	E15	LRP11	
1760	E21	LRP4	

1761	E14	LRPPRC	
1762	E41	LRRC15	
1763	E31	LRRC4	
1764	E70	Lrrk1	
1765	E56	LRRN2	
1766	E17	LRRN3	
1767	E52	LRRTM1	
1768	E58	LRRTM1	
1769	E17	LRRTM2	
1770	E28	LSAMP	
1771	E28	LSR	
1772	E21	LSS	
1773	E54	LTB	
1774	E38	LTB	
1775	E60	LTBP1	
1776	E40	LTBP2	
1777	E70	Ltbp2	
1778	E33	LTBP3	
1779	E24	LUM	
1780	E45	Ly6a	
1781	E7	LY6D	
1782	E33	LY6E	
1783	E50	LYN	
1784	E42	LYZ	
1785	E54	LZTFL1	
1786	E66	LZTS1	
1787	E17	MAB21L2	
1788	E26	Mag	
1789	E26	Mal	
1790	E13	MAL2	
1791	E57	MAL2	
1792	E69	Mall	
1793	E14	MAMDC2-AS1	
1794	E66	MAMDC4	
1795	E11	MANEAL	
1796	E17	MAOA	
1797	E1	MAOB	
1798	E44	MAP1B	
1799	E43	MAP1LC3A	
1800	E60	MAP2K6	
1801	E15	MAP3K14	
1802	E58	MAP3K15	
1803	E56	MAP3K5	

1804	E64	MAP3K5	
1805	E64	MAP3K6	
1806	E16	MAP4K4	
1807	E14	MAP6D1	
1808	E50	MAPK1	
1809	E59	Mapk13	
1810	E50	MAPK14	
1811	E38	MAPK6	
1812	E21	MAPT	
1813	E56	MARCH4	
1814	E16	MARCKS	
1815	E54	MARK1	
1816	E14	MARS2	
1817	E2	MARVELD1	
1818	E27	MAST4	
1819	E70	Matn2	
1820	E31	MAZ	
1821	E1	MB	
1822	E14	MBD5	
1823	E59	Mc5r	
1824	E70	Mcam	
1825	E62	MCFD2	
1826	E26	Mcm2	
1827	E60	MCM2	
1828	E2	MCM8	
1829	E68	MCOLN3	
1830	E59	Mcpt8	
1831	E59	Mdga1	
1832	E48	MDH1	
1833	E18	MDM2	
1834	E2	MECOM	
1835	E26	Mef2c	
1836	E1	MEF2C	
1837	E59	Mefv	
1838	E1	MEG3	
1839	E26	Melat1	
1840	E52	MEP1A	
1841	E14	MESDC1	
1842	E7	MESP1	
1843	E23	MET	
1844	E59	Met	
1845	E62	MET	
1846	E67	Met	

1847	E18	METTL1	
1848	E18	METTL21B	
1849	E66	METTL7A	
1850	E21	METTL7B	
1851	E21	MFGE8	
1852	E58	MFSD2A	
1853	E59	Mfsd2b	
1854	E14	MGAT4C	
1855	E26	Mia	
1856	E22	MICB	
1857	E2	MIF	
1858	E30	MIR205HG	
1859	E30	MIR31HG	
1860	E65	MKX	
1861	E51	MLC1	
1862	E25	MLK4	
1863	E23	MLLT11	
1864	E1	MLPH	
1865	E11	MLT11	
1866	E69	Mmd	
1867	E29	MME	
1868	E44	MMP1	
1869	E56	MMP1	
1870	E64	MMP1	
1871	E38	MMP1	
1872	E38	MMP10	
1873	E44	MMP10	
1874	E49	Mmp10	
1875	E24	MMP14	
1876	E40	MMP3	
1877	E54	MMP7	
1878	E58	MMP7	
1879	E38	MMP9	
1880	E59	Mmrn1	
1881	E65	MNX1	
1882	E46	MNX1	
1883	E30	MNX1-AS1	
1884	E58	MOB1B	
1885	E26	Mobp	
1886	E26	Mog	
1887	E31	MORC3	
1888	E11	MOSC2	
1889	E57	MOXD1	

1890	E13	MPDZ	
1891	E59	Mpl	
1892	E2	MPP3	
1893	E2	MPZL1	
1894	E3	MPZL1	
1895	E15	MPZL2	
1896	E69	Mpzl2	
1897	E21	MRC2	
1898	E40	MRC2	
1899	E14	MREG	
1900	E24	MRGPRX3	
1901	E14	MRPL24	
1902	E7	MRPL4	
1903	E31	MRPS33	
1904	E45	Mrps35	
1905	E28	MS4A1	
1906	E58	MS4A7	
1907	E64	MSLN	
1908	E66	MSMO1	
1909	E21	MSN	
1910	E43	MSN	
1911	E63	MSN	
1912	E15	MSR1	
1913	E40	MSRB3	
1914	E43	MT1A	
1915	E25	MT1F	
1916	E43	MT1X	
1917	E26	Mt2	
1918	E43	MT2A	
1919	E21	MT-CYB	
1920	E63	MTHFD2	
1921	E52	MTND4P12	
1922	E58	MTRNR2L12	
1923	E1	MUC1	
1924	E7	MUC1	
1925	E42	MUC13	
1926	E42	MUC17	
1927	E41	MUC5B	
1928	E58	MUC6	
1929	E40	MUM1L1	
1930	E21	MVD	
1931	E23	MYBL1	
1932	E14	MYC	

1933	E35	MYC	
1934	E61	MYC	
1935	E17	MYCN	
1936	E25	MYH11	
1937	E1	MYH11	
1938	E38	MYH16	
1939	E21	MYH9	
1940	E70	Myh9	
1941	E1	MYL9	
1942	E1	MYLK	
1943	E65	MYO18B	
1944	E42	MYO1A	
1945	E2	MYO1B	
1946	E59	Myo1d	
1947	E1	MYO5B	
1948	E1	MYO5C	
1949	E42	MYO7B	
1950	E21	MYRF	
1951	E18	MYRFL	
1952	E17	MYT2L	
1953	E21	NAB2	
1954	E4	NANOG	
1955	E49	Nap1l2	
1956	E26	Nap1l5	
1957	E2	NAPEPLD	
1958	E33	NARF	
1959	E14	NAV2	
1960	E12	NCAM1	
1961	E68	NCAM1	
1962	E66	NCAM-AS1	
1963	E5	NCAN	
1964	E17	NCAN	
1965	E51	NCAN	
1966	E14	NCL	
1967	E14	NCOA3	
1968	E26	Ndn	
1969	E8	NDRG1	
1970	E63	NDRG1	
1971	E26	Ndrg2	
1972	E38	NDRG4	
1973	E51	NDRG4	
1974	E29	NDUFA4L2	
1975	E66	NDUFB9	

1976	E33	NEAT1	
1977	E56	NEBL	
1978	E37	NEDD9	
1979	E34	NEFH	
1980	E17	NEFL	
1981	E17	NEFM	
1982	E41	NEGR1	
1983	E25	NELL2	
1984	E57	NELL2	
1985	E15	NEO1	
1986	E60	NEO1	
1987	E51	NES	
1988	E21	NEU1	
1989	E65	NEUROD1	
1990	E65	NEUROD4	
1991	E34	NF1	
1992	E35	NF1B	
1993	E24	NFAT5	
1994	E31	NFAT5	
1995	E2	NFIA	
1996	E31	NFIA	
1997	E58	NFIA	
1998	E12	NFIB	
1999	E58	NFIB	
2000	E33	NFIC	
2001	E66	NFIC	
2002	E66	NFIX	
2003	E50	NFKB1	
2004	E8	NFKBIZ	
2005	E31	NFKBIZ	
2006	E41	NGEF	
2007	E49	Ngf	
2008	E1	NGFR	
2009	E40	NGFR	
2010	E62	NGRN	
2011	E31	NHEJ1	
2012	E58	NID2	
2013	E60	NID2	
2014	E15	NINJ1	
2015	E25	NIPAL1	
2016	E56	NIPAL1	
2017	E40	NIPAL4	
2018	E23	NKG7	

2019	E19	NKX2-1	
2020	E35	NKX2-1	
2021	E65	NKX2-2	
2022	E52	NKX3-1	
2023	E46	NKX6-2	
2024	E4	NLGN4X	
2025	E4	NLGN4Y	
2026	E43	NLRP1	
2027	E67	Nlrp3	
2028	E29	NMB	
2029	E17	NMNAT2	
2030	E41	NMNAT2	
2031	E34	NMU	
2032	E60	NMU	
2033	E56	NNAT	
2034	E29	NNMT	
2035	E2	NOD1	
2036	E4	NODAL	
2037	E70	Nog	
2038	E14	NOP56	
2039	E2	NOTCH1	
2040	E60	NOTCH1	
2041	E66	NOTCH1	
2042	E66	NOTCH3	
2043	E40	NOTCH4	
2044	E59	Nov	
2045	E70	Nov	
2046	E66	NOVA2	
2047	E21	NPC2	
2048	E65	NPHS1	
2049	E34	NPPA	
2050	E70	Nppb	
2051	E43	NPTX1	
2052	E65	NPTX1	
2053	E26	Nptxr	
2054	E19	NPY1R	
2055	E34	NPY1R	
2056	E34	NPY2R	
2057	E65	NR0B1	
2058	E69	Nr1h4	
2059	E51	NR2E1	
2060	E2	NR2E3	
2061	E33	NR2F1	

2062	E7	NR4A1	
2063	E67	Nr4a2	
2064	E67	Nr4a3	
2065	E65	NR5A1	
2066	E62	NRBF2	
2067	E31	NRCAM	
2068	E44	NREP	
2069	E40	NRG1	
2070	E59	Nrg1	
2071	E54	NRG2	
2072	E26	Nrgn	
2073	E58	NRGN	
2074	E42	NRIL2	
2075	E26	Nrip3	
2076	E10	NROB1	
2077	E39	NRP1	
2078	E41	NRP1	
2079	E14	NRP1	
2080	E21	NRP2	
2081	E17	NRSN1	
2082	E41	NRXN3	
2083	E60	NT5E	
2084	E34	NTF3	
2085	E25	NTM	
2086	E39	NTM	
2087	E41	NTN4	
2088	E46	NTNG2	
2089	E46	NTSR1	
2090	E51	NTSR2	
2091	E70	Nuak1	
2092	E70	Nuak2	
2093	E34	NUCB1	
2094	E33	NUCB2	
2095	E34	NUCB2	
2096	E37	NUDT1	
2097	E45	Nupr1	
2098	E60	ODC1	
2099	E58	OGDHL	
2100	E26	Olfm1	
2101	E22	OLFM1	
2102	E58	OLFM2	
2103	E54	OPN1SW	
2104	E41	OPRD1	

2105	E66	OPRM1	
2106	E16	OPTN	
2107	E15	OR4K2	
2108	E1	ORAI1	
2109	E2	ORC1	
2110	E39	OSMR	
2111	E1	OSR1	
2112	E20	OSR1	
2113	E46	OTX2	
2114	E11	OVOS2	
2115	E26	Oxt	
2116	E37	P2RX1	
2117	E28	P2RX5	
2118	E67	P2ry2	
2119	E55	P4ha2	
2120	E47	PAG1	
2121	E68	PAH	
2122	E2	PAICS	
2123	E37	PAK1	
2124	E62	PAK4	
2125	E2	PALB2	
2126	E21	PALLD	
2127	E70	Palld	
2128	E54	PALM	
2129	E43	PAPPA	
2130	E11	PAPSS2	
2131	E68	PAPSS2	
2132	E42	PAQR8	
2133	E28	PARM1	
2134	E43	PAT1	
2135	E46	PAX2	
2136	E51	PAX6	
2137	E4	PAX7	
2138	E29	PAX8	
2139	E31	PBX1	
2140	E66	PCBP2	
2141	E60	PCCA	
2142	E41	PCDH1	
2143	E21	PCDH1	
2144	E65	PCDH17	
2145	E1	PCDH18	
2146	E31	PCDH9	
2147	E66	PCDHGC3	

2148	E60	PCNA	
2149	E68	PCP4	
2150	E23	PCSK1	
2151	E49	Pcsk1n	
2152	E66	PCSK1N	
2153	E26	Pde10a	
2154	E55	Pde11a	
2155	E58	PDE11A	
2156	E26	Pde1b	
2157	E38	PDE2A	
2158	E65	PDE3A	
2159	E2	PDE3B	
2160	E67	Pde4b	
2161	E1	PDGFA	
2162	E21	PDGFA	
2163	E70	Pdgfa	
2164	E21	PDGFB	
2165	E70	Pdgfb	
2166	E39	PDGFC	
2167	E43	PDGFC	
2168	E70	Pdgfrl	
2169	E26	Pdp1	
2170	E50	PDP1	
2171	E40	PDPN	
2172	E60	PDRG1	
2173	E29	PDZK1IP1	
2174	E16	PEA15	
2175	E31	PEA15	
2176	E68	PEG10	
2177	E49	Peg3	
2178	E26	Penk	
2179	E67	Per1	
2180	E28	PERP	
2181	E43	PFKFB4	
2182	E16	PFKP	
2183	E37	PFKP	
2184	E44	PFN2	
2185	E58	PGBD5	
2186	E54	PGC	
2187	E11	PGCP	
2188	E66	PGF	
2189	E58	PGR	
2190	E17	PHACTR3	

2191	E63	PHGDH	
2192	E66	PHGDH	
2193	E10	PHLDA1	
2194	E45	Phlda1	
2195	E54	PHLDA1	
2196	E8	PHLDA2	
2197	E41	PHLDA2	
2198	E17	PHOX2A	
2199	E17	PHOX2B	
2200	E58	PI3	
2201	E2	PICALM	
2202	E66	PIDD	
2203	E34	PIEZO2	
2204	E49	Pigr	
2205	E50	PIK3C2B	
2206	E62	PIP4K2A	
2207	E52	PIP5K1B	
2208	E58	PIP5K1B	
2209	E65	PITX2	
2210	E1	PKD1	
2211	E65	PKD1L1	
2212	E21	PKDCC	
2213	E58	PKHD1	
2214	E55	Pkp4	
2215	E9	PLA2G10	
2216	E20	PLA2G4E	
2217	E11	PLAG1	
2218	E57	PLAG1	
2219	E65	PLAGL1	
2220	E33	PLAT	
2221	E64	PLAT	
2222	E69	Plat	
2223	E49	Plau	
2224	E54	PLAU	
2225	E64	PLAU	
2226	E67	Plau	
2227	E38	PLAU	
2228	E8	PLAUR	
2229	E13	PLAUR	
2230	E41	PLAUR	
2231	E43	PLAUR	
2232	E49	Plaur	
2233	E69	Plaur	

2234	E2	PLCE1	
2235	E47	PLCL2	
2236	E52	PLCXD3	
2237	E69	Plek2	
2238	E29	PLEKHA1	
2239	E54	PLEKHA4	
2240	E68	PLEKHA5	
2241	E14	PLEKHS1	
2242	E58	PLEKHS1	
2243	E67	Plk2	
2244	E16	PLOD2	
2245	E26	Plp1	
2246	E65	PLP1	
2247	E1	PLPP1	
2248	E28	PLPP5	
2249	E67	Plscr1	
2250	E31	PLTP	
2251	E70	Plxnbp2	
2252	E39	PLXNC1	
2253	E25	PMAIP1	
2254	E60	PMAIP1(NOXA)	
2255	E26	Pmch	
2256	E41	PMEPA1	
2257	E43	PMEPA1	
2258	E16	PML	
2259	E35	PNMA2	
2260	E51	PNOC	
2261	E67	Pnp	
2262	E31	PODXL	
2263	E42	POF18	
2264	E2	POLD1	
2265	E2	POLE	
2266	E2	POLI	
2267	E66	POLR2A	
2268	E44	POLR2H	
2269	E62	POM121	
2270	E23	POPDC3	
2271	E1	POSTN	
2272	E19	POSTN	
2273	E70	Postn	
2274	E65	POU1F1	
2275	E38	POU2F2	
2276	E31	POU3F2	

2277	E51	POU3F2	
2278	E19	POU3F3	
2279	E31	POU3F3	
2280	E4	POU5F1	
2281	E50	PPARG	
2282	E2	PPAT	
2283	E49	Ppbp	
2284	E13	PPL	
2285	E5	PPMiK	
2286	E38	PPP1CB	
2287	E1	PPP1R14A	
2288	E31	PPP1R14C	
2289	E49	Ppp1R15a	
2290	E67	Ppp1r15a	
2291	E26	Ppp1r1b	
2292	E58	PPP1R3B	
2293	E26	Ppp1r9b	
2294	E62	PPP2R1B	
2295	E58	PPP2R2C	
2296	E41	PRDM1	
2297	E27	PRDM16	
2298	E65	PRDM8	
2299	E45	Prdx6	
2300	E2	PRELP	
2301	E41	PRICKLE2	
2302	E60	PRIM1	
2303	E70	Prkaa1	
2304	E58	PRKAG2-AS1	
2305	E47	PRKAR2B	
2306	E24	PRKCB	
2307	E26	Prkcd	
2308	E27	PRKCE	
2309	E70	Prkcq	
2310	E50	PRKD1	
2311	E59	Prkd2	
2312	E49	Prkg2	
2313	E69	Prkg2	
2314	E5	PRODH	
2315	E66	PRODH	
2316	E7	PROM1	
2317	E11	PROS1	
2318	E54	PROS1	
2319	E12	PROX1	

2320	E66	PRR12	
2321	E66	PRR36	
2322	E1	PRRX1	
2323	E41	PRSS23	
2324	E59	Prss34	
2325	E31	PRTG	
2326	E48	PSAT1	
2327	E54	PSAT1	
2328	E63	PSAT1	
2329	E26	Psd	
2330	E5	PSD2	
2331	E67	Psmb9	
2332	E44	PSMD2	
2333	E55	Pstpip2	
2334	E60	PSTPIP2	
2335	E6	PTEN	
2336	E26	Ptgds	
2337	E21	PTGDS	
2338	E67	Ptger4	
2339	E60	PTGES(PIG12)	
2340	E52	PTGFR	
2341	E65	PTGFR	
2342	E49	Ptgs2	
2343	E34	PTH1R	
2344	E58	PTHLH	
2345	E3	PTK7	
2346	E50	PTPN1	
2347	E50	PTPN12	
2348	E50	PTPN21	
2349	E66	PTPN23	
2350	E50	PTPN6	
2351	E13	PTPRC	
2352	E1	PTPRE	
2353	E15	PTPRE	
2354	E67	Ptpre	
2355	E50	PTPRJ	
2356	E27	PTPRK	
2357	E70	Ptprm	
2358	E11	PTPRN2	
2359	E2	PTPRO	
2360	E1	PTPRT	
2361	E31	PTPRZ1	
2362	E36	PTRH1	

2363	E58	PVR	
2364	E66	PVRL4	
2365	E30	PVT1	
2366	E66	PVT1	
2367	E13	PXN	
2368	E35	PXN	
2369	E42	PZDP3	
2370	E11	QPCT	
2371	E21	QPRT	
2372	E1	QRICH2	
2373	E2	QSOX2	
2374	E16	RAB11FIP5	
2375	E35	RAB12	
2376	E17	RAB33A	
2377	E11	RAB34	
2378	E14	RAB38	
2379	E54	RAB3B	
2380	E56	RAB3C	
2381	E16	RAB6IP1	
2382	E35	RAC1	
2383	E61	RAD51	
2384	E26	Ramp3	
2385	E34	RAMP3	
2386	E56	RAMP3	
2387	E44	RAP2B	
2388	E59	Rapgef3	
2389	E47	RAPGEF5	
2390	E22	RAPGEFL1	
2391	E59	Raph1	
2392	E66	RAPH1	
2393	E70	Rarb	
2394	E70	Rarg	
2395	E26	Rarres2	
2396	E29	RARRES2	
2397	E2	RASA3	
2398	E26	Rasd2	
2399	E42	RASEF	
2400	E58	RASGRF1	
2401	E59	Rasgrf2	
2402	E51	RASGRP1	
2403	E2	RASL11B	
2404	E2	RASSF4	
2405	E59	Rassf4	

2406	E66	RAVER1
2407	E46	RAX
2408	E65	RAX2
2409	E22	RBBP8
2410	E58	RBBP8NL
2411	E14	RBFA
2412	E56	RBFOX1
2413	E60	RBL1
2414	E6	RBM24
2415	E17	RBMS3
2416	E58	RBP7
2417	E12	RBPJ
2418	E24	RCAN3
2419	E25	RCAN3
2420	E58	RCAN3
2421	E10	RCOR
2422	E55	Rcvm
2423	E14	RDH13
2424	E21	RDH5
2425	E67	Rel
2426	E34	RELN
2427	E57	RELN
2428	E69	Reln
2429	E21	RENBP
2430	E66	RERE
2431	E26	Resp18
2432	E12	RET
2433	E15	RET
2434	E22	RET
2435	E31	REXO1
2436	E65	RFX4
2437	E33	RGCC
2438	E2	RGMB
2439	E49	Rgs16
2440	E20	RGS17
2441	E60	RGS2
2442	E17	RGS4
2443	E56	RGS7
2444	E26	Rgs9
2445	E14	RGS9
2446	E2	RHOBTB3
2447	E33	RHOBTB3
2448	E13	RHOU

2449	E1	RHPN2	
2450	E31	RIBC1	
2451	E4	RIMS1	
2452	E67	Ripk1	
2453	E64	RIPK3	
2454	E14	RLBP1	
2455	E34	RLN1	
2456	E52	RLN1	
2457	E31	RND2	
2458	E67	Rnf144b	
2459	E21	RNF145	
2460	E2	RNF32	
2461	E58	RNF39	
2462	E66	RNU1-1	
2463	E38	ROBO4	
2464	E41	ROBO4	
2465	E57	ROPN1L	
2466	E65	RORB	
2467	E25	RP1	
2468	E25	RP11-141M1.3	
2469	E65	RP11-192H23.4	
2470	E65	RP11-362F19.1	
2471	E58	RP11-392E22.10	
2472	E58	RP11-392E22.12	
2473	E21	RP11-644F5.10	
2474	E65	RP11-701P16.2	
2475	E14	RP11-79H23.3	
2476	E14	RP13-631K18.2	
2477	E25	RP1-78014.1	
2478	E66	RP3-326I13.1	
2479	E65	RP5-842K16.1	
2480	E51	RPH3A	
2481	E48	RPIA	
2482	E65	RPL21	
2483	E65	RPL37	
2484	E65	RPLP0	
2485	E38	RPLPOP2	
2486	E60	RPRM	
2487	E65	RPS16	
2488	E44	RPS23	
2489	E65	RPS23	

2490	E19	RPS4Y1	
2491	E65	RPS5	
2492	E60	RRAD	
2493	E70	Rras2	
2494	E54	RRM2	
2495	E66	RRM2B	
2496	E24	RRN3P3	
2497	E65	RRS1	
2498	E67	Rsad2	
2499	E26	Rsph1	
2500	E65	RSPO2	
2501	E32	RSPO3	
2502	E66	RTL1	
2503	E11	RTN1	
2504	E17	RTN1	
2505	E21	RTN1	
2506	E44	RTP4	
2507	E3	RUNX1	
2508	E35	RUNX1	
2509	E58	RUNX3	
2510	E34	RXFP1	
2511	E65	RXRG	
2512	E65	RYR1	
2513	E51	RYR3	
2514	E23	S100A2	
2515	E60	S100A2	
2516	E64	S100A2	
2517	E58	S100A3	
2518	E64	S100A4	
2519	E8	S100A6	
2520	E38	S100A6	
2521	E38	S100A9	
2522	E58	S100A9	
2523	E59	S1pr1	
2524	E60	SAA1(PIG4)	
2525	E5	SALLA	
2526	E35	SAMD4A	
2527	E44	SAMD9	
2528	E5	SAMD9L	
2529	E67	Samhd1	
2530	E11	SAMSN1	
2531	E60	SARDH	
2532	E5	SASH1	

2533	E59	Satb1	
2534	E46	SATB2	
2535	E66	SBK1	
2536	E50	SCAMP3	
2537	E60	SCARA3	
2538	E24	SCARA5	
2539	E43	SCARNA13	
2540	E43	SCARNA8	
2541	E61	SCC1	
2542	E45	Scd2	
2543	E35	SCFD2	
2544	E34	SCG2	
2545	E54	SCG2	
2546	E17	SCG5	
2547	E43	SCG5	
2548	E49	Scg5	
2549	E58	SCGB1D2	
2550	E58	SCGB2A1	
2551	E16	SCHIP1	
2552	E52	SCIN	
2553	E59	Scin	
2554	E60	SCL4A10	
2555	E52	SCML4	
2556	E12	SCN3A	
2557	E17	SCN3A	
2558	E26	Scn4b	
2559	E4	SCN5A	
2560	E70	Scn5a	
2561	E34	SCN7A	
2562	E28	SDC1	
2563	E8	SDC4	
2564	E67	Sdc4	
2565	E41	SDK1	
2566	E58	SDK2	
2567	E59	Sdpr	
2568	E70	Sdpr	
2569	E14	SEC24D	
2570	E2	SECTM1B	
2571	E15	SELP	
2572	E54	SELPLG	
2573	E41	SEMA3B	
2574	E44	SEMA3C	
2575	E63	SEMA3C	

2576	E70	Sema3d	
2577	E70	Sema3f	
2578	E59	Sema4a	
2579	E70	Sema4f	
2580	E42	SEMA4G	
2581	E21	SEMA7A	
2582	E70	Sema7a	
2583	E37	SEPT10	
2584	E44	SERP1	
2585	E59	Serpina3f	
2586	E8	SERPINB1	
2587	E40	SERPINB13	
2588	E8	SERPINB5	
2589	E14	SERPINB8	
2590	E54	SERPINB8	
2591	E67	Serpinb8	
2592	E20	SERPINE1	
2593	E60	SERPINE1(PAI1)	
2594	E38	SERPINE2	
2595	E54	SERPINE2	
2596	E57	SERPINE2	
2597	E1	SERPINF1	
2598	E33	SERPINF1	
2599	E40	SERPINF1	
2600	E41	SERTAD4	
2601	E31	SESN3	
2602	E60	SESN3	
2603	E62	SESN3	
2604	E8	SFN	
2605	E60	SFN(14-3-3σ)	
2606	E4	SFRP1	
2607	E41	SFRP1	
2608	E70	Sfrp1	
2609	E54	SFTPD	
2610	E14	SFXN4	
2611	E14	SGCB	
2612	E1	SGIP1	
2613	E21	SGK223	
2614	E70	Sgms1	
2615	E54	SH2D3C	
2616	E40	SH2D5	
2617	E42	SH3BGRL2	
2618	E16	SH3GLB1	

2619	E43	SH3KBP1	
2620	E41	SH3PXD2A	
2621	E66	SH3PXD2B	
2622	E26	Shank1	
2623	E58	SHANK1	
2624	E50	SHC1	
2625	E50	SHC3	
2626	E11	SHCBP1	
2627	E63	SHCBP1	
2628	E1	SHE	
2629	E46	SHH	
2630	E65	SHH	
2631	E70	Shh	
2632	E2	SHISA2	
2633	E41	SHISA2	
2634	E46	SHISA2	
2635	E56	SHISA2	
2636	E63	SHISA4	
2637	E42	SI	
2638	E63	SIAE	
2639	E45	Sidt2	
2640	E20	SIM1	
2641	E46	SIM2	
2642	E45	Simapos2	
2643	E14	SIRPAP1	
2644	E20	SIX1	
2645	E70	Six4	
2646	E66	SKI	
2647	E31	SKIL	
2648	E44	SKIL	
2649	E31	SKIV2L	
2650	E35	SKP2	
2651	E37	SLAMF1	
2652	E28	SLAMF7	
2653	E21	SLC04A1	
2654	E66	SLC12A4	
2655	E31	SLC12A6	
2656	E41	SLC14A1	
2657	E14	SLC14A1	
2658	E58	SLC14A1	
2659	E59	Slc16a11	
2660	E8	SLC16A3	
2661	E43	SLC16A3	

2662	E9	SLC16A6	
2663	E26	Slc17a6	
2664	E26	Slc17a7	
2665	E56	SLC18A1	
2666	E4	SLC18A2	
2667	E59	Slc18a2	
2668	E56	SLC18A3	
2669	E14	SLC19A2	
2670	E58	SLC1A1	
2671	E26	Slc1a2	
2672	E51	SLC1A2	
2673	E58	SLC1A2	
2674	E67	Slc1a2	
2675	E51	SLC1A3	
2676	E58	SLC1A3	
2677	E28	SLC1A4	
2678	E39	SLC22A4	
2679	E51	SLC24A3	
2680	E38	SLC25A24	
2681	E27	SLC25A26	
2682	E14	SLC25A42	
2683	E19	SLC26A4	
2684	E2	SLC27A2	
2685	E14	SLC27A3	
2686	E1	SLC28A3	
2687	E43	SLC2A3	
2688	E3	SLC2A5	
2689	E56	SLC30A3	
2690	E67	Slc31a2	
2691	E18	SLC35E3	
2692	E41	SLC35F1	
2693	E58	SLC35F5	
2694	E58	SLC38A4	
2695	E23	SLC38A8	
2696	E7	SLC39A2	
2697	E19	SLC3A1	
2698	E63	SLC3A2	
2699	E29	SLC40A1	
2700	E42	SLC40A1	
2701	E58	SLC40A1	
2702	E42	SLC44A4	
2703	E27	SLC44A5	
2704	E52	SLC45A3	

2705	E58	SLC45A4	
2706	E67	Slc4a4	
2707	E54	SLC4A7	
2708	E42	SLC5A1	
2709	E34	SLC6A1	
2710	E51	SLC6A1	
2711	E26	Slc6a11	
2712	E24	SLC6A12	
2713	E42	SLC6A20	
2714	E65	SLC6A7	
2715	E67	Slc7a1	
2716	E54	SLC7A1	
2717	E5	SLC7A11	
2718	E62	SLC7A11	
2719	E65	SLC7A2	
2720	E67	Slc7a2	
2721	E63	SLC7A5	
2722	E66	SLC7A5	
2723	E58	SLC8A1	
2724	E58	SLC9A2	
2725	E15	SLC9AB	
2726	E54	SLCO2B1	
2727	E21	SLIT1	
2728	E39	SLIT2	
2729	E70	Slit2	
2730	E1	SLIT3	
2731	E41	SLITRK6	
2732	E58	SLITRK6	
2733	E64	SLK	
2734	E43	SLN	
2735	E29	SLPI	
2736	E54	SLURP1	
2737	E41	SMAGP	
2738	E31	SMARCA2	
2739	E35	SMARCA4	
2740	E2	SMARCD3	
2741	E20	SMIM5	
2742	E2	SMO	
2743	E43	SNAI2	
2744	E54	SNAI2	
2745	E65	SNAI2	
2746	E63	SNAP23	
2747	E69	Snap25	

2748	E17	SNAP91	
2749	E49	Snap91	
2750	E66	SNHG3	
2751	E21	SNN	
2752	E43	SNORA12	
2753	E61	SNTA1	
2754	E2	SNX10	
2755	E37	SNX4	
2756	E59	Socs2	
2757	E33	SOCS3	
2758	E60	SOD3	
2759	E66	SOGA1	
2760	E66	SON	
2761	E27	SORCS2	
2762	E2	SORD	
2763	E53	SORD	
2764	E26	Sostdc1	
2765	E35	SOX11	
2766	E65	SOX11	
2767	E4	SOX2	
2768	E12	SOX2	
2769	E41	SOX2	
2770	E46	SOX2	
2771	E65	SOX2	
2772	E46	SOX2/3/8	
2773	E27	SOX2-OT	
2774	E46	SOX3/8	
2775	E33	SOX4	
2776	E24	SOX5	
2777	E65	SOX5	
2778	E24	SOX6	
2779	E59	Sox6	
2780	E46	SOX8	
2781	E24	SOX9	
2782	E51	SOX9	
2783	E56	SOX9	
2784	E2	SP5	
2785	E65	SP5	
2786	E2	SPAG5	
2787	E43	SPANXB1	
2788	E43	SPANXB2	
2789	E26	Sparc	
2790	E40	SPARC	

2791	E14	SPARC	
2792	E66	SPATA18	
2793	E2	SPATA24	
2794	E58	SPDEF	
2795	E54	SPDEF	
2796	E24	SPDYE1	
2797	E54	SPEF1	
2798	E66	SPEN	
2799	E59	Sphk1	
2800	E58	SPI1	
2801	E53	SPIB	
2802	E2	SPICE1	
2803	E33	SPINT2	
2804	E21	SPOCD1	
2805	E43	SPOCD1	
2806	E41	SPOCK1	
2807	E19	SPP1	
2808	E69	Spp1	
2809	E45	Spr2a3	
2810	E41	SPRED3	
2811	E14	SPRED3	
2812	E33	SPRY1	
2813	E49	Spry2	
2814	E69	Spry2	
2815	E51	SPRY4	
2816	E31	SPSB4	
2817	E26	Sptbn2	
2818	E67	Sqstm1	
2819	E66	SRCAP	
2820	E21	SRGAP1	
2821	E27	SRL	
2822	E2	SRL	
2823	E43	SRPX	
2824	E44	SSBP2	
2825	E21	SSTR3	
2826	E65	ST18	
2827	E21	ST3GAL5	
2828	E31	ST3GAL5	
2829	E42	ST6GALNAC1	
2830	E24	ST8SIA1	
2831	E16	STAC	
2832	E66	STAC	
2833	E50	STAM2	

2834	E59	Stap2	
2835	E32	STAR	
2836	E60	STARD4	
2837	E66	STARD4	
2838	E58	STARD8	
2839	E43	STC1	
2840	E22	STC2	
2841	E38	STC2	
2842	E54	STC2	
2843	E8	STEAP1	
2844	E54	STEAP1	
2845	E44	STEAP4	
2846	E54	STEAP4	
2847	E16	STK17A	
2848	E41	STK32B	
2849	E13	STK39	
2850	E63	STMN1	
2851	E17	STMN3	
2852	E17	STMN4	
2853	E27	STON1-GTF2A1L	
2854	E58	STX19	
2855	E59	Stxbp6	
2856	E3	SULT1C2	
2857	E42	SULT1C2	
2858	E32	SULT2A1	
2859	E68	SULT2A1	
2860	E24	SUZ12	
2861	E26	Sv2b	
2862	E1	SVEP1	
2863	E4	SYN2	
2864	E40	SYNE1	
2865	E41	SYNPO	
2866	E12	SYT1	
2867	E58	SYT1	
2868	E4	SYT2	
2869	E26	Tac1	
2870	E51	TAC1	
2871	E65	TAC4	
2872	E34	TACR2	
2873	E20	TACR3	
2874	E24	TAF13	
2875	E1	TAGLN	

2876	E40	TAGLN	
2877	E43	TAGLN	
2878	E70	Tagln	
2879	E14	TAP1	
2880	E31	TBC1D16	
2881	E62	TBC1D16	
2882	E17	TBC1D30	
2883	E56	TBC1D30	
2884	E65	TBX19	
2885	E23	TBX21	
2886	E24	TBXT	
2887	E41	TCAF2	
2888	E32	TCF21	
2889	E39	TCF4	
2890	E4	TCF7L1	
2891	E26	Tcf7l2	
2892	E27	TCF7L2	
2893	E24	TCFL2	
2894	E4	TDGF1	
2895	E67	Tdrd7	
2896	E2	TEAD2	
2897	E25	TENM1	
2898	E46	TERT	
2899	E42	TESC	
2900	E35	TFAP2B	
2901	E35	TFDP1	
2902	E14	TFE3	
2903	E22	TFF3	
2904	E58	TFF3	
2905	E8	TFPI2	
2906	E21	TFRC	
2907	E19	TG	
2908	E41	TGFA	
2909	E24	TGFB1	
2910	E16	TGFB1	
2911	E21	TGFB1	
2912	E70	Tgfb2	
2913	E70	Tgfb3	
2914	E67	Tgif1	
2915	E2	TGIF2	
2916	E29	TGM2	
2917	E33	TGM2	
2918	E43	TGM2	

2919	E59	Tgm2	
2920	E23	TGM4	
2921	E17	TH	
2922	E60	THBS1	
2923	E40	THBS2	
2924	E14	THNSL1	
2925	E1	THY1	
2926	E13	THY1	
2927	E27	TIAM1	
2928	E2	TIFA	
2929	E58	TIMD4	
2930	E2	TIMELESS	
2931	E14	TIMM50	
2932	E21	TIMP1	
2933	E31	TIRAP	
2934	E63	TK1	
2935	E70	Tk1	
2936	E31	TLCD1	
2937	E46	TLE1	
2938	E39	TLE4	
2939	E41	TLE4	
2940	E67	Tlr3	
2941	E41	TLR4	
2942	E42	TM4SF20	
2943	E65	TMBIM6	
2944	E42	TMC5	
2945	E58	TMC5	
2946	E14	TMC8	
2947	E15	TMED1	
2948	E1	TMEM125	
2949	E23	TMEM156	
2950	E43	TMEM158	
2951	E43	TMEM166	
2952	E31	TMEM170A	
2953	E31	TMEM170B	
2954	E29	TMEM176A	
2955	E49	Tmem176a	
2956	E29	TMEM176B	
2957	E49	Tmem176b	
2958	E26	Tmem212	
2959	E54	TMEM22	
2960	E26	Tmem30	
2961	E17	TMEM35	

2962	E65	TMEM38B	
2963	E8	TMEM45B	
2964	E15	TMEM59	
2965	E52	TMEM74	
2966	E26	Tmem88b	
2967	E2	TMEM9	
2968	E29	TMEM91	
2969	E15	TMEM98	
2970	E56	TMIE	
2971	E35	TMIGD2	
2972	E1	TMPRSS13	
2973	E31	TMPRSS5	
2974	E27	TMTC2	
2975	E21	TNC	
2976	E40	TNC	
2977	E57	TNC	
2978	E11	TNF	
2979	E38	TNFAIP3	
2980	E49	Tnfaip3	
2981	E66	TNFRSF10B	
2982	E42	TNFRSF11A	
2983	E8	TNFRSF12A	
2984	E21	TNFRSF12A	
2985	E28	TNFRSF13B	
2986	E28	TNFRSF13C	
2987	E21	TNFRSF14	
2988	E28	TNFRSF17	
2989	E21	TNFRSF19	
2990	E55	Tnfrsf19	
2991	E67	Tnfrsf1b	
2992	E47	TNFRSF21	
2993	E37	TNFRSF8	
2994	E14	TNFRSF9	
2995	E54	TNFRSF9	
2996	E2	TNFSF10	
2997	E44	TNFSF10	
2998	E54	TNFSF4	
2999	E67	Tnfsf9	
3000	E35	TNIK	
3001	E64	TNIK	
3002	E65	TNNI1	
3003	E58	TNNT1	
3004	E49	Tnnt2	

3005	E1	TNS1	
3006	E1	TNS4	
3007	E36	TNS4	
3008	E29	TOP2A	
3009	E63	TOP2A	
3010	E17	TOX	
3011	E37	TOX	
3012	E42	TOX3	
3013	E50	TP53	
3014	E43	TP53INP1	
3015	E60	TP63	
3016	E46	TP73	
3017	E60	TP73	
3018	E39	TPBG	
3019	E41	TPBG	
3020	E12	TPH1	
3021	E29	TPI1	
3022	E70	Tpk1	
3023	E41	TPM1	
3024	E70	Tpm1	
3025	E1	TPM2	
3026	E37	TPM2	
3027	E43	TPM2	
3028	E19	TPO	
3029	E60	TPO	
3030	E21	TPP1	
3031	E54	TPPP3	
3032	E37	TPST2	
3033	E65	TPT1	
3034	E14	TRAM2	
3035	E47	TRAM2	
3036	E58	TRDC	
3037	E26	Trf	
3038	E11	TRIAP1	
3039	E25	TRIB1	
3040	E69	Trib1	
3041	E49	Trib2	
3042	E69	Trib2	
3043	E24	TRIL	
3044	E7	TRIM29	
3045	E2	TRIM37	
3046	E4	TRIM71	
3047	E51	TRIM9	

3048	E67	Trip10	
3049	E12	TRIT1	
3050	E1	TRO	
3051	E16	TRPC1	
3052	E54	TRPC1	
3053	E15	TRPC6	
3054	E14	TRPM1	
3055	E34	TRPV4	
3056	E70	Trpv4	
3057	E7	TRPV6	
3058	E38	TRPV8	
3059	E60	TSC2	
3060	E33	TSC22D3	
3061	E18	TSFM	
3062	E57	TSGA10	
3063	E19	TSHR	
3064	E20	TSHZ1	
3065	E59	Tshz2	
3066	E65	TSHZ2	
3067	E68	TSPAN12	
3068	E43	TSPAN13	
3069	E69	Tspan13	
3070	E69	Tspan3	
3071	E18	TSPAN31	
3072	E15	TSPAN8	
3073	E42	TSPAN8	
3074	E63	TSPYL3	
3075	E33	TTC3	
3076	E35	TTC3	
3077	E26	Ttr	
3078	E51	TTYH1	
3079	E11	TUBA4A	
3080	E25	TUBB2B	
3081	E17	TUBB2B	
3082	E58	TUBB2B	
3083	E65	TUBB4A	
3084	E66	TUBB4A	
3085	E2	TXNDC16	
3086	E21	TXNIP	
3087	E33	TXNIP	
3088	E45	Txnip	
3089	E31	TYW1	
3090	E48	UAP1	

3091	E21	UAP1L1	
3092	E50	UBASH3B	
3093	E35	UBB	
3094	E67	Ube216	
3095	E14	UBE2E3	
3096	E14	UBE2H	
3097	E31	UBL3	
3098	E23	UCHL1	
3099	E34	UCN2	
3100	E45	Ugdh	
3101	E45	Ugt2b34	
3102	E23	UGT3A1	
3103	E14	ULBP2	
3104	E4	UNC13C	
3105	E20	UNC5A	
3106	E62	UNC5A	
3107	E17	UNC5C	
3108	E6	UNC5D	
3109	E46	UNCX	
3110	E2	UNG	
3111	E54	UPP1	
3112	E31	USB1	
3113	E42	USH1C	
3114	E62	VAMP7	
3115	E26	Vat1l	
3116	E2	VAV3	
3117	E26	Vcan	
3118	E16	VCAN	
3119	E43	VCAN	
3120	E56	VCAN	
3121	E21	VCL	
3122	E35	VCL	
3123	E2	VDR	
3124	E60	VDR	
3125	E29	VEGFA	
3126	E19	VEGFA	
3127	E54	VEGFA	
3128	E21	VGF	
3129	E21	VGLL4	
3130	E33	VGLL4	
3131	E42	VIL1	
3132	E16	VIM	
3133	E66	VIM	

3134	E65	VIPR1	
3135	E1	VIT	
3136	E31	VKORC1L1	
3137	E31	VMAC	
3138	E29	VMP1	
3139	E30	VPS9D1-AS1	
3140	E32	VSNL1	
3141	E40	VSNL1	
3142	E65	VSTM2A	
3143	E52	VTCN1	
3144	E66	VWA1	
3145	E58	VWA2	
3146	E54	VWA5A	
3147	E66	VWA5B2	
3148	E56	VWDE	
3149	E26	Vxn	
3150	E27	WASF3	
3151	E4	WDFY2	
3152	E14	WDR43	
3153	E16	WDR47	
3154	E68	WDR63	
3155	E23	WDR72	
3156	E58	WDR72	
3157	E2	WDR90	
3158	E70	Wee1	
3159	E25	WFDC1	
3160	E2	WFDC15B	
3161	E29	WFDC2	
3162	E70	Wisp1	
3163	E54	WISP2	
3164	E70	Wisp2	
3165	E65	WNT16	
3166	E46	WNT3A	
3167	E39	WNT5A	
3168	E46	WNT5A	
3169	E54	WNT6	
3170	E58	WNT6	
3171	E40	WNT7A	
3172	E49	Wnt7a	
3173	E46	WNT7B	
3174	E10	WRN	
3175	E37	WSB2	
3176	E70	Wwc1	

3177	E24	WWP2	
3178	E37	XBP1	
3179	E60	XBP1	
3180	E47	XBP1	
3181	E58	XCL2	
3182	E66	XIST	
3183	E14	XKRX	
3184	E24	XYLB	
3185	E35	YAP1	
3186	E54	YARS	
3187	E18	YEATS4	
3188	E33	YPEL3	
3189	E58	YWHAEP7	
3190	E37	ZA20D3	
3191	E35	ZADH2	
3192	E37	ZAP70	
3193	E54	ZBED2	
3194	E65	ZBED2	
3195	E67	Zbp1	
3196	E37	ZBT20	
3197	E2	ZBTB12	
3198	E26	Zbtb18	
3199	E31	ZBTB41	
3200	E35	ZBTB7A	
3201	E21	ZCCHC12	
3202	E33	ZCCHC24	
3203	E62	ZCCHC3	
3204	E39	ZEB1	
3205	E33	ZFAND5	
3206	E66	ZFHX3	
3207	E2	ZFP566	
3208	E65	ZFPM2	
3209	E26	Zic1	
3210	E65	ZIC2	
3211	E46	ZIC5	
3212	E65	ZIC5	
3213	E2	ZKSCAN17	
3214	E43	ZMAT3	
3215	E66	ZMAT3	
3216	E23	ZMAT4	
3217	E65	ZMAT4	
3218	E66	ZMIZ1	
3219	E54	ZMYND10	

3220	E27	ZMYND8	
3221	E60	ZNF185	
3222	E65	ZNF385B	
3223	E31	ZNF396	
3224	E66	ZNF428	
3225	E65	ZNF536	
3226	E14	ZNF704	
3227	E35	ZNF704	
3228	E65	ZNF750	
3229	E14	ZNF75D	
3230	E61	ZNF791	
3231	E65	ZNF804A	
3232	E4	ZSCAN10	
3233	E21	ZYX	
3234	E43	ZYX	
3235	E26	1110017D15Rik	
3236	E45	1700030C14Rik	
3237	E45	1700031L13Rik	
3238	E26	1700094D03Rik	
3239	E26	2900040C04Rik	
